# Comparative Analysis of the Recently Discovered *hAT* Transposon *TcBuster* in Human Cells

**DOI:** 10.1371/journal.pone.0042666

**Published:** 2012-11-15

**Authors:** Lauren E. Woodard, Xianghong Li, Nirav Malani, Aparna Kaja, Robert H. Hice, Peter W. Atkinson, Frederic D. Bushman, Nancy L. Craig, Matthew H. Wilson

**Affiliations:** 1 Department of Medicine, Division of Nephrology, Baylor College of Medicine, Houston, Texas, United States of America; 2 Department of Entomology & Institute for Integrative Genome Biology, University of California Riverside, Riverside, California, United States of America; 3 Howard Hughes Medical Institute, Department of Molecular Biology and Genetics, Johns Hopkins School of Medicine, Baltimore, Maryland, United States of America; 4 Department of Microbiology, Perlman School of Medicine at the University of Pennsylvania, Philadelphia, Pennsylvania, United States of America; 5 Michael E. DeBakey Veteran's Administration Medical Center, Houston, Texas, United States of America; New England Biolabs, Inc., United States of America

## Abstract

**Background:**

Transposons are useful tools for creating transgenic organisms, insertional mutagenesis, and genome engineering. *TcBuster*, a novel hAT-family transposon system derived from the red flour beetle *Tribolium castaneum*, was shown to be highly active in previous studies in insect embryoes.

**Methodology/Principal Findings:**

We tested *TcBuster* for its activity in human embryonic kidney 293 (HEK-293) cells. Excision footprints obtained from HEK-293 cells contained small insertions and deletions consistent with a *hAT*-type repair mechanism of hairpin formation and non-homologous end-joining. Genome-wide analysis of 23,417 *piggyBac*, 30,303 *Sleeping Beauty*, and 27,985 *TcBuster* integrations in HEK-293 cells revealed a uniquely different integration pattern when compared to other transposon systems with regards to genomic elements. *TcBuster* experimental conditions were optimized to assay *TcBuster* activity in HEK-293 cells by colony assay selection for a neomycin-containing transposon. Increasing transposon plasmid increased the number of colonies, whereas gene transfer activity dependent on codon-optimized transposase plasmid peaked at 100 ng with decreased colonies at the highest doses of transposase DNA. Expression of the related human proteins Buster1, Buster3, and SCAND3 in HEK-293 cells did not result in genomic integration of the *TcBuster* transposon. *TcBuster*, *Tol2*, and *piggyBac* were compared directly at different ratios of transposon to transposase and found to be approximately comparable while having their own ratio preferences.

**Conclusions/Significance:**

*TcBuster* was found to be highly active in mammalian HEK-293 cells and represents a promising tool for mammalian genome engineering.

## Introduction

Transposon technology has been harnessed for genome engineering for the creation of transgenic organisms [1], cancer gene discovery by insertional mutagenesis [2], and in pre-clinical gene transfer experiments by inserting genes into the genomes of the somatic cells of living organisms [3]. While the most studied transposon for these applications has been *Sleeping Beauty*, a reconstructed Tc1/*mariner*-type transposon from fish [4], others such as *Frog Prince* [5], *Tol2* [6], and *piggyBac* [7] have also proven to be highly active in diverse organisms and applications. Although they share a similar overall mechanism of “cut and paste” of the delivered transposon DNA, each transposon system has its own characteristics. For example, *Sleeping Beauty* is subject to loss of activity when attached to protein domains intended to direct transposition to certain DNA sequences [8]–[10]. *piggyBac* has a higher affinity for integrating near genes as compared with *Sleeping Beauty* [11] and appears more amenable to the addition of protein domains or tags [8], [12]. Importantly, transposon systems are known to differ in their target site preference and these differences have been exploited for expanding cancer gene discovery [13]. Thus, exploring the unique features of newly discovered transposon systems is important to provide more choices for mammalian genome engineering.

The *hAT* superfamily of DNA transposons are found in diverse species, as indicated by its name which is derived from the *hobo* transposon from *Drosophila* [14], the *Ac* transposon from maize [15], and the *Tam3* transposon from snapdragon [16]. *hAT* transposons are also found in many animals, including inactive and domesticated forms of *hAT* elements in the human genome [17]. The *hAT* superfamily can be further divided into two subfamilies, the *Buster* family, containing the *Buster* and *SPIN* transposons, and the *Ac* family, containing the *Activator*, *Tam3*, *hobo*, *Hermes*, and *Tol2* transposons [18].

Previous studies have shown that the *Ac*-family transposons are not only found in diverse species across many kingdoms of life [19], but that some *Ac*-family transposons remain fully functional when used for genetic engineering in species quite unrelated to their genome of origin. For example, *Activator* was able to transpose the maize *Dissociation* element in the germline of the zebrafish *Danio rerio*, displaying accurate and efficient germline transmission along with large-fragment carrying capacity and high levels of reporter gene expression [20]. Another example of the cross-species utility of the *Ac*-family elements was the successful *Tol2*-mediated transgenic manipulation of zebrafish, *Xenopus*, and mouse [21]–[23]. *Tol2* is also active in human tissue culture cells and in mouse liver, and was successfully used for gene therapy of hereditary tyrosemia type 1 in a mouse model [6], [21].

Recent bioinformatic technology has increased our ability to search genomes for transposon sequences and a number of new genome sequences are published each year. The *TcBuster* transposon was recently identified by searching all available sequence using bioinformatics to find active *hAT* family transposons [18]. *TcBuster* is named for the species from which it was isolated, *Tribolium castaneum* or the red flour beetle [24], and its similarity to a group of domesticated transposase genes discovered in the human genome, *Buster*-1–4 [25]. *TcBuster* is highly active in insect S2 cells and in the embryos of the insects *D. melanogaster* and *Ae. aegypti* [18]. Additionally, *TcBuster* is able to transpose efficiently *in vitro*, in *Saccharomyces cerevisiae*, and mammalian cells [26]. We aimed to further explore the use of *TcBuster* for genetic manipulation of mammalian cells by optimizing its activity in human HEK-293 cells. *TcBuster* was found to have slightly different human genomic integration preferences when compared to *Sleeping Beauty* and *piggyBac* both in concurrent studies done in HeLa cells [26] and in data shown here for HEK-293 cells which should expand the arsenal of transposon systems for genomic applications. Additionally, in this study we uncovered novel characteristics in the dose-response curve of the *TcBuster* as compared with *hAT* transposon *Tol2* that will inform future studies of the *hAT* transposons.

## Results

We compared *TcBuster* to three other transposons in various assays: *Sleeping Beauty* (for integration preferences), *piggyBac* (for integration preferences and activity), and *Tol2* (for activity). The transposons used in this study each contained an identical cassette encoding for a drug-resistance gene, so that the transfected cells could be selected with either neomycin (activity assays: pTcBNeo for *TcBuster*, pTpB for *piggyBac*, or pTol2Neo for *Tol2*) or blasticidin (integration preferences: pXL-TcB-D-GFP/Bsd for *TcBuster*, pXL-PB-D-GFP/Bsd for *piggyBac*, or pXL-SB-D-GFP/Bsd for *Sleeping Beauty*) to select for the sustained expression of the transgene. In each case we expressed the transposase from a separate helper plasmid (pCMV-*TcBuster*, pCMV-*piggyBac*, or pCMV-*Tol2* for activity and pXL-CMV-TcBuster_CO_, pXL-CMV-piggyBac, or pXL-CMV-Sleeping Beauty for integration preferences). After selection for two weeks, each cell that survived the drug selection formed a colony, which was counted so that the transposon systems could be compared.

### *TcBuster* excision activity and footprint in HEK-293 cells

**Figure 1:**
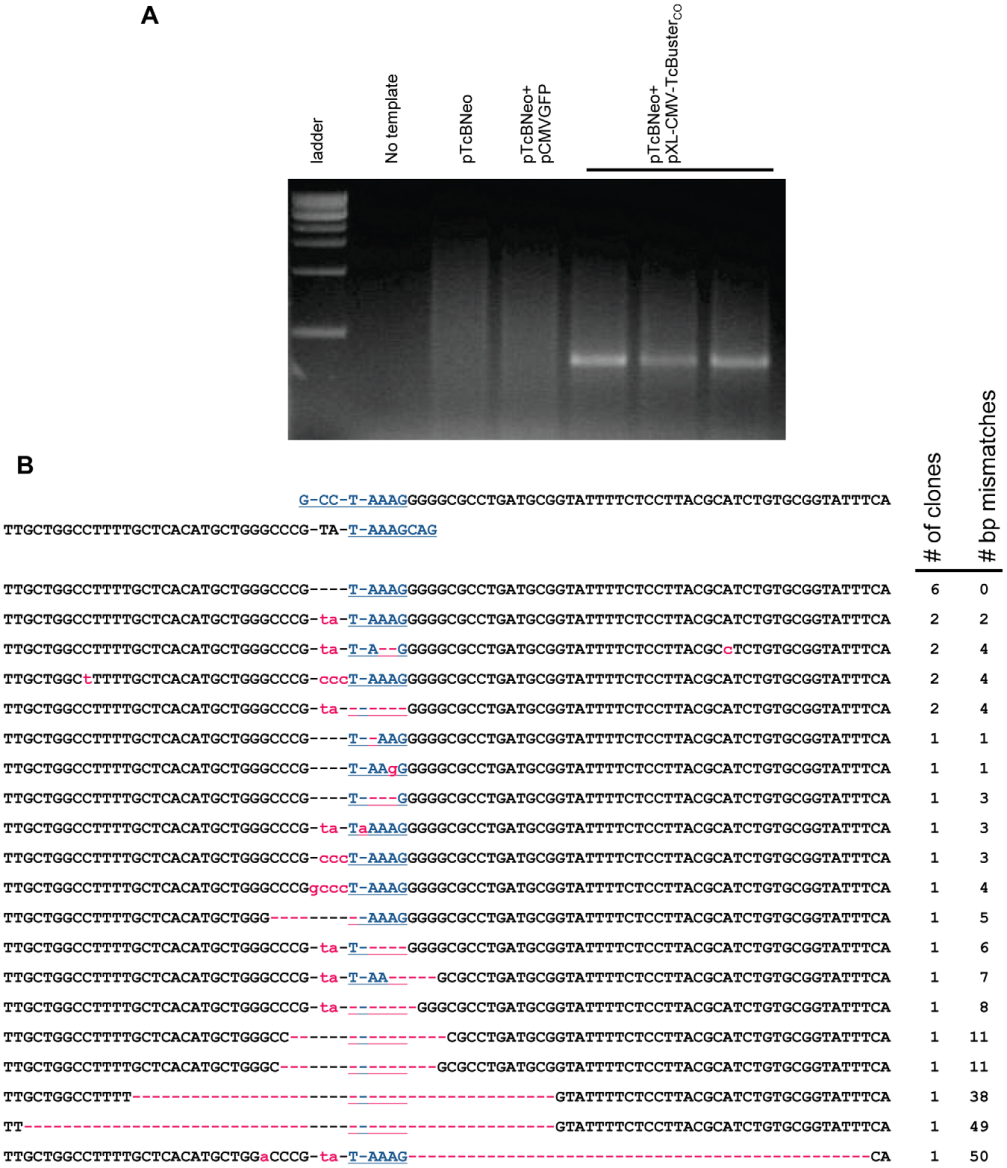
Transposase-mediated excision of the *TcBuster* transposon in HEK-293 cells. (**a**) An agarose gel of the excision PCR. Plasmid DNA was extracted from transfected HEK-293 cells and used as a template for nested PCR to detect the excision of the transposon DNA. Lane 1, 1 kb ladder; lane 2, PCR reaction without any DNA template added; lanes 3–7, PCR on extracts from cells transfected with either 1 µg of the transposon plasmid pTcBNeo (lane 3), 867 ng transposon pTcBNeo and 133 ng pCMVGFP negative control (lane 4), or 867 ng transposon pTcBNeo and 133 ng pXL-CMV-TcBuster_CO_ transposase plasmid (three separate transfections, lanes 5–7). (**b**) The three PCR bands shown in (**a**) were gel-purified and TOPO-cloned. Clones were sequenced to determine the exact excision junction. The sequence flanking the transposon in pTcBNeo is shown at the top. The TAAAG homology region is shown in blue. Mismatches are shown in lowercase pink. Dashes are used to maintain alignment and if pink, indicate a missing bp. The sequences are ranked according to (1) incidence (# of clones) followed by (2) number of bp not matching the highest incidence clone (# bp mismatches).

An excision assay was developed to confirm the activity of our *TcBuster* constructs and to determine its excision footprint in human cells. The transposase-producing plasmid was compared to a control plasmid (pCMVGFP) expressing green fluorescent protein (GFP) from the CMV promoter. HEK-293 cells were transfected with target plasmid pTcBNeo and either the transposase helper plasmid pXL-CMV-TcBuster_CO_ or negative control. Plasmid DNA was extracted from the cells twenty-four hours later and subject to a nested PCR reaction. The PCR produced a ∼450 bp product only if the transposon was excised from the plasmid pTcBNeo by *TcBuster* transposase (**Fig. 1a**).

To determine the excision footprint of *TcBuster* in HEK-293 cells, the PCR band was excised from the gel and TOPO-cloned. The sequenced clones revealed that the insertion of a few nucleotides was common, and in a few cases deletions at the excision site of up to 50 bp were found (**Fig. 1b**). This is consistent with excision footprint sequences that have been reported for other *hAT* transposons [27]–[29], including that of *TcBuster* in *Aedes aegypti* embryos [18]. As is the case for the other *hAT* transposons, the additional nucleotides and deletions are attributable to the formation of hairpin structures on the DNA ends that are left in the plasmid following excision of the transposon, that are then repaired by non-homologous end-joining [30]. Insertions of “ccc” and “gccc” are complementary to the “GGGC” sequence after the “TAAAGG” (**Fig. 1b**), so there is direct evidence for this mechanism for *TcBuster*.

### *TcBuster-*mediated gene transfer in HEK-293 cells

**Figure 2:**
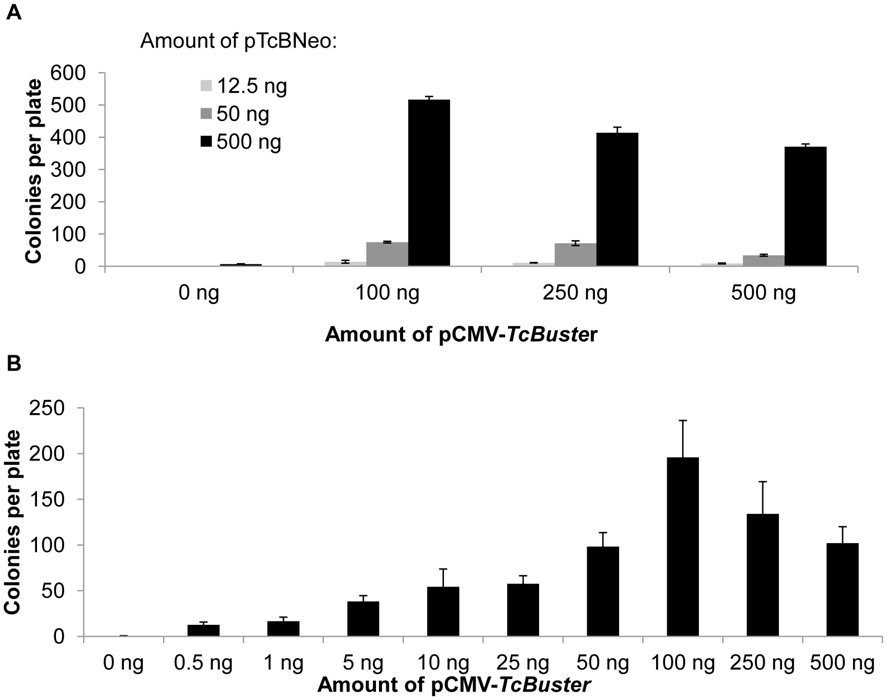
The effect of transposon and transposase plasmid dose on the number of drug-resistant colonies formed. (**a**) HEK-293 cells in 6-well plates were transfected in triplicate with either 12.5 ng (light grey bars), 50 ng (dark grey bars), or 500 ng (black bars) of pTcBNeo carrying the neomycin-resistance transposon and 0 ng, 100 ng, 250 ng, or 500 ng of pCMV-*TcBuster* expressing the transposase. (**b**) HEK-293 cells in 6-well plates were transfected in triplicate with 500 ng of pTcBNeo plasmid carrying the neomycin-resistance transposon and the indicated amount of pCMV-*TcBuster* (0.5 ng, 1 ng, 5 ng, 10 ng, 25 ng, 50 ng, 100 ng, 250 ng, or 500 ng). In both **a** and **b**, pUC19 was used as filler DNA to increase the total amount of DNA transfected to 1 µg. Cells were diluted 1∶750 in selection media and grown for two weeks to allow drug-resistant cells to multiply and form colonies. The colonies were fixed, stained, and counted. The mean and standard error of the mean (SEM; n = 3) are shown.

We varied the amount of *TcBuster* transposon (pTcBNeo) and transposase plasmid DNA (pCMV-*TcBuster*) in order to optimize the *TcBuster* system for long-term gene expression in mammalian cells. HEK-293 cells were transfected with either 12.5 ng, 50 ng, or 500 ng of pTcBNeo and either 0 ng, 100 ng, 250 ng, or 500 ng of pCMV-TcBuster, with enough pUC19 filler plasmid DNA added to reach a total of 1000 ng per transfection. By colony assay for drug-resistant cells, we found that there was a linear relationship between the amount of transposon DNA added and transposition (**Fig. 2a**). In contrast, the relationship between transposase plasmid amount and colony number was not linear (**Fig. 2a**). To further investigate the extent of the transposase plasmid dosage effect, HEK-293 cells were transfected with 500 ng of pTcBNeo and varying amounts of pCMV-*TcBuster* transposase plasmid plus enough pUC19 to equal 1 µg of total plasmid DNA (**Fig. 2b**). The population of cells transfected with 0.5 ng of pCMV-*TcBuster* produced significantly more colonies than cells transfected with transposon alone. The number of colonies and presumably the number of genomes carrying the drug-resistance transposon increased up to the 100 ng pCMV-*TcBuster* dose and then declined with the same behavior as in **Fig. 2a**.

**Figure 3:**
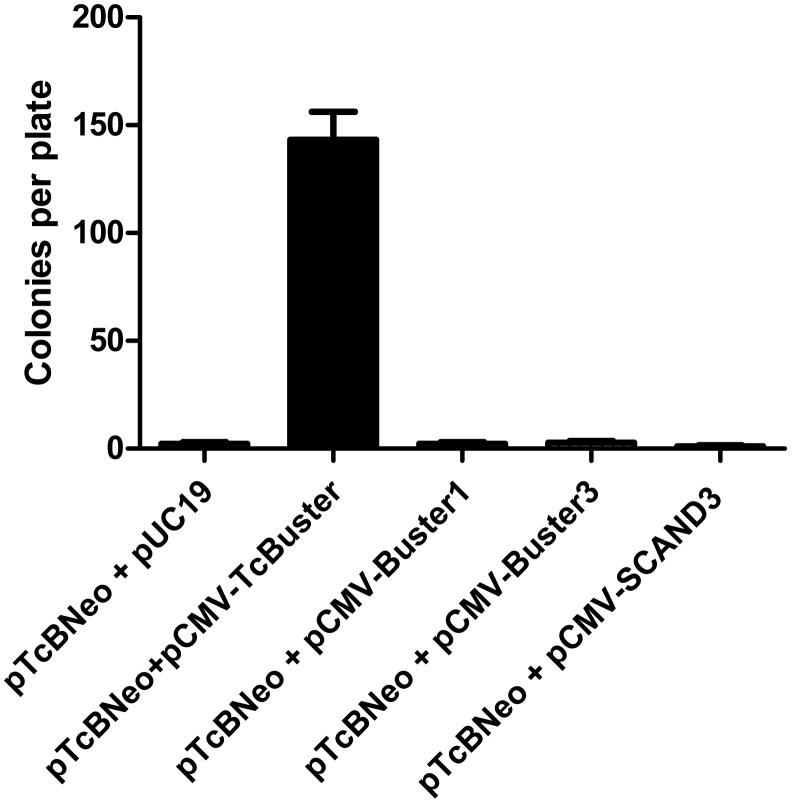
The *TcBuster* transposase-related human proteins Buster1, Buster3 and SCAND3 cannot insert the *TcBuster* transposon into the mammalian genome. HEK-293 cells were split onto 60 mm dishes and transfected with 1800 ng of pTcBNeo and 200 ng of *Buster* plasmid using FuGene6. The transfected cells (10 µl) were diluted into media containing G418 in 10 cm dishes and allowed to grow for two weeks. The resulting colonies were stained and counted (n = 3; error bars represent the standard error of the mean).

We also tested three human *Buster* proteins [25] for their ability to mobilize the TcBuster transposon (**Fig. 3**). When the CMV promoter was used to express Buster1, Buster3, or related protein SCAND3 in the presence of pTcBNeo in HEK-293 cells, colony numbers were equal to background levels of random integration, suggesting a potential lack of cross-reactivity between the *TcBuster* system and the human *Buster* transposase-related genes.

### Genome-wide analysis of *TcBuster* integration in human cells

**Figure 4:**
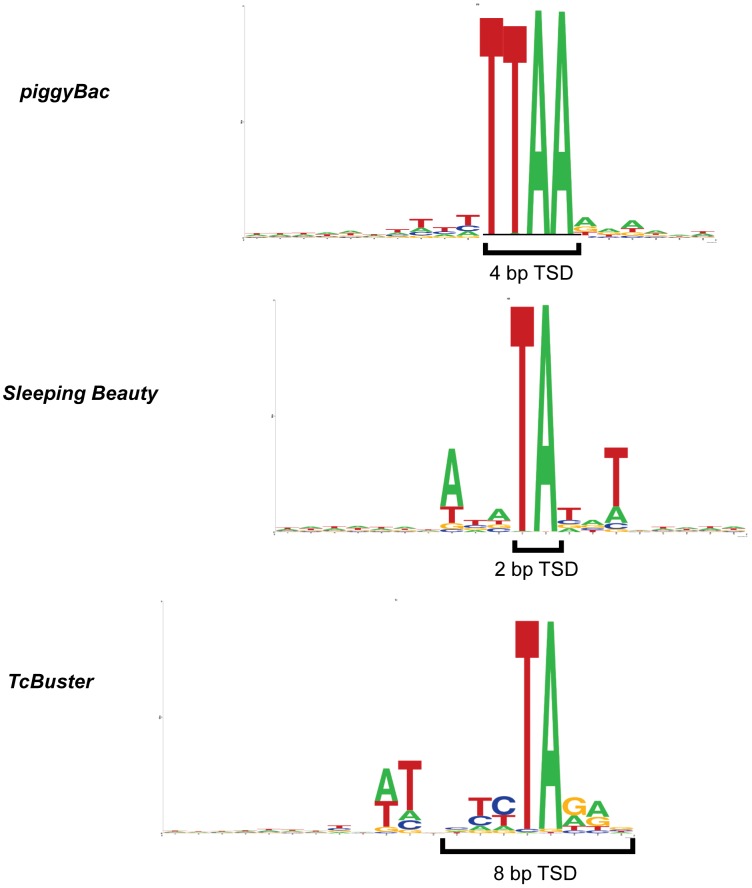
WebLogos generated from integration sites of *piggyBac*, *TcBuster*, and *Sleeping Beauty* transposons in HEK- 293 cells using the software at weblogo.berkeley.edu **.**

*TcBuster* integration has previously been shown to produce 8bp target site duplications with the consensus sequence nnnTAnnn in insects [18], [31]. We isolated and sequenced a large number of *TcBuster* transposon integrations in HEK-293 cells using ligation-mediated PCR to capture transposon-chromosomal junctions. *Sleeping Beauty* and *piggyBac* are the two most characterized transposon systems used in mammalian cells. Therefore, we compared *TcBuster* integrations to large numbers of *Sleeping Beauty* and *piggyBac* integrations. Overall, we analyzed 23,417 *piggyBac*, 30,303 *Sleeping Beauty*, and 27,985 *TcBuster* integrations in HEK-293 cells (**Table 1**). These large collections of integrations, which are the largest that have been analyzed for *TcBuster*, permitted thorough characterization of integration site preference with regards to sequence and genomic elements. **Fig. 4** shows WebLogos comparing the consensus target site for *Sleeping Beauty*, *piggyBac*, and *TcBuster* in HEK-293 cells, which were similar to those produced from integrations in HeLa cells [26].

**Table 1:** The distribution of nucleotide sequences found at transposon insertion sites in HEK-293 cells.

*piggyBac*		*TcBuster*		*Sleeping Beauty*	
Total	23417	Total	27985	Total	30303
TTAA	23026	TA	26871	TA	30102
CTAA	110	CA	394	AT	43
TTAG	100	TG	373	AA	39
ATAA	30	TT	129	CA	37
TCAA	29	AA	109	TG	20
TTGA	28	TC	31	GA	15
GTTA	25	GA	17	TT	11
TTAT	18	AG	17	TC	8
GTAA	10	CT	14	AG	7
CTAG	9	AT	10	AC	7
TAAA	6	GT	9	GG	3
GTAT	3	GG	5	GC	3
TTAC	3	CC	4	GT	2
TTTA	3	AC	2	CT	2
AAGA	2			CG	2
AGGC	2			CC	2
ATAC	2				
AAAC	1				
ACAG	1				
ACGA	1				
AGTC	1				
ATAT	1				
CCCT	1				
CGTA	1				
GCTA	1				
TGAA	1				
TTCT	1				
TTTT	1				

**Figure 5:**
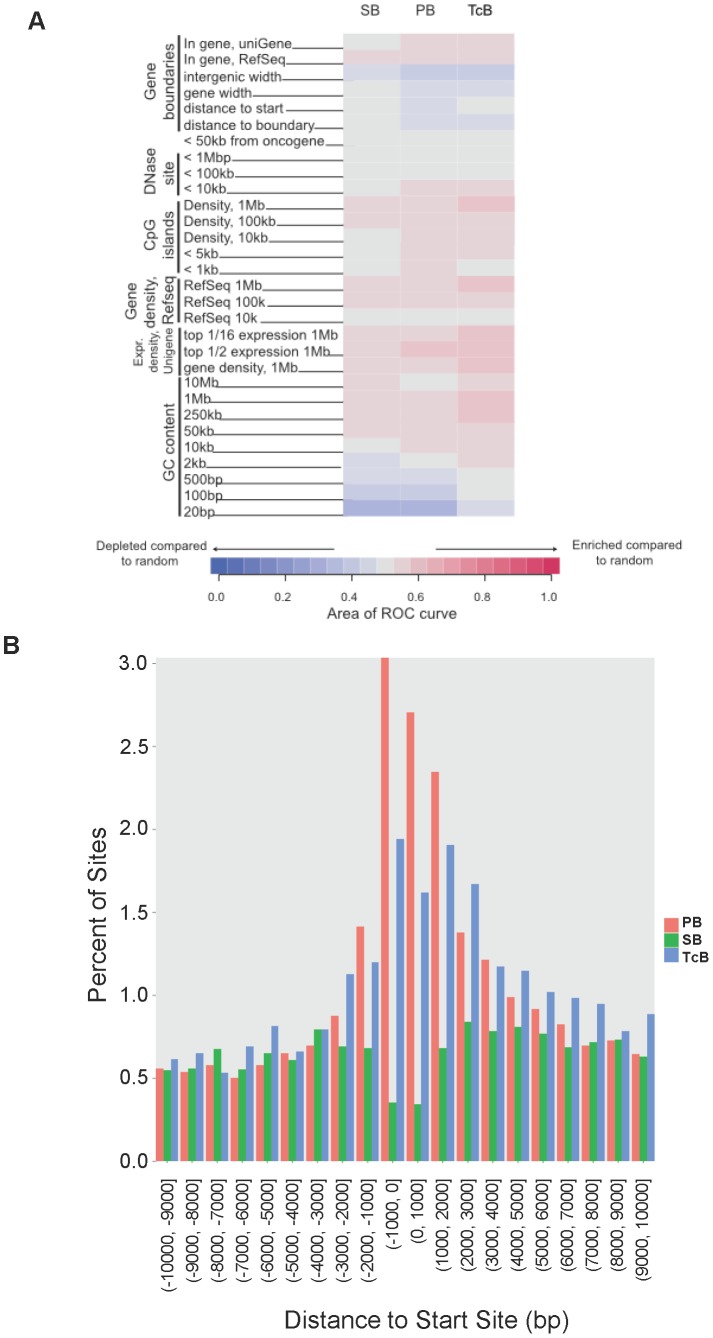
High-throughput sequencing of transposon integration sites in HEK-293 cells. (**a**) Integration frequency near selected genomic features. Integration site data sets for each transposon are indicated by the columns and the distance from the integration site to the genomic features by the rows. The departure from random distribution is indicated by colored tiles. Blue indicates that insertions are depleted compared to random whereas red indicates features where insertions are enriched compared to random and gray indicates that the distribution is random. Differences from random placement were scored using the ROC area method. (**b**) Integration frequency of *Sleeping Beauty*, *piggyBac*, and *TcBuster* near transcription start sites. Integration sites near transcription start sites were compiled onto a common transcription start site and the proportions mapped. The x-axis shows the distance from the transcription start sites and the y-axis shows the percentage of integration sites that were found for each range of distances.

We analyzed the genome-wide integration distributions of *Sleeping Beauty*, *piggyBac*, and *TcBuster* in HEK-293 cells and compared them with genomic elements such as annotated transcription units (**Fig. 5**). For comparison, we also generated a set of Matched Random Control (MRC) sites *in silico* as described previously [32]. Transposon integration relative to genomic features is summarized in heat maps using the ROC area method for comparison (**Fig. 5**) [32].

*piggyBac* and *TcBuster* showed weak favoring of integration in genomic regions enriched in transcription units, CpG islands, and preferential cleavage sites for DNase I (**Fig. 5a**). *Sleeping Beauty* had a lower preference for integration near these features. All three transposons showed favored integration near genes that were active in HEK-293 cells based on comparison to transcriptional profiling data for HEK-293 cells (**Fig. 5a**, labeled “Expr. Density”). *piggyBac* and *TcBuster* also showed increased integration frequency near transcription start sites, whereas *Sleeping Beauty* disfavored integration near transcription start sites (**Fig. 5b**). *Sleeping Beauty* had a greater preference for integration within L1 repeats and simple repeats compared with *piggyBac* and *TcBuster* (**Fig. S1A**). *Sleeping Beauty* also had a preference for integration within 50 kb of acro repeats (**Fig. S1B**). Interestingly, *TcBuster* had the same preference for integration into and near to *Ac-hobo* repeats as *piggyBac* and *Sleeping Beauty*, so we found no evidence of an affinity of *TcBuster* toward the sequences of related transposon fossils (**Fig. S1**). Further sequencing analysis including chromosomal preferences, preferences for introns and exons, graphs detailing short distances to genomic features, and more are available in the attached Berry report (**Fig. S2**). These results from HEK-293 cells are similar to those obtained using HeLa cells [26].

### *TcBuster*-mediated gene transfer in human cells is comparable to *piggyBac* and *Tol2*

**Figure 6:**
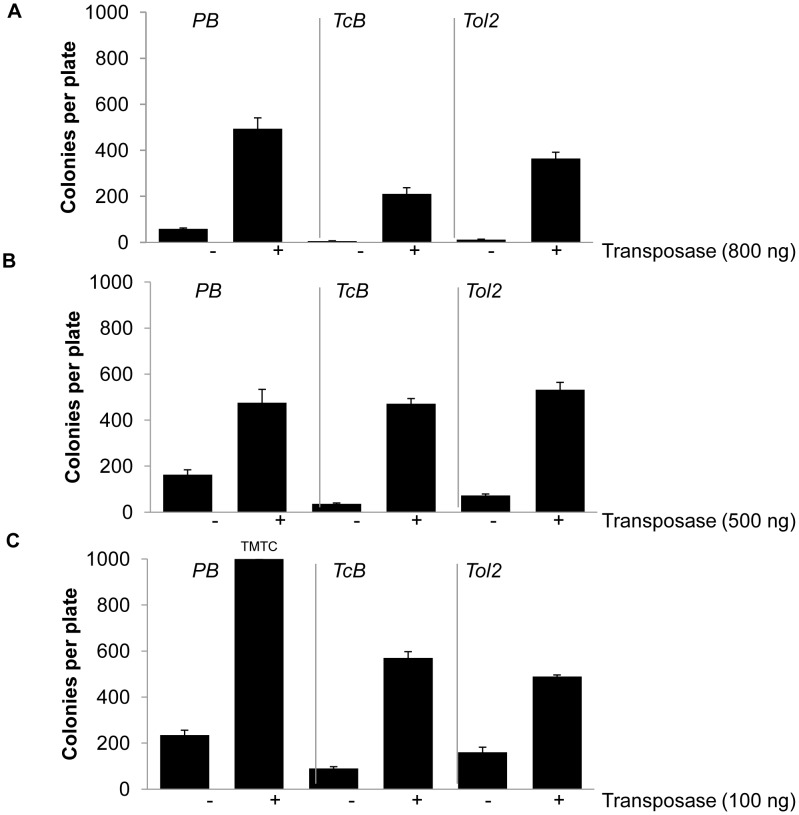
Comparison of *piggyBac*, *TcBuster*, and *Tol2* at varying transposase: transposon ratios. HEK-293 cells in 6-well plates were transfected in triplicate with the indicated amount of transposase helper plasmid (+) or pUC19 (−) and plasmids carrying the neomycin-resistance transposon for each system. The amount of transposon plasmid transfected was 200 ng (**a**), 500 ng (**b**), or 900 ng (**c**). In **c**, the *piggyBac* (+) transposase plates had approximately 1000 colonies, too many to count (TMTC). Cells were diluted 1∶750 onto 10 cm plates in media containing geneticin for selection and incubated for two weeks to allow drug-resistant cells to form colonies that were fixed, stained and counted. Error bars represent the SEM (n = 3).

To gain further insight into how *TcBuster* compares to other transposons, we chose the transposons *piggyBac* and *Tol2* for comparison to *TcBuster*. We choose these particular transposons for simplicity as *piggyBac* is a highly active transposon system that is comparable to *Sleeping Beauty* [7], [33] and *Tol2* is the only other *hAT* superfamily transposon that has been used for genetic manipulation of murine somatic cells [6]. Due to differences in the dose-response kinetics of *TcBuster* (**Fig. 2**) and those reported for *Tol2* [6], we tested three ratios of transposase to transposon: 4∶1, 1∶1, or 1∶9. When an excess of transposase plasmid DNA was present, *piggyBac* and *Tol2* both yielded more colonies than *TcBuster* (t-test, p = 0.006 and p = 0.02, respectively) although they were not significantly different from each other (**Fig. 6a**). When equal amounts of transposase and transposon plasmids were transfected, the three systems performed comparably (**Fig. 6b**). Transfection of a nine-fold excess of transposon over transposase plasmid gave a number of colonies for *piggyBac* that was too high to quantify, approximately 1000 per 10 cm plate for each replicate (**Fig. 6c**). *TcBuster* produced more colonies than *Tol2* in this condition (p = 0.04). *piggyBac* was equal to or better than both *hAT* transposons at each ratio tested. Comparing the *hAT* transposons, *TcBuster* and *Tol2* appeared to have opposite behavior. While *TcBuster* produced more colonies than *Tol2* at the 9∶1 transposon to transposase plasmid ratio, *Tol2* outperformed *TcBuster* at the 1∶4 ratio. Therefore, we conclude that *piggyBac*, *TcBuster* and *Tol2* are all highly active in human HEK-293 cells, but these transposons each have unique dose-response behavior.

**Figure 7:**
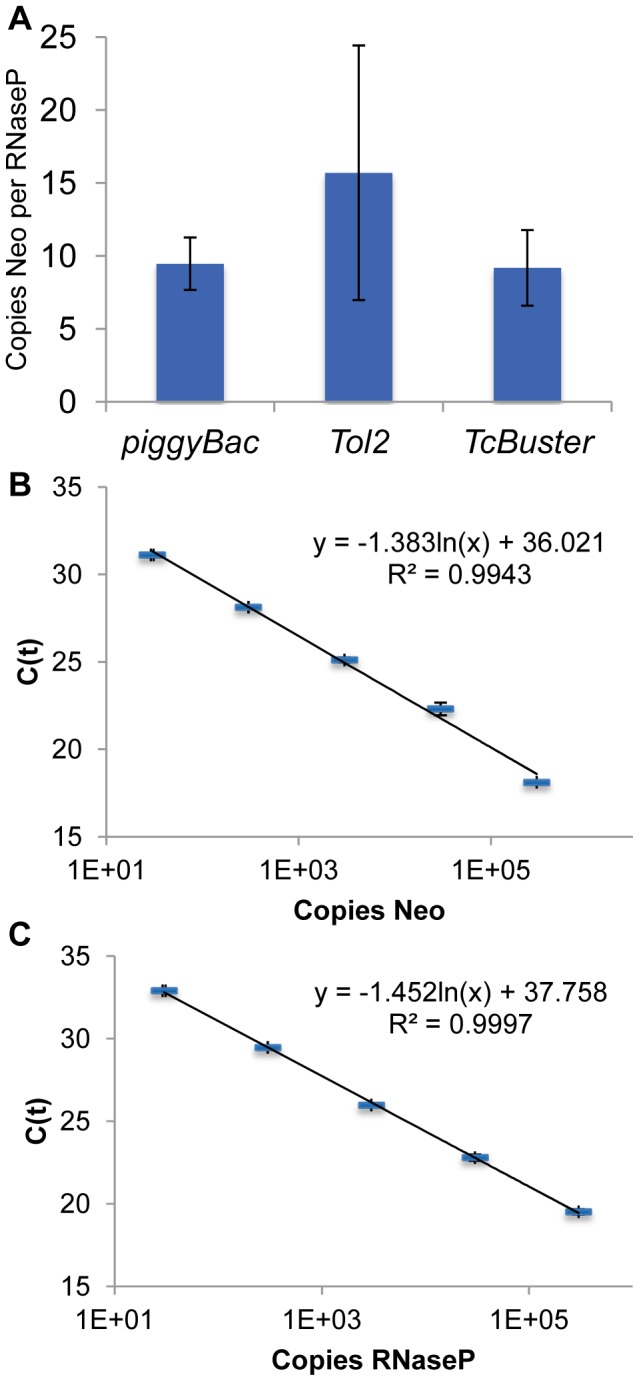
Transposon copy number analysis by real-time quantiative PCR (qPCR) for *piggyBac*, *TcBuster*, and *Tol2*. (**a**) The number of copies of the transposon (*Neo*) per haploid genome (copy of *RNaseP*). There was no statistically significant difference between the transposons by the ANOVA test. Propagation of errors was used to combine errors inherent in the plates per group and replicates per plate. Error bars represent the standard error of the mean. (**b**) Standard curve showing the C(t) result vs number of copies of pTpB added. (**c**) Standard curve showing the C(t) result vs number of copies of pRNaseP added. Both sets of standards were performed in triplicate for each dilution. The equation for the line of best fit and R squared value are printed on each graph. The error bars represent the standard error of the mean.

Although colony assays are a common method to determine transpositional activity, they are limited in the sense that only integration events that result in long-term expression of the drug resistance gene on the transposon will actually result in the formation of a colony, so cells receiving only integration event(s) that were silenced will not form colonies that can be counted. Colony assays also are not informative with regard to the number of integrated transposons. Therefore, we also performed real-time quantitative PCR (qPCR) to determine the average number of transposons integrated per cell (**Fig. 7**). HEK-293 cells were transfected and selected as for a colony assay. The cells were transfected with 100 ng of transposase as this was optimal for *TcBuster* (**Fig. 6**) and 500 ng of transposon. We used quantification of the neomycin resistance gene (*Neo*) rather than the *IR* sequences because the *Neo* sequence was the same for all transposon groups, whereas amplification of the *IR* sequences would require different reactions for each transposon. In order to determine the number of transposons per genome, we performed qPCR for both *Neo* and the human *RNaseP* gene and derived the copy number in each reaction from the qPCR C(t) result by fitting the data to line-of-best-fit equations that were obtained by a dilution series of either pTpB for *Neo* or pRNaseP for *RNaseP* (**Fig. 7b, c**). Dividing the number of copies of the neomycin resistance gene by the number of copies of *RNaseP* gave the average number of copies of the transposon per haploid genome. By multiplying the result shown in **Fig. 7a** by two, one arrives at the number of copies of transposon per diploid genome of approximately 20–30 transposons per cell. We have previously found that *piggyBac* produced transposon copy numbers within this range under similar experimental conditions [8], [33]. We observed no significant difference between *TcBuster*, *Tol2*, or *piggyBac* with regard to transposon copy number. Therefore, comparisons between the transposons with regard to transpositional activity can be accurately approximated from the colony count assay.

## Discussion

In this study, we optimized and characterized aspects of the *TcBuster* transposon for the permanent integration of genes into human genomes. Sequencing of a large number of *TcBuster* integrations into HEK-293 cells revealed a different integration pattern and preference when compared to the other most commonly used systems, *Sleeping Beauty* and *piggyBac*. By colony assay, *TcBuster* was comparable to other transposon systems and displayed a slightly negative dose-response when an excess of transposase plasmid was present. Together, our data support the addition of *TcBuster* transposase to the group of enzyme systems available for the permanent addition of genes to mammalian genomes.

Plasmids from which *TcBuster* transposons were excised in HEK-293 cells were PCR amplified, TOPO-cloned, and sequenced (**Fig. 1**). Many of the sequences included insertions of a few nucleotides of DNA complementary to the plasmid DNA template and some had deletions of up to 50 bp. The sequences in **Fig. 1a** are consistent with sequences of the transposition junctions from insect embryos [18]. Our excision footprints support a transposition mechanism involving hairpin formation at the donor plasmid ends following transposon excision that are repaired by non-homologous end-joining [30]. This result is expected for *TcBuster* since the other *hAT* transposons have similar errors in their footprints [27]–[29].

Next, we investigated the ability of *TcBuster* to insert a drug-resistant transposon into the genome of HEK-293 cells. To optimize activity, we tested several doses of transposon and transposase plasmid. Colony number was linearly dependent on the amount of transposon plasmid that was transfected (**Fig. 2a**). Higher doses of transposase plasmid consistently increased the number of drug-resistant colonies to a point, after which the number decreased slightly, regardless of the amount of transposon plasmid in the cells (**Fig. 2**). This negative dosage effect mirrors that which was first observed and described by McClintock, who found that increasing *Ac* copy number from one to two to three copies caused a developmental delay in the timing of transposition [15]. Future experiments will investigate the extent of this effect, i.e. if higher transposase plasmid doses result in a complete loss of activity, as well as the mechanism behind this phenomenon. To compare transposition activity of *TcBuster* to *Tol2* and *piggyBac*, we performed colony assays and qPCR copy number analysis and determined that *TcBuster* is roughly equivalent by both of these measures to *piggyBac* and *Tol2*, both established transposon systems.

When genetically manipulating an organism, the possibility that the host genome itself may contain factors that could interact with the transposase or transposon being introduced is of interest and becomes a cause for concern when genomic instability is theoretically possible [34]. For example, if the transposase were introduced into a genome containing its transposon, it may induce hopping of the transposon and thus some degree of genomic destabilization. Another possibility is that the host genome expresses the transposase, but the inverted repeats are no longer present in the genome, and the introduction of the transposon causes genomic instability due to endogenous expression of the transposase. Although we hypothesized that the human *Buster* proteins were unable to induce transposition of the *TcBuster* element due to the lack of detectable transposon IR sequences flanking the human *Buster* proteins [18], we thought it prudent to test this experimentally. As shown in **Fig. 3**, there was no cross-transposition of the *TcBuster* transposon element by overexpressed human *Buster* proteins. The data shown here indicate that these proteins are likely to be incapable of mobilizing an integrated *TcBuster* transposon, an important consideration for the anticipated use of *TcBuster* in gene therapy applications.

We compared the integration preferences and activity of *TcBuster* to other transposons in HEK-293 cells, a male embryonic kidney-derived cell line. Simultaneously, the activity of *TcBuster* was assayed in HeLa cells, a cervical cancer cell line begun from a biopsy of tumor from an adult female [26]. In both cell types, *TcBuster* was highly active, had the same integration site motif, and preferred transcription start sites. Comparing the *TcBuster* integration sites in HEK-293 vs HeLa cells, 77.1% of the measurements showing the distance from integration sites to various genomic features were identical and non-identical results differed only slightly (a difference of 0.05 when comparing heat maps between the cell types). Therefore, many features of *TcBuster* are not cell type specific and we expect that *TcBuster* is likely to have a similar integration profile and to be highly active in most other human cell lines.

The integration site analysis of *TcBuster* revealed a preference and pattern slightly different than the other two most commonly used systems, *Sleeping Beauty* and *piggyBac*, and as has also been observed in HeLa cells [26]. Integration site differences between transposons have recently been harnessed for new cancer gene discovery in mice [13]. The consensus base pairs for each transposon's integration site indicated that each transposon had a requirement for a specific TA-rich sequence (**Fig. 4**). It naturally follows that a greater number of TA-rich integration sequences in a given region should increase the chances of the transposon finding a suitable integration site; therefore, the GC content of the integration regions was not enriched for any transposon over distances from the integration sites of 500 bp or less. In contrast to this requirement, our data also show that *piggyBac* and *TcBuster* preferred transcription start sites (**Fig. 5B**), which are known to be GC-rich. This preference may be due to epigenetic features of the transcription start sites rather than the DNA sequence of those sites. The transpososomes may be binding to proteins that are present at transcription start sites that enhance their affinity for the DNA, as is the preferred explanation for MLV transcription start site targeting [35], and/or the lack of nucleosomes may enhance integrations at transcription start sites, as is the case for the transposon *Hermes* in the yeast genome [36]. The integration site data was obtained from pools of selected clones, so only cells carrying at least one integration event that permitted sufficient levels of transgene expression were sequenced. Drug selection enriched for cells containing transposition events for all three groups of transposon insertions that were sequenced. However, *Sleeping Beauty* did not have a preference for transcription start sites, indicating that bias from drug selection did not cause increased insertion events near transcription start sites, as was observed for *TcBuster* and *piggyBac*. The data suggest that *TcBuster* represents a complementary tool for genomic engineering of animals for gene discovery and other applications that bias integration targeting to different parts of the genome.

*TcBuster* transposase has been codon-optimized for expression in mammalian cells. It is very likely that *TcBuster* activity can be improved further by screening for mutations to give hyperactive versions of the transposase, the identification of transposon IR sequences that are more easily transposed, the addition of sequences that may affect transposase protein dynamics and intracellular localization, and/or deletion of amino acids that inhibit transpososome formation. Future studies will test *TcBuster* in specific applications of transgenesis, insertional mutagenesis, and pre-clinical gene therapy in mammals to capitalize on the impressive performance of *TcBuster in vitro*.

## Materials and Methods

### Plasmid constructs

All plasmids were prepared for assays by endotoxin-free maxiprep (Qiagen, Valencia, CA). pUC19 (Invitrogen, Carlsbad, CA) was used as filler plasmid DNA for the FuGene6 transfections. The blasticidin resistance gene donor and transposase-expressing helper plasmids (pXL-TcB-D-GFP/Bsd and pXL-CMV-TcBuster_CO_ for *TcBuster*, pXL-PB-D-GFP/Bsd and pXL-CMV-piggyBac for *piggyBac*, or pXL-SB-D-GFP/Bsd and pXL-CMV-Sleeping Beauty for *Sleeping Beauty*) used for excision PCR and integration site analysis are described elsewhere [26]. Briefly, the donor plasmids are based on pCMV/Zeo (Invitrogen) in which the Zeo gene was replaced with a GFP/Bsd fusion protein from pTracer/CMV-Bsd (Invitrogen) and flanked by the transposon IRs. The helper plasmids are based on pcDNA3.1/myc-HisA (Invitrogen) and contain the CMV promoter controlling transposase coding sequence.

Construction of the plasmids pCMV-*piggyBac* [7], pCMV-HA-*piggyBac* [33], and pTpB [7] has been reported elsewhere. pCMV-*TcBuster* was constructed by polymerase chain reaction (PCR) amplification of the codon-optimized *TcBuster* transposase open reading frame from the plasmid pXL-CMV-TcBuster_CO_. The PCR product was cloned into the backbone vector of pCMV-HA-*piggyBac*, consisting of the cytomegalovirus (CMV) promoter, a unique SacII site, the hemagglutinin (HA) tag, the *piggyBac* open reading frame, a unique KpnI site, and the polyA tail. The forward primer SacIITCB2 (GCTGCCGCGGATGATGCTGAATTGGCTGAAAAGC) was used to add a SacII site to the N-terminus to create pCMV-*TcBuster*. The reverse primer was RevKpnITCB2 (GCCGGGTACCTCAGTGAGATTTCTGGGCCTGC) to add a KpnI site to the C-terminus. The enzyme PfuUltra (Agilent-Strategene, Santa Clara, CA) was used for PCR at 95°C for 2 min; 35 cycles of 95°C for 30 sec, 55°C for 30 sec, and 72°C for 1.5 min; and 72°C for 7 min. The PCR product was digested, gel-purified by freeze and squeeze (BioRad, Hercules, CA), and cleaned and concentrated (ZYMO Research, Irvine, CA) to generate the insert. To make the vector backbone fragments, the plasmid pCMV-HA-*piggyBac* was digested with SacII and KpnI to liberate the HA-*piggyBac* cassette. After digest, reactions were cleaned using the PCR purification kit (Qiagen), dephosphorylated with Antarctic Phosphatase (New England Biolabs, Ipswich, MA), heat-killed, and gel-purified (Qiagen). Ligation was performed using Quick Ligase (New England Biolabs) for five min at room temperature, purified by clean and concentrate kit (ZYMO Research), and half of the reaction was transformed into electrocompetent *E. coli* strain DH10β (Invitrogen) and selected on ampicillin agar plates (Invitrogen). Similar molecular biology protocols were used to generate all other constructs. pCMV-*TcBuster* was confirmed by sequencing (Genewiz, South Plainfield, NJ and Lone Star Labs, Houston, TX).

pTcBNeo was created by cloning the neomycin cassette from pTpB into the plasmid pXL-TcB-D-GFP/Bsd [26]. pTpB was digested with EcoRI and BamHI to generate the neomycin transposon insert. This was cloned into pXL-TcB-D-GFP/Bsd that was digested with EcoRI and BglII to give just the backbone vector containing the *TcBuster* inverted repeats (IRs). The resultant pTcBNeo clone was fully sequenced. The pCMV-*Tol2* and pMiniTol2/MCS plasmids were generously provided by Stephen Ekker [6]. pTcBNeo was digested with ApoI to isolate the Neo cassette and PvuI to cut the backbone into smaller fragments. pMiniTol2/MCS was digested with EcoRI and dephosphorylated to generate the backbone containing the *Tol2* IRs. After ligation and selection, the resultant constructs were sequenced to confirm the desired structure of pTol2Neo.

To express the human Buster proteins, plasmids based on the vector pCMV-Sport6 containing the human MGC-verified cDNA for each gene (OpenBiosystems, Lafayette, CO: Buster 1, clone ID 6471771; Buster 3, clone ID 5535434; and SCAND3, clone ID 6499684) were confirmed by sequencing (Lone Star Labs). The standard midiprep procedure (Qiagen) was modified according to manufacturer's protocols to produce endotoxin-free DNA for transfection.

### Tissue culture

HEK-293 cells (ATCC, Manassas, VA) were maintained in cellgro Minimum Essential Medium, Alpha 1× with Earle's Salts, ribonucleosides, deoxyribonucleosides, and L-glutamine (Mediatech, Inc., Manassas, VA) that was supplemented with 10% fetal bovine serum (HyClone/Thermo Scientific, Logan, UT) and 1% penicillin/streptomycin (Gibco/Invitrogen) and filter sterilized.

### Excision PCR assay

HEK-293 cells in 6-well dishes were transfected by FuGene6 (Roche). After 48 hrs, the cells were trypsinized, pelleted, washed with PBS, and resuspended in 600 µl of PBS. Minipreps were done on the samples according to the manufacturer's directions (Zymo Research), except that the neutralization buffer addition was followed by a 5 min incubation period. Polymerase chain reaction (PCR) was performed in two rounds, both with 1 µl of DNA template and 0.2 M of each primer in 1× Taq-Pro Red COMPLETE 2.5 mM MgCl_2_ (Denville Scientific, Metuchen, NJ) at 94°C for 5 min, followed by 60 cycles of 94°C for 30 sec, 60°C for 30 sec, 72°C for 20 sec, and a final extension at 72°C for 10 min. The second round PCR was the same except that the cycle number was reduced to 35 cycles. The first round primers were TcBx1F (CGAACGACCTACACCGAACT) and TcBx1R (GGTGATGACGGTGAAAACCT); the second round primers were TcBx2F (CAGCGTGAGCATTGAGAAAG) and TcBx2R (CGGCATCAGAGCAGATTGTA). The first-round and second-round bands were excised from an agarose gel, purified, TOPO cloned (Invitrogen), and sequenced. Alignment and chromatogram analysis were performed with CloneManager software (Scientific and Educational Software, Cary, NC).

### Colony counts

HEK-293 cells seeded onto 6-well plates were transfected the next day in triplicate according to the manufacturer's instructions with FuGene6 (Roche) at a 3∶1 ratio of FuGene6 to DNA. The cells were trypsinized 48 hours later, reconstituted in 1 ml of media, and 13.33 µl was transferred to dilute the cells by 1∶750 onto 10 cm plates containing 10 ml of media supplemented with 1 mg/ml geneticin (Invitrogen). The media was changed one week later. After two weeks, the cells were simultaneously fixed and stained with a solution of 50% methanol (VWR, Radnor, PA) and 1% methylene blue (Invitrogen). After 30–60 minutes incubation, the plates were washed twice with phosphate-buffered saline (PBS), allowed to air dry, and all visible colonies were counted.

### Analysis of transposon integration sites in HEK-293 cells

HEK-293 cells were seeded at 2×10^6^ in 10-cm plates and were transfected with FuGENE 6 (Roche) with 2.0 µg of helper plasmid and 8.0 µg of donor plasmid. Forty-eight hours after transfection, cells were trypsinized and diluted onto 6–8 10 cm plates and cultured in DMEM medium with 5% FBS containing 3.5 µg/ml blasticidin. Drug selection was continued for 18–21 days, changing the media every 2–3 days. Surviving cells were harvested and the genomic DNA was purified using the DNeasy Blood and Tissue Kit (Qiagen). Integration sites were recovered as described [37]. Briefly, 2 µg of genomic DNA was digested overnight with ApoI or BstYI and ligated to linkers overnight at 16°C. Nested PCR was then carried out under stringent conditions using end-specific primers complementary to transposon sequences and linker-specific primers complementary to the DNA linker. Primers used in this study have been previously described [26]. DNA barcodes were included in the second-round PCR primers in order to track sample origin. The PCR products were gel purified, pooled, and sequenced using the 454 sequencing platform (Roche). Only sequences that uniquely aligned to the human genome by BLAT (hg18, version 36.1, >98% match score) and began within 3 bp of the terminal repeat end were used in downstream analyses. A 20-bp target DNA sequence surrounding each integration site was extracted from the draft human genome (hg18), and aligned using WebLogo (http://weblogo.Berkeley.edu/logo.cgi). Detailed bioinformatic methods for analysis of association with chromosomal features are described in Berry et al [32]. The methods for generating heat maps based in receiver operating characteristic curves are as described in Berry et al [32]. The integration site datasets are available from NCBI under accessions JS717545–JS799249.

### qPCR assay for transposon copies per cell

The primers and experimental conditions for the qPCR assays for *Neo* and *RNaseP* have been described previously [8]. Briefly, HEK-293 cells in 6-well plates were transfected by FuGene 6 in triplicate with 500 ng of transposon, 100 ng of transposase and 400 ng of pUC19 per well. Cells were diluted and plated into drug-selection media 2 days following transfection. After approximately 3 weeks the plates containing colonies were trypsinized, replated and allowed to grow to confluence in 10 cm dishes. Genomic DNA was harvested by DNeasy Blood and Tissue Kit (Qiagen) and digested with DpnI (New England Biolabs) to remove residual bacterially-methylated plasmid DNA. There are three restriction sites for DpnI in the *Neo* gene product; therefore this method effectively prevents plasmid DNA from being amplified. The enzyme reactions were heat-killed at 80°C for 30 minutes.The qPCR standard curves were either serial dilutions of pTpB in non-transfected HEK-293 genomic DNA to determine the copy number for the neomycin resistance gene or serial dilutions of a plasmid containing a fragment of the *RNaseP* gene (pRNaseP) to determine the *RNaseP* copy number. *RNaseP* is commonly used to determine the amount of genomic DNA present in the qPCR reaction because it is known to have only two copies per diploid genome. Previously reported primer sets for *Neo* and *RNaseP* (Sigma) amplified the gene products on a CFX96/C1000 real-time system (BioRad) from 20 ng of DpnI-treated genomic DNA in a 25 µl reaction of 1× iQ SYBR Green Master Mix (BioRad) for 40 cycles at an annealing temperature of 60°C. Standard curves were generated and lines of best fit were calculated using CFX Manager (BioRad) and Excel (Microsoft, Seattle, WA). Melting curves suggested PCR amplification of a single product in all reactions other than the negative controls (water and naïve HEK-293 genomic DNA only).

## Supporting Information

Figure S1 Integration sites of *piggyBac*, *Sleeping Beauty*, and *TcBuster* in relation to repeat elements. Integration sites rescued from HEK-293 cells were analyzed for integration into and near genomic repeats. (**a**) Integration into genomic repeats for *piggyBac* (red), *Sleeping Beauty* (green), and *TcBuster* (blue). The graph shows the percentage of integrations (y-axis) that occurred within each genomic repeat element (x-axis). (**b**) The proximity of transposon integration sites to the repeat elements. Each mini-graph displays the percentage of sites (y-axis) that were within windows of <1 kb, <5 kb, <10 kb, <50 kb, <100 kb, or <500 kb (x-axis) from each repeat element (title of mini-graph). kb = kilobase pairs
(TIF)

Figure S2 Full report on the association of genomic features with integration sites of the *Sleeping Beauty*, *piggyBac*, and *TcBuster* transposons.
(PDF)
