# Supplementary figures and images for: Comparative Analysis of the Recently Discovered hAT Transposon TcBuster in Human Cells
Source: PLoS One. 2012 Nov 15;7(11):e42666. doi: 10.1371/journal.pone.0042666 (PMC3499496; doi:10.1371/journal.pone.0042666)

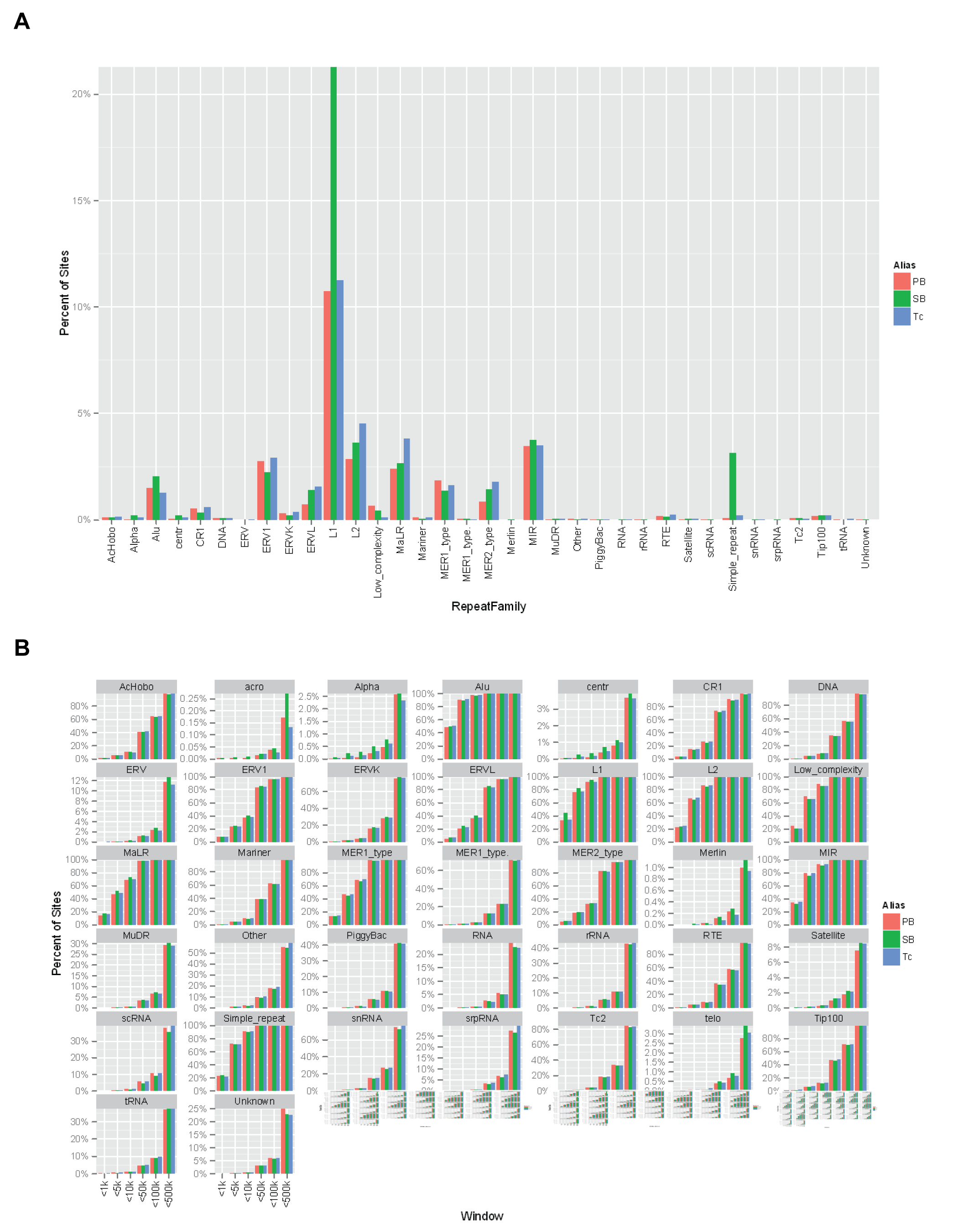

Supplement: Figure S1 — Integration sites of piggyBac, Sleeping Beauty, and TcBuster in relation to repeat elements. Integration sites rescued from HEK-293 cells were analyzed for integration into and near genomic repeats. (a) Integration into genomic repeats for piggyBac (red), Sleeping Beauty (green), and TcBuster (blue). The graph shows the percentage of integrations (y-axis) that occurred within each genomic repeat element (x-axis). (b) The proximity of transposon integration sites to the repeat elements. Each mini-graph displays the percentage of sites (y-axis) that were within windows of <1 kb, <5 kb, <10 kb, <50 kb, <100 kb, or <500 kb (x-axis) from each repeat element (title of mini-graph). kb = kilobase pairs (TIF) [file pone.0042666.s001.tif]
