# Supplementary material for: Comparative Analysis of the Recently Discovered hAT Transposon TcBuster in Human Cells
Source: PLoS One. 2012 Nov 15;7(11):e42666. doi: 10.1371/journal.pone.0042666 (PMC3499496; doi:10.1371/journal.pone.0042666)
Supplement: Figure S2 — Full report on the association of genomic features with integration sites of the Sleeping Beauty, piggyBac, and TcBuster transposons. (PDF) [file pone.0042666.s002.pdf]

Association of Genomic Features with Integration

March 28, 2012

Contents

1 Introduction 2

2 Preference for Genes 4
2.1 Acembly Genes 4
2.2 refGenes 6
2.3 ensGenes 7
2.4 genScan Genes 9
2.5 uniGenes 11
2.6 oncogenes 14

3 CpG Island Neighborhoods 15
3.1 1 kilobase neighborhoods 15
3.2 2 kilobase neighborhoods 16
3.3 5 kilobase neighborhoods 17
3.4 10 kilobase neighborhoods 17
3.5 25 kilobase neighborhoods 18
3.6 50 kilobase neighborhoods 19

4 Gene Density, Expression 'Density', and CpG Island Density 21
4.1 25 kilobase Window 21
4.2 50 kilobase Window 25
4.3 100 kilobase Window 30
4.4 250 kilobase Window 35
4.5 500 kilobase Window 40
4.6 1 megabase Window 45
4.7 2 megabase Window 50
4.8 4 megabase Window 55
4.9 8 megabase Window 60
4.10 16 megabase Window 65
4.11 32 megabase Window 70

5 Juxtaposition with Gene Start and End Positions **76**
5.1 Acembly Annotations 76
5.2 RefSeq Annotations 80
5.3 genScan Annotations 84
5.4 uniGene Annotations 88

6 GC content **92**

1 Introduction

In this document, I examine the association of integration sites with various genomic features.

The data consist of both actual integration sites and sets of control sites, each set chosen to match the spacing (in bases) from the nearest restriction site (according to the direction in which the sequence was read) to an integration site. The numbers of insertion and matching sites for several data sets are shown below:

```
                  type
Origin.of.data.set insertion match
             a)SB      30303 90887
             b)PB      23417 70250
             c)TC      27985 83954
```

The advantage of choosing 'control' sites that match the spacing from the nearest restriction site is that biases due to location and density of restriction sites are eliminated by applying the classical multinomial logit model (reviewed in [2]). This model allows regression procedures to be applied to the study of integration intensity as a function of genomic features. The `clogit` function of the R `survival` library) implements estimation and fitting for such models along with the usual likelihood ratio and Wald tests.

The distribution of relative frequency of insertions across the chromosomes is given in this barplot:

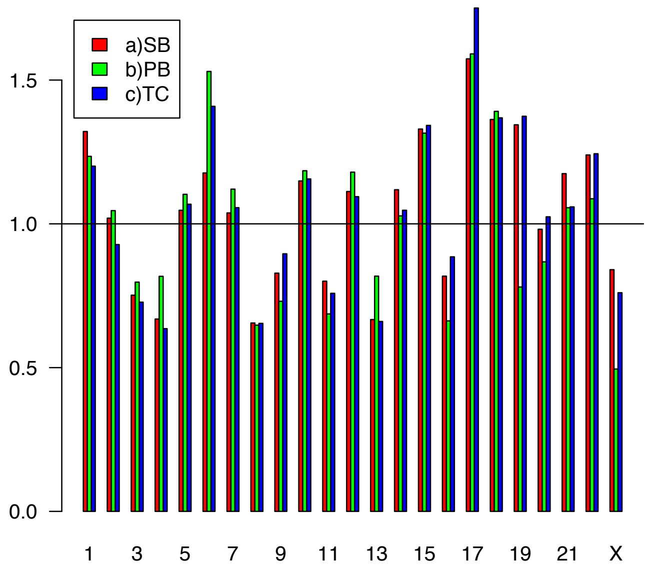

It seems evident that there are some chromosomes that are particularly favored for integration. This is reinforced by a test of statistical significance. The test performed used the likelihood ratio statistic for the multinomial logit model (reviewed in [2]) as implemented by the `clogit` function of the R `survival` library). The null hypothesis tested is that the ratio of true integration events to matched control sites is constant across all chromosomes. This test attains a p-value of $< 2.22e - 16$.

2 Preference for Genes

2.1 Acembly Genes

Here we examine the preference that integration events have for genes. In the following plot we show the relative frequency of integrations in genes according to the 'Acembly' annotation. The bars grouped over the label "In Gene" give the relative frequency of integration events (compared to control sites) between bases located within Acembly gene annotations, while the label "Not in Gene" give the relative frequency of integration events (compared to control sites) between bases not located within Acembly gene annotations.

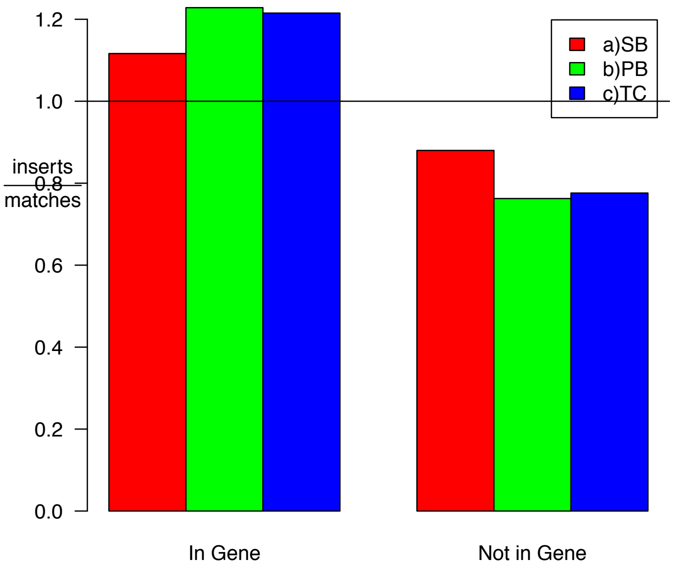

It seems evident that there is a strong tendency for insertions to occur in genes. A formal test of significance bears this out with a p-value of $< 2.22e-16$. Also, it appears that the tendency of different viruses to integrate into genes varies, and a test for this hypothesis attains $< 2.22e - 16$. Here is the table of coefficients of the log ratio of intensities for true insertion sites versus control insertion sites along with their standard errors, z statistics, and p-values for each data set:

```
      coef     se    z         p
a)SB 0.238 0.0134 17.8  5.80e-71
b)PB 0.478 0.0155 30.7 1.59e-207
c)TC 0.449 0.0142 31.7 2.49e-220
```

As is evident, there are some differences in the coefficients. The largest coefficient is seen in the b)PB data set, while the smallest is seen in the a)SB data set.

In the following plot we show the relative frequency of insertions in exons according to the 'Acembly' annotation. The bars grouped over the label "In Exon" give the relative frequency of integration events (compared to control sites) between bases located in exons according to the Acembly annotation, while the label "Not in Exon" give the relative frequency of integration events (compared to control sites) between bases not located in exons according to the Acembly gene annotation.

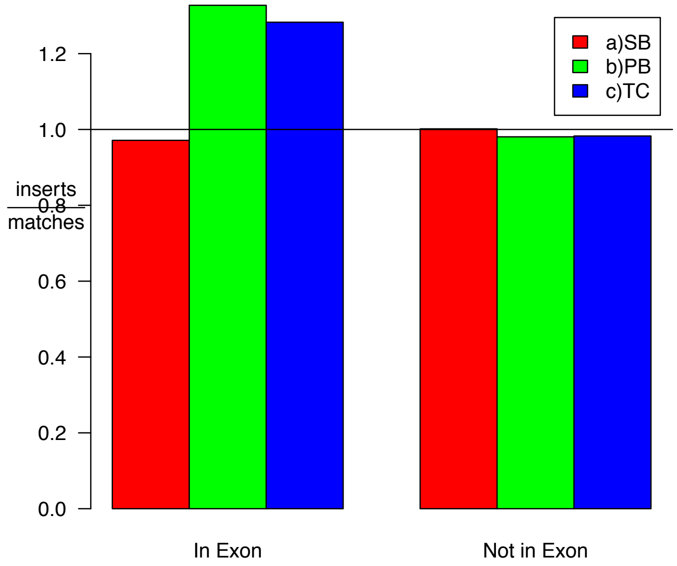

Here is the table of coefficients of the log ratio of intensities for true insertion sites versus control insertion sites along with their standard errors, z statistics, and p-values for each data set:

	coef	se	z	p
a)SB	-0.1540	0.0302	-5.09	3.60e-07
b)PB	0.0850	0.0307	2.77	5.63e-03
c)TC	0.0618	0.0280	2.21	2.74e-02

The model on which these coefficients are based include terms for whether the site is in a gene or not. Thus, the effect shown as "In Exon" is net of that due to being in a gene. Note that in the barplot above the 'Not in Exon' bars include both the introns and intergenic regions, so the impression given by the

table may differ from that for the barplot.

2.2 refGenes

Here we examine the preference that insertions have for genes. In the following plot we show the relative frequency of insertions in genes according to the 'refGene' annotation.

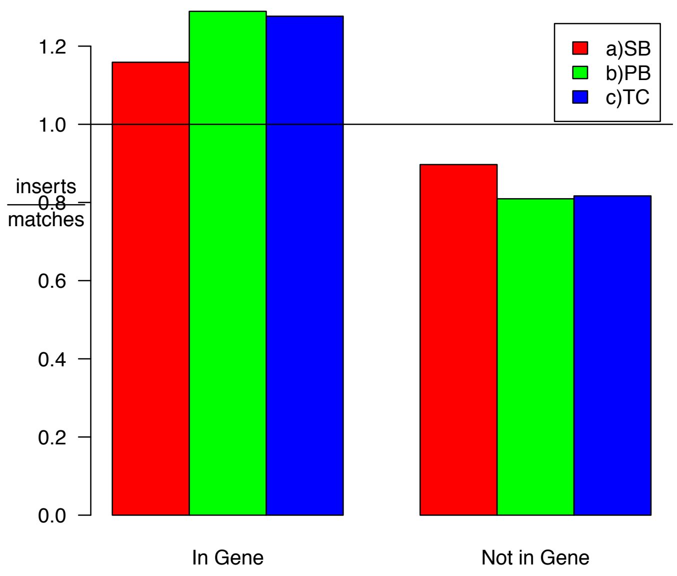

It seems evident that there is a strong tendency for insertions to occur in genes. A formal test of significance bears this out with a p-value of $< 2.22e-16$. Also, it appears that the tendency of different viruses to integrate into genes varies, and a test for this hypothesis attains $< 2.22e-16$. Here is the table of coefficients of the log ratio of intensities for true insertion sites versus control insertion sites along with their standard errors, z statistics, and p-values for each data set:

	coef	se	z	p
a)SB	0.255	0.0134	19.1	3.29e-81
b)PB	0.465	0.0152	30.5	7.10e-205
c)TC	0.447	0.0139	32.1	8.25e-226

As is evident, there are some differences in the coefficients. The largest coefficient is seen in the b)PB data set, while the smallest is seen in the a)SB data set.

In the following plot we show the relative frequency of insertions in exons according to the 'refGene' annotation.

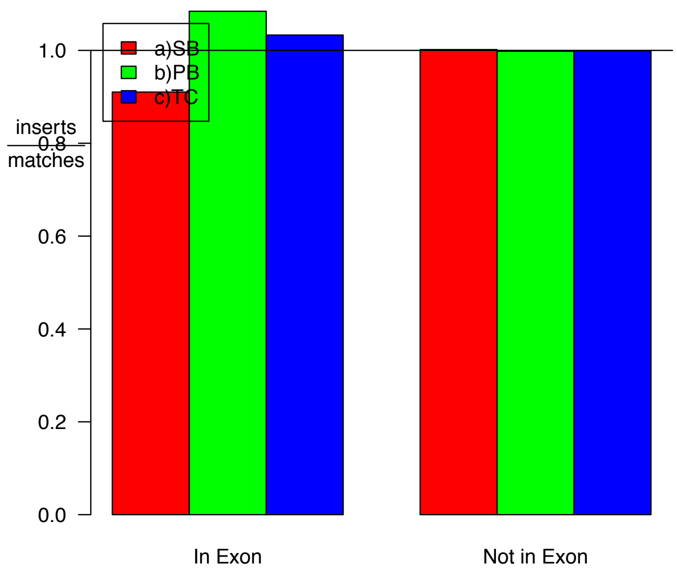

Here is the table of coefficients of the log ratio of intensities for true insertion sites versus control insertion sites along with their standard errors, z statistics, and p-values for each data set:

	coef	se	z	p
a)SB	-0.248	0.0501	-4.95	7.48e-07
b)PB	-0.183	0.0530	-3.45	5.62e-04
c)TC	-0.224	0.0473	-4.73	2.30e-06

The model on which these coefficients are based include terms for whether the site is in a gene or not. Thus, the effect shown as "In Exon" is net of that due to being in a gene.

2.3 ensGenes

Here we examine the preference that insertions have for genes. In the following plot we show the relative frequency of insertions in genes according to the 'ensGene' annotation.

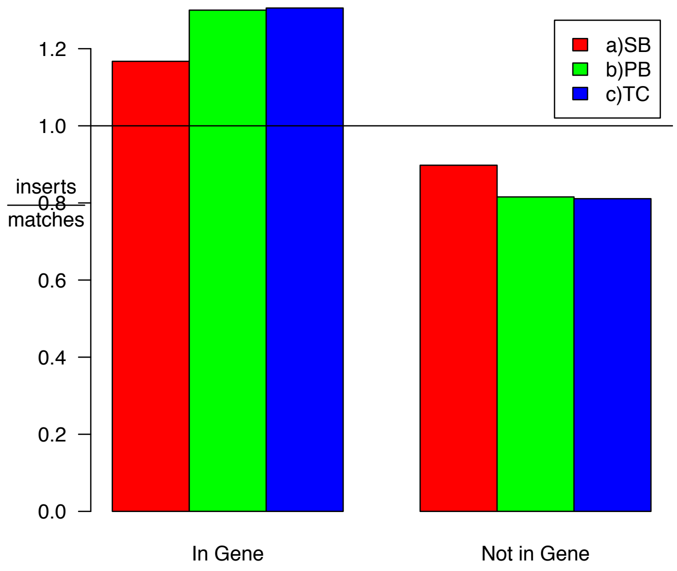

It seems evident that there is a strong tendency for insertions to occur in genes. A formal test of significance bears this out with a p-value of $< 2.22e-16$. Also, it appears that the tendency of different viruses to integrate into genes varies, and a test for this hypothesis attains $< 2.22e-16$. Here is the table of coefficients of the log ratio of intensities for true insertion sites versus control insertion sites along with their standard errors, z statistics, and p-values for each data set:

```
      coef     se    z          p
a)SB 0.263 0.0135 19.5   7.30e-85
b)PB 0.467 0.0153 30.6 2.47e-205
c)TC 0.478 0.0140 34.2 1.08e-255
```

As is evident, there are some differences in the coefficients. The largest coefficient is seen in the c)TC data set, while the smallest is seen in the a)SB data set.

In the following plot we show the relative frequency of insertions in exons according to the 'ensGene' annotation.

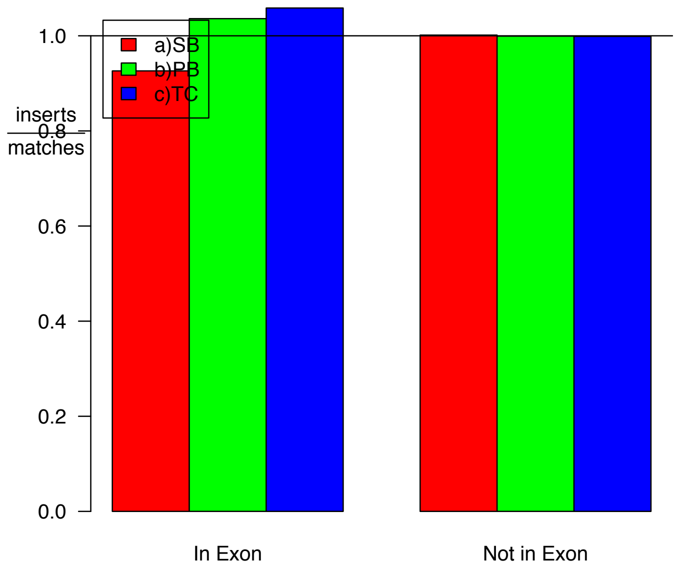

Here is the table of coefficients of the log ratio of intensities for true insertion sites versus control insertion sites along with their standard errors, z statistics, and p-values for each data set:

```
       coef     se     z        p
a)SB -0.239 0.0499 -4.78 1.76e-06
b)PB -0.237 0.0539 -4.40 1.10e-05
c)TC -0.221 0.0473 -4.67 3.08e-06
```

The model on which these coefficients are based include terms for whether the site is in a gene or not. Thus, the effect shown as "In Exon" is net of that due to being in a gene.

2.4 genScan Genes

Here we examine the preference that insertions have for genes. In the following plot we show the relative frequency of insertions in genes according to the 'genScan' annotation.

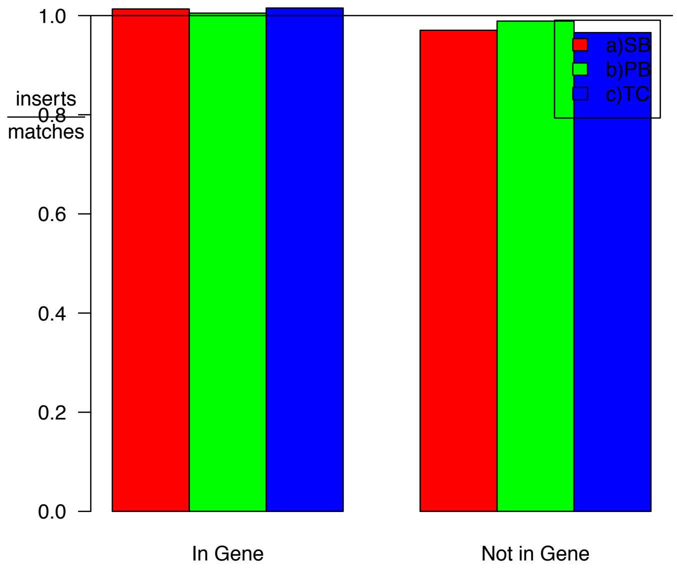

It seems evident that there is a strong tendency for insertions to occur in genes. A formal test of significance bears this out with a p-value of $1.6525e-05$. Also, it appears that the tendency of different viruses to integrate into genes varies, and a test for this hypothesis attains 0.2766. Here is the table of coefficients of the log ratio of intensities for true insertion sites versus control insertion sites along with their standard errors, z statistics, and p-values for each data set:

	coef	se	z	p
a)SB	0.0433	0.0144	3.000	0.00266
b)PB	0.0161	0.0164	0.984	0.32500
c)TC	0.0502	0.0151	3.330	0.00088

As is evident, there are some differences in the coefficients. The largest coefficient is seen in the c)TC data set, while the smallest is seen in the b)PB data set.

In the following plot we show the relative frequency of insertions in exons according to the 'genScan' annotation.

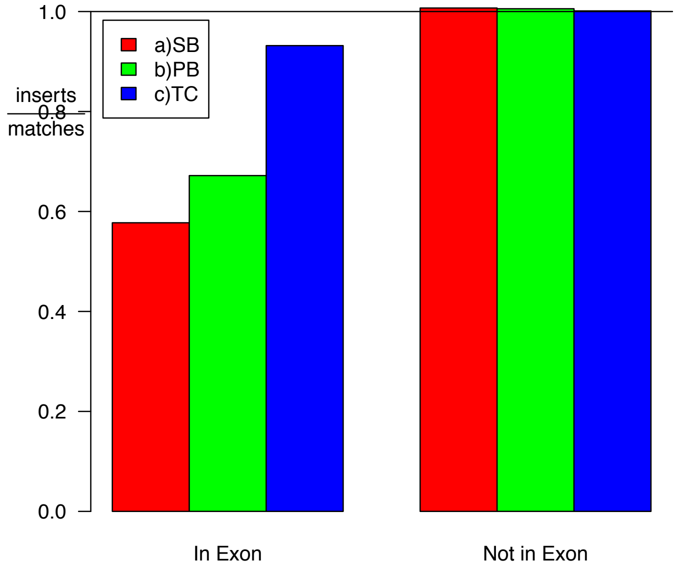

Here is the table of coefficients of the log ratio of intensities for true insertion sites versus control insertion sites along with their standard errors, z statistics, and p-values for each data set:

```
        coef     se     z        p
a)SB -0.5730 0.0651 -8.80 1.35e-18
b)PB -0.4100 0.0689 -5.96 2.58e-09
c)TC -0.0881 0.0541 -1.63 1.03e-01
```

The model on which these coefficients are based include terms for whether the site is in a gene or not. Thus, the effect shown as "In Exon" is net of that due to being in a gene.

2.5 uniGenes

Here we examine the preference that insertions have for genes. In the following plot we show the relative frequency of insertions in genes according to the 'uniGene' annotation.

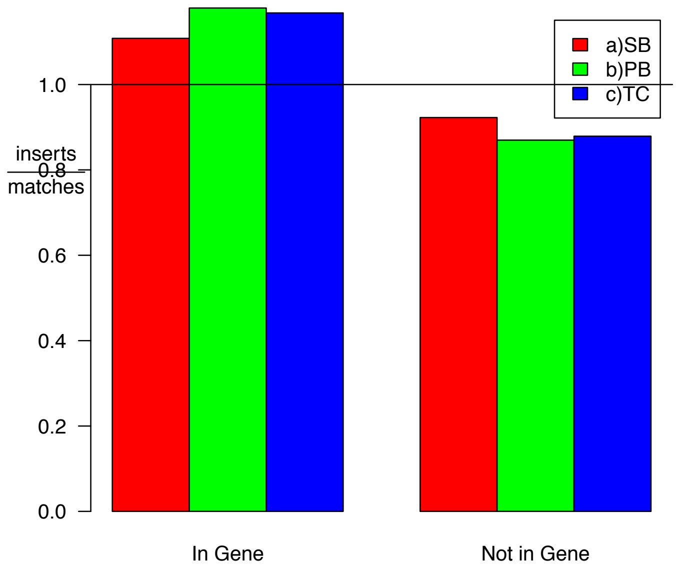

It seems evident that there is a strong tendency for insertions to occur in genes. A formal test of significance bears this out with a p-value of $< 2.22e-16$. Also, it appears that the tendency of different viruses to integrate into genes varies, and a test for this hypothesis attains $3.1551e-10$. Here is the table of coefficients of the log ratio of intensities for true insertion sites versus control insertion sites along with their standard errors, z statistics, and p-values for each data set:

```
      coef     se    z        p
a)SB 0.184 0.0134 13.7 5.97e-43
b)PB 0.305 0.0152 20.1 7.43e-90
c)TC 0.284 0.0139 20.5 3.17e-93
```

As is evident, there are some differences in the coefficients. The largest coefficient is seen in the b)PB data set, while the smallest is seen in the a)SB data set.

In the following plot we show the relative frequency of insertions in exons according to the 'uniGene' annotation.

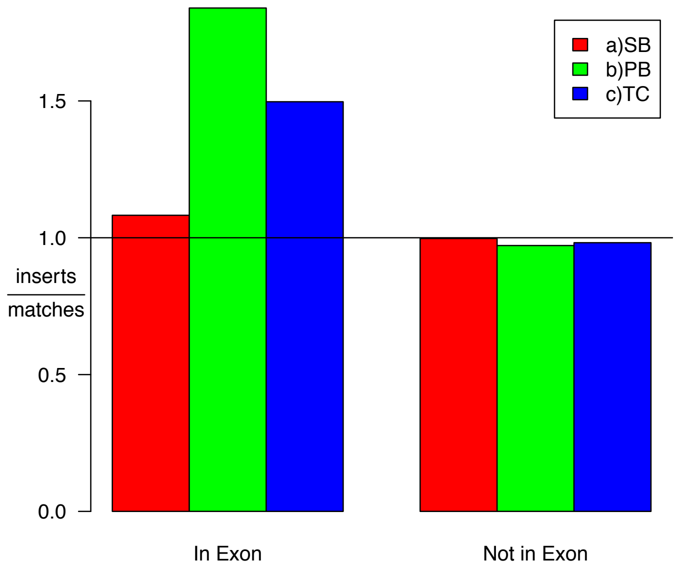

Here is the table of coefficients of the log ratio of intensities for true insertion sites versus control insertion sites along with their standard errors, z statistics, and p-values for each data set:

	coef	se	z	p
a)SB	-0.025	0.0372	-0.67	5.03e-01
b)PB	0.496	0.0358	13.90	9.84e-44
c)TC	0.278	0.0336	8.27	1.31e-16

The model on which these coefficients are based include terms for whether the site is in a gene or not. Thus, the effect shown as ”In Exon” is net of that due to being in a gene.

2.6 oncogenes

Here we examine the preference that insertions have for oncogenes. In the following plot we show the relative frequency of insertions with 50kb of an oncogene 5' end.

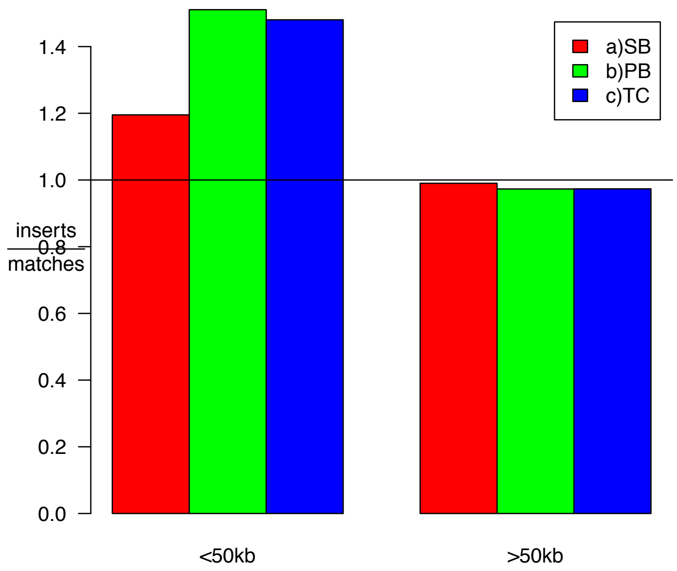

A formal test of oncogenic insertion returns p-value of $< 2.22e-16$. The tendency of different viruses to integrate near oncogenes may vary, and a test for this hypothesis attains $6.8314e-11$. Here is the table of coefficients of the log ratio of intensities for true insertion sites versus control insertion sites along with their standard errors, z statistics, and p-values for each data set:

	coef	se	z	p
a)SB	-0.188	0.0291	-6.46	1.04e-10
b)PB	-0.441	0.0302	-14.60	2.63e-48
c)TC	-0.419	0.0272	-15.40	1.46e-53
a)SB	NA	0.0000	NA	NA
b)PB	NA	0.0000	NA	NA
c)TC	NA	0.0000	NA	NA

As is evident, there are some differences in the coefficients. The largest coefficient is seen in the a)SB data set, while the smallest is seen in the b)PB data set.

3 CpG Island Neighborhoods

Here we study the effect of being in the neighborhood of CpG Islands. Following Wu et al [3], who found that the neighborhoods within ±1kb of CpG islands are enriched for MLV insertions, we study such neighborhoods.

3.1 1 kilobase neighborhoods

The following plot shows the effect of being in or within ±1kb of a CpG island:

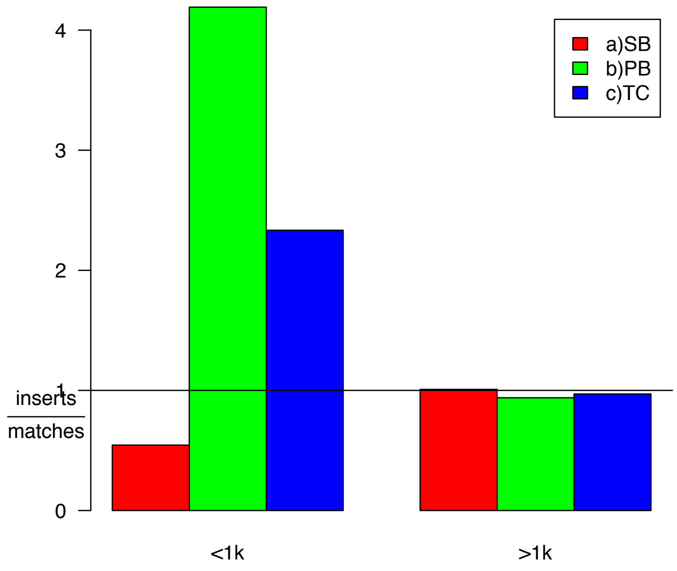

A formal test of significance comparing the difference attains a p-value of $< 2.22e - 16$. A test for differences between viruses attains $< 2.22e - 16$. Here is the table of coefficients of the log ratio of intensities for true insertion sites versus control insertion sites along with their standard errors, z statistics, and p-values for each data set:

	coef	se	z	p
a)SB	-0.618	0.0627	-9.85	6.54e-23
b)PB	1.500	0.0374	40.20	0.00e+00
c)TC	0.881	0.0362	24.30	7.92e-131

The largest coefficient is seen in the b)PB data set, while the smallest is seen in the a)SB data set.

3.2 2 kilobase neighborhoods

The following plot shows the effect of being in or within ±2kb of a CpG island:

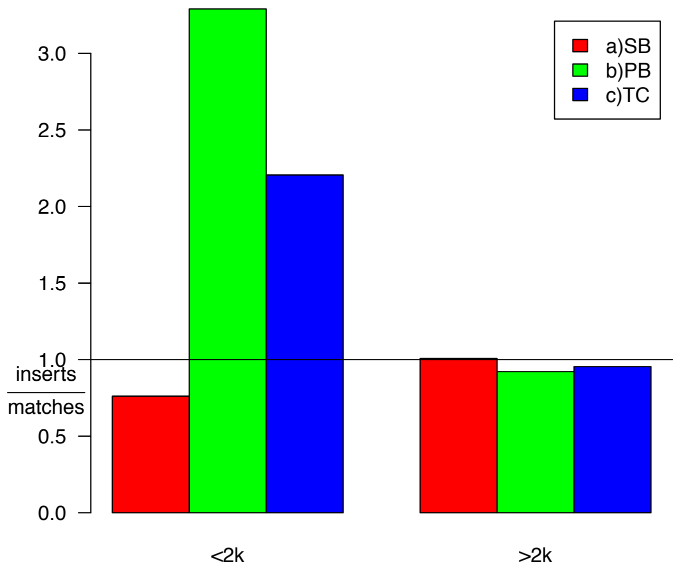

A formal test of significance comparing the difference attains a p-value of $< 2.22e - 16$. A test for differences between viruses attains $< 2.22e - 16$. Here is the table of coefficients of the log ratio of intensities for true insertion sites versus control insertion sites along with their standard errors, z statistics, and p-values for each data set:

	coef	se	z	p
a)SB	-0.281	0.0410	-6.85	7.47e-12
b)PB	1.280	0.0301	42.40	0.00e+00
c)TC	0.840	0.0287	29.30	3.00e-188

The largest coefficient is seen in the b)PB data set, while the smallest is seen in the a)SB data set.

3.3 5 kilobase neighborhoods

The following plot shows the effect of being in or within ±5kb of a CpG island:

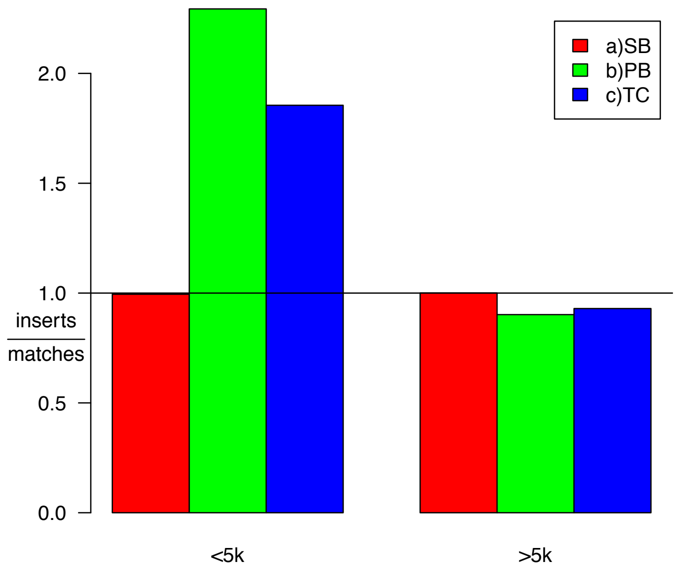

A formal test of significance comparing the difference attains a p-value of $< 2.22e - 16$. A test for differences between viruses attains $< 2.22e - 16$. Here is the table of coefficients of the log ratio of intensities for true insertion sites versus control insertion sites along with their standard errors, z statistics, and p-values for each data set:

	coef	se	z	p
a)SB	-0.00575	0.0260	-0.221	8.25e-01
b)PB	0.93400	0.0233	40.100	0.00e+00
c)TC	0.69300	0.0216	32.000	9.31e-225

The largest coefficient is seen in the b)PB data set, while the smallest is seen in the a)SB data set.

3.4 10 kilobase neighborhoods

The following plot shows the effect of being in or within ±10kb of a CpG island:

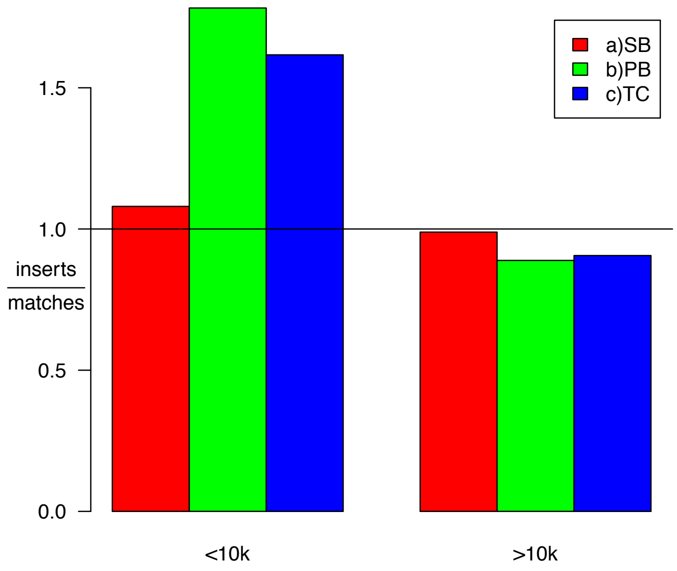

A formal test of significance comparing the difference attains a p-value of $< 2.22e - 16$. A test for differences between viruses attains $< 2.22e - 16$. Here is the table of coefficients of the log ratio of intensities for true insertion sites versus control insertion sites along with their standard errors, z statistics, and p-values for each data set:

	coef	se	z	p
a)SB	0.0883	0.0198	4.46	8.10e-06
b)PB	0.7000	0.0196	35.70	6.63e-279
c)TC	0.5820	0.0179	32.50	4.61e-232

The largest coefficient is seen in the b)PB data set, while the smallest is seen in the a)SB data set.

3.5 25 kilobase neighborhoods

The following plot shows the effect of being in or within ±25kb of a CpG island:

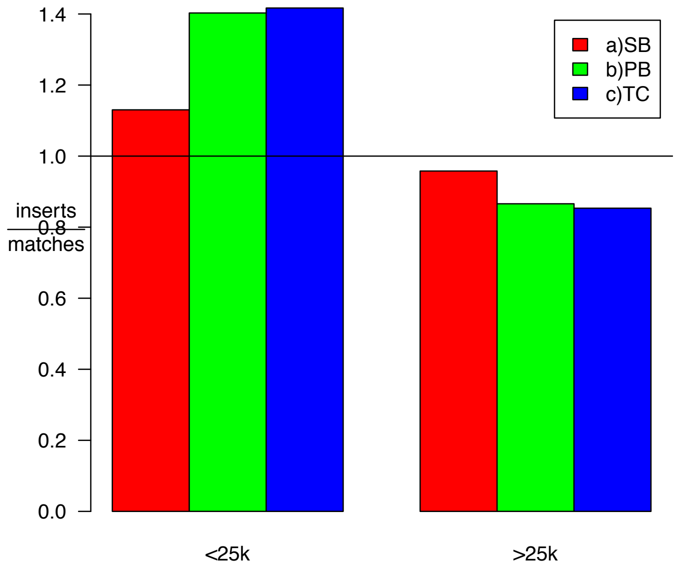

A formal test of significance comparing the difference attains a p-value of $< 2.22e-16$. A test for differences between viruses attains $< 2.22e-16$. Here is the table of coefficients of the log ratio of intensities for true insertion sites versus control insertion sites along with their standard errors, z statistics, and p-values for each data set:

	coef	se	z	p
a)SB	0.166	0.0150	11.1	2.16e-28
b)PB	0.484	0.0163	29.7	2.82e-193
c)TC	0.508	0.0148	34.5	3.69e-260

The largest coefficient is seen in the c)TC data set, while the smallest is seen in the a)SB data set.

3.6 50 kilobase neighborhoods

The following plot shows the effect of being in or within ±50kb of a CpG island:

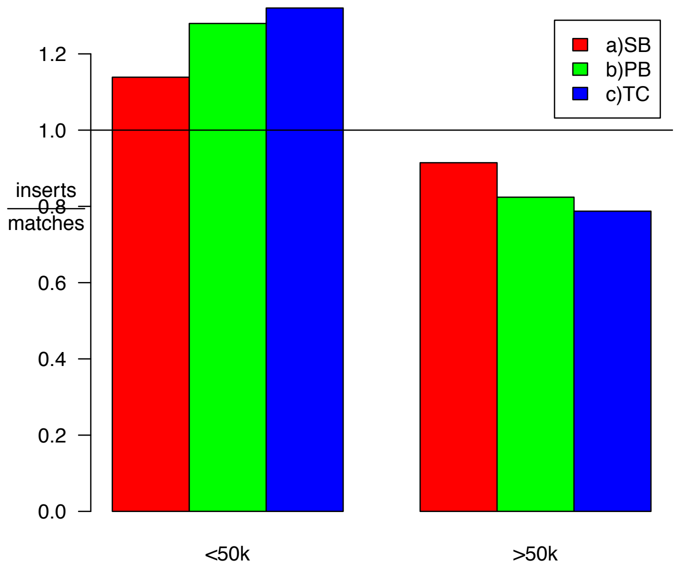

A formal test of significance comparing the difference attains a p-value of $< 2.22e - 16$. A test for differences between viruses attains $< 2.22e - 16$. Here is the table of coefficients of the log ratio of intensities for true insertion sites versus control insertion sites along with their standard errors, z statistics, and p-values for each data set:

	coef	se	z	p
a)SB	0.220	0.0135	16.3	7.96e-60
b)PB	0.440	0.0152	28.9	2.43e-183
c)TC	0.516	0.0140	37.0	5.91e-300

The largest coefficient is seen in the c)TC data set, while the smallest is seen in the a)SB data set.

4 Gene Density, Expression 'Density', and CpG Island Density

In this section the association with gene density is examined. For expression analysis, the 'genes' that are counted are the genes represented on the microarray. In addition, we the number of such genes expressed at various levels. The levels are

low.ex Count genes whose expression is in the upper half and divide by number of bases

med.ex Count genes whose expression is in the upper $1/8^{th}$ and divide by number of bases

high.ex Count genes whose expression is in the upper $1/16^{th}$ and divide by number of bases

The bolded terms are used as abbreviations in what follows. The abbreviation **dens** is used to indicate gene density as number of genes per base.

4.1 25 kilobase Window

In the barplot that follows we examine the association of insertion sites with gene density in a 25 kilobase window surrounding each locus. More such plots will follow and the method of their construction is always to try to divide the data according to the deciles of density. However, it often happens that there is a very skewed distribution of density and even the 90th percentile is zero. In that case, the barplots simply show the sites for which the density is zero and those for which it is non-zero. If there are fewer than ten groups of bars, the groupings contain ten percent of the sites each except for the leftmost grouping which will contain all of the remaining sites.

Also note that the title of the plot contains clues as to its content; the prefix indicates the type of variable studied while the suffix indicates the window width in the number of bases. The p-value given is the result of fitting a cubic polynomial to the gene density values.

The following expression data and probe set were used for this report:

```
[1] "ledgf293TS-HU133Plus2"

[1] "HG-U133"

      coef     se    z          p
a)SB 0.204 0.0151 13.5  1.12e-41
b)PB 0.374 0.0168 22.3 3.81e-110
c)TC 0.417 0.0151 27.6 9.99e-168
```

Here are the results for expression density. First, we count just genes that are in the upper half.

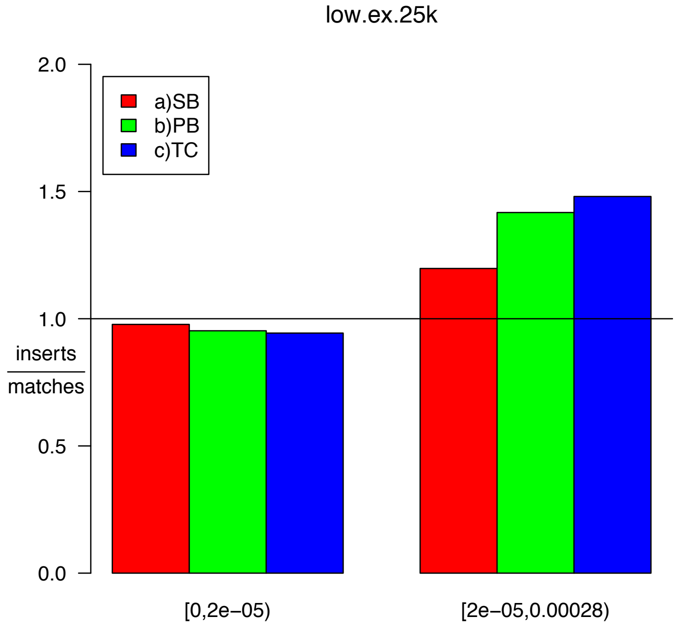

	coef	se	z	p
a)SB	0.223	0.0186	12.0	4.63e-33
b)PB	0.401	0.0203	19.7	9.03e-87
c)TC	0.464	0.0181	25.6	5.19e-144

Now we count genes in the upper $1/8^{th}$:

```
Density data too sparse for barplot

      coef     se     z          p
a)SB 0.235 0.0239  9.85  7.00e-23
b)PB 0.492 0.0255 19.30  4.63e-83
c)TC 0.521 0.0228 22.80 1.56e-115
```

And here we count genes in the upper $1/16^{th}$:

```
Density data too sparse for barplot

      coef     se     z        p
a)SB 0.245 0.0316  7.76 8.63e-15
b)PB 0.585 0.0328 17.90 2.66e-71
c)TC 0.603 0.0291 20.70 4.84e-95
```

Here the effect of density of CpG islands is studied:

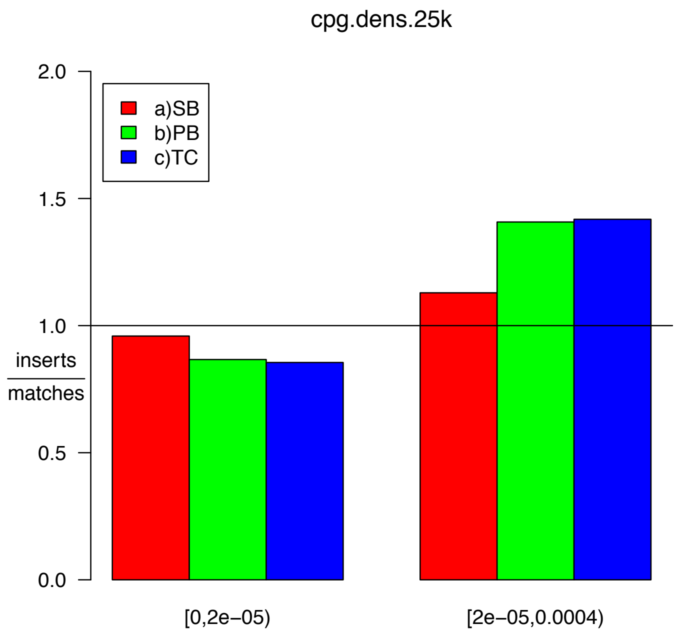

	coef	se	z	p
a)SB	0.164	0.0151	10.9	1.68e-27
b)PB	0.486	0.0164	29.7	3.11e-194
c)TC	0.507	0.0148	34.3	6.51e-258

4.2 50 kilobase Window

In the barplot that follows we examine the association of insertion sites with expression density in a 50 kilobase window surrounding each locus. First, we count just the number of genes represented on the chip.

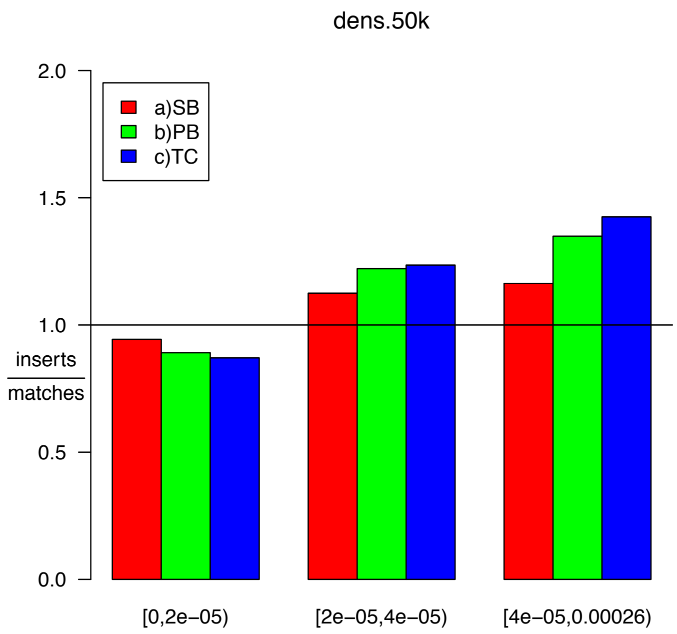

	coef	se	z	p
a)SB	0.234	0.0135	17.3	3.84e-67
b)PB	0.420	0.0153	27.4	1.88e-165
c)TC	0.456	0.0139	32.7	1.72e-234

Here are the results for expression density. First, we count just genes that are in the upper half.

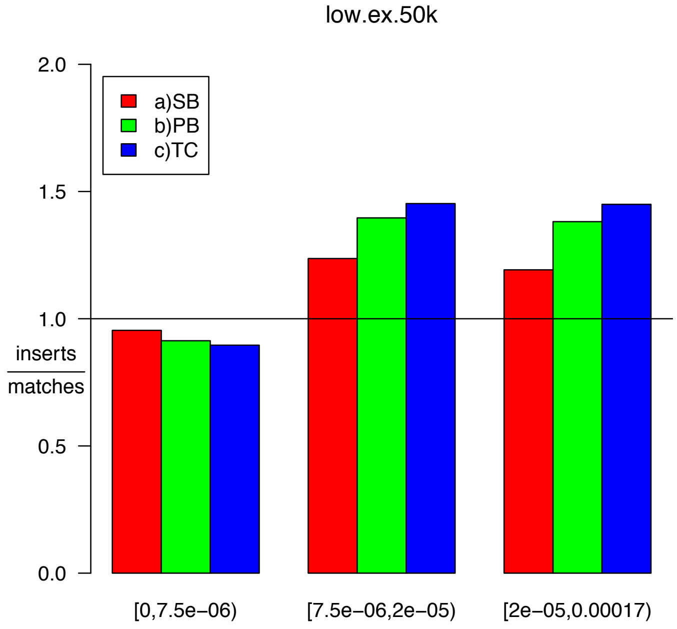

	coef	se	z	p
a)SB	0.256	0.0153	16.7	9.65e-63
b)PB	0.439	0.0170	25.9	1.89e-147
c)TC	0.490	0.0153	32.0	5.64e-225

Now we count genes in the upper $1/8^{th}$:

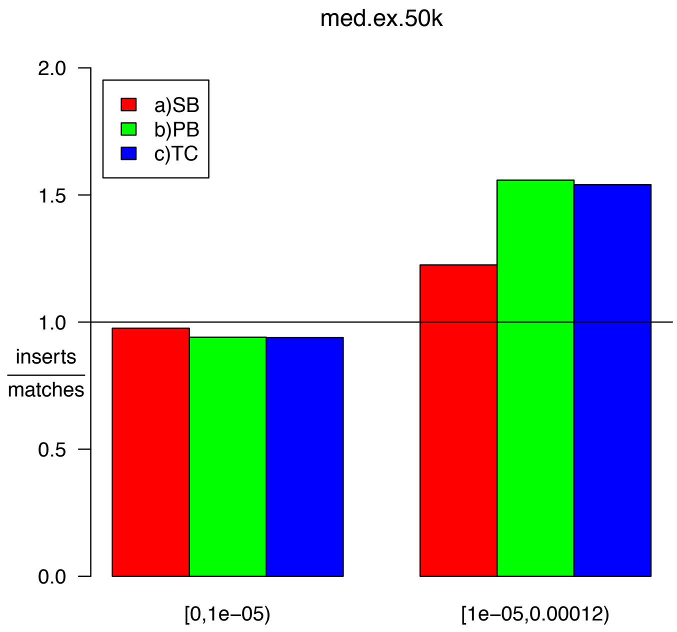


```
      coef     se    z          p
a)SB 0.240 0.0187 12.9  8.31e-38
b)PB 0.505 0.0202 25.1 1.60e-138
c)TC 0.493 0.0182 27.1 1.64e-161
```

And here we count genes in the upper $1/16^{th}$:

```
Density data too sparse for barplot

      coef     se    z          p
a)SB 0.218 0.0239  9.1  8.94e-20
b)PB 0.530 0.0251 21.1  3.87e-99
c)TC 0.520 0.0226 23.0 3.33e-117
```

Here the effect of density of CpG islands is studied:

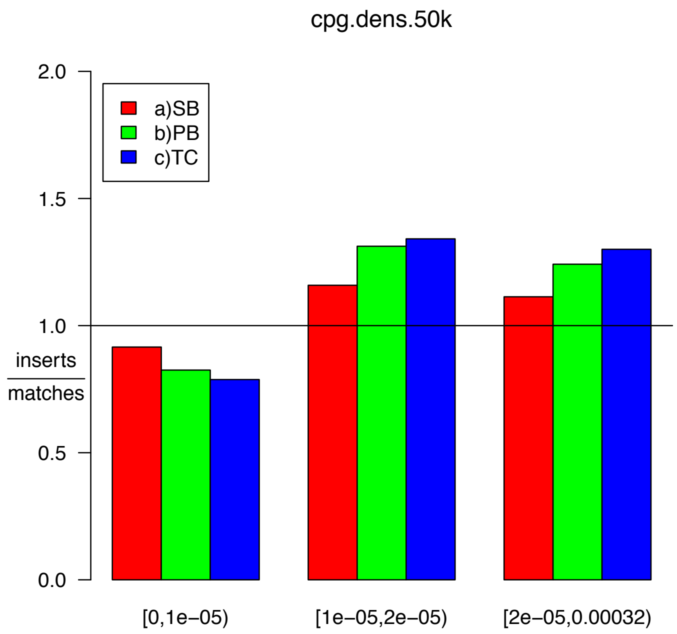


```
      coef     se    z          p
a)SB 0.218 0.0135 16.2  8.18e-59
b)PB 0.439 0.0152 28.8 1.13e-182
c)TC 0.517 0.0140 37.1 5.09e-301
```

4.3 100 kilobase Window

In the barplot that follows we examine the association of insertion sites with expression density in a 100 kilobase window surrounding each locus. First, we count just the number of genes represented on the chip.

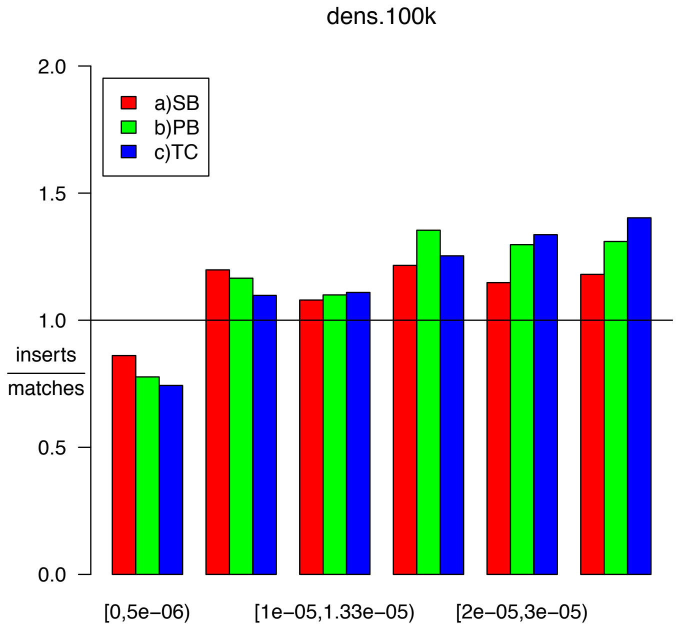

	coef	se	z	p
a)SB	0.261	0.0133	19.6	2.49e-85
b)PB	0.438	0.0153	28.6	1.40e-179
c)TC	0.502	0.0141	35.6	1.47e-277

Here are the results for expression density. First, we count just genes that are in the upper half.

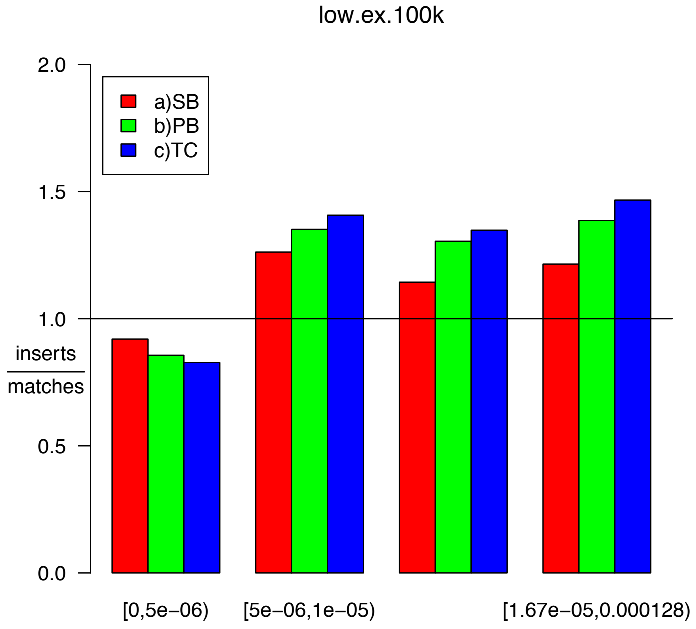

	coef	se	z	p
a)SB	0.290	0.0137	21.2	4.65e-100
b)PB	0.493	0.0154	31.9	5.86e-224
c)TC	0.560	0.0141	39.8	0.00e+00

Now we count genes in the upper $1/8^{th}$:

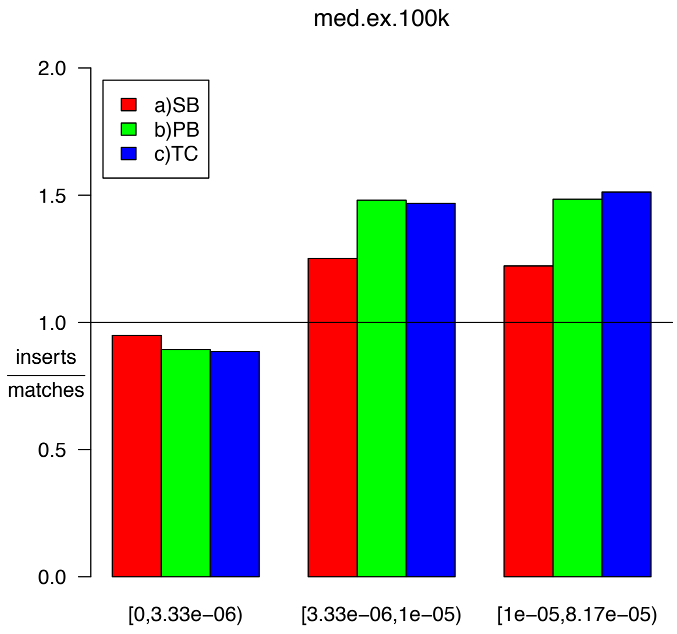

	coef	se	z	p
a)SB	0.274	0.0154	17.8	1.14e-70
b)PB	0.530	0.0170	31.3	2.16e-214
c)TC	0.550	0.0153	35.9	8.88e-283

And here we count genes in the upper $1/16^{th}$:

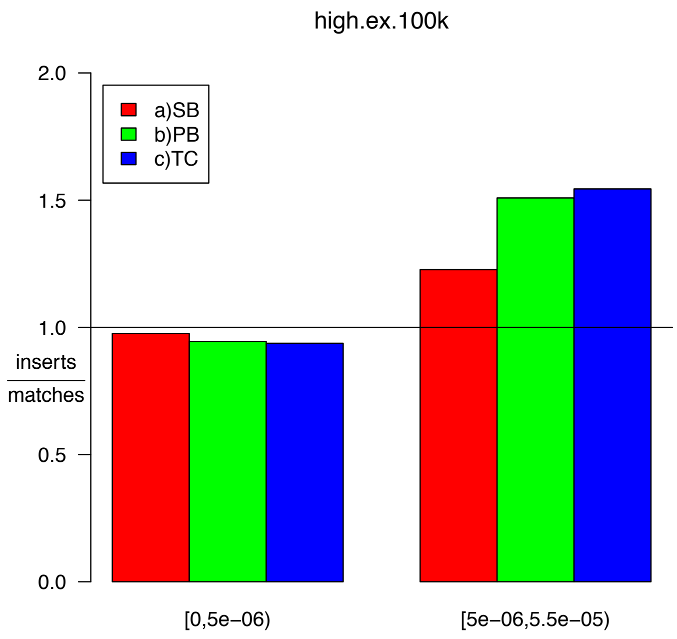

	coef	se	z	p
a)SB	0.245	0.0186	13.1	1.97e-39
b)PB	0.516	0.0200	25.9	2.02e-147
c)TC	0.532	0.0179	29.6	4.96e-193

Here the effect of density of CpG islands is studied:

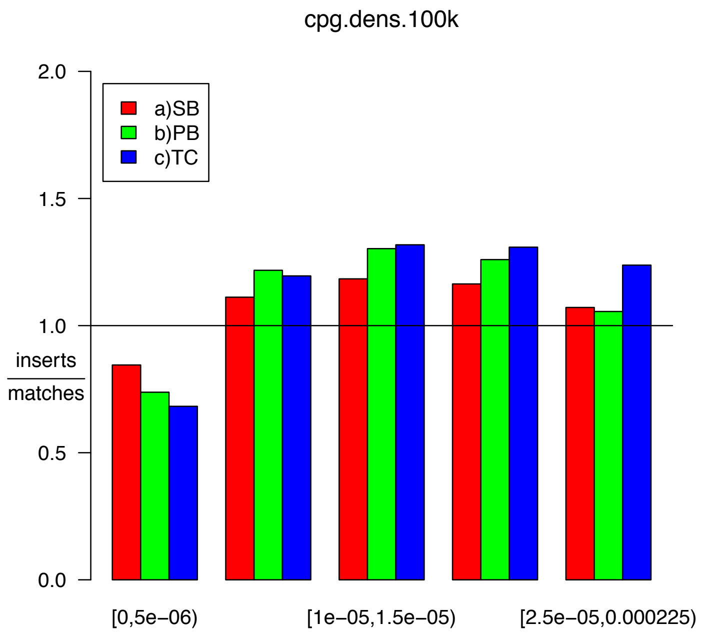

	coef	se	z	p
a)SB	0.215	0.0139	15.5	2.85e-54
b)PB	0.321	0.0156	20.6	4.82e-94
c)TC	0.422	0.0141	29.9	4.56e-197

4.4 250 kilobase Window

In the barplot that follows we examine the association of insertion sites with expression density in a 250 kilobase window surrounding each locus. First, we count just the number of genes represented on the chip.

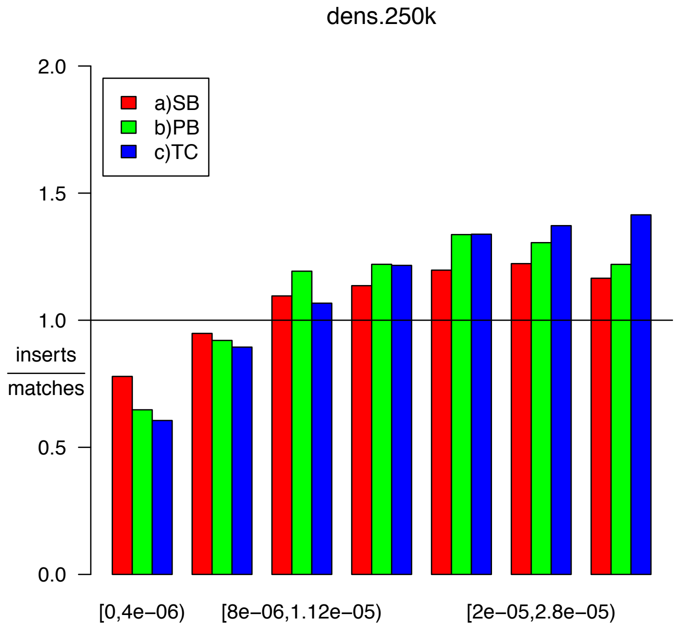

	coef	se	z	p
a)SB	0.300	0.0134	22.4	1.65e-111
b)PB	0.448	0.0153	29.3	3.77e-189
c)TC	0.546	0.0140	39.1	0.00e+00

Here are the results for expression density. First, we count just genes that are in the upper half.

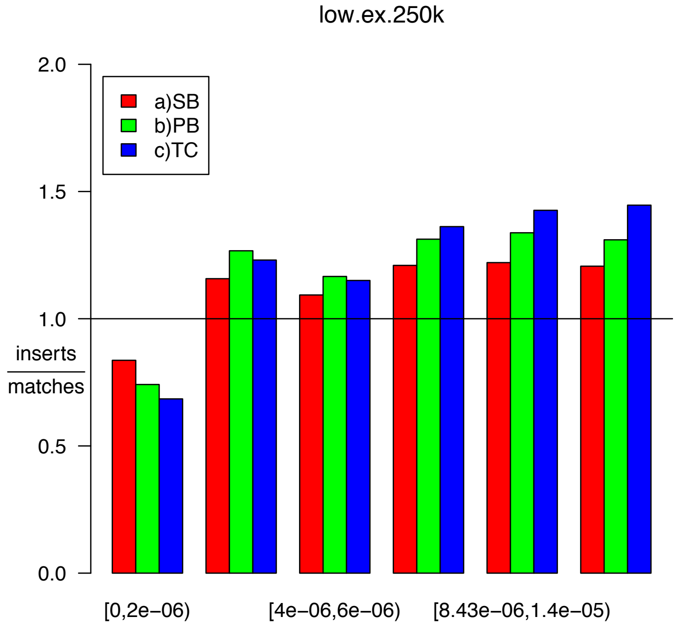

	coef	se	z	p
a)SB	0.317	0.0134	23.8	6.84e-125
b)PB	0.503	0.0154	32.7	5.07e-235
c)TC	0.615	0.0142	43.3	0.00e+00

Now we count genes in the upper $1/8^{th}$:

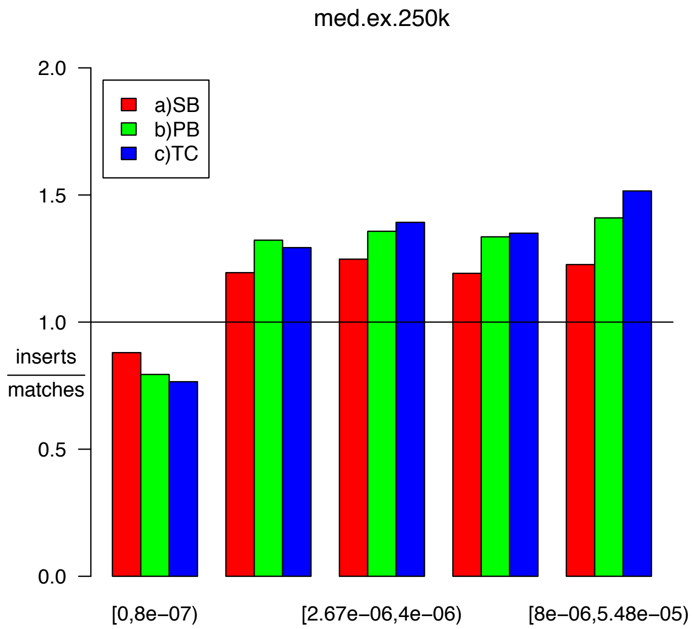

	coef	se	z	p
a)SB	0.322	0.0135	23.9	1.14e-126
b)PB	0.549	0.0153	35.9	1.11e-282
c)TC	0.605	0.0140	43.2	0.00e+00

And here we count genes in the upper $1/16^{th}$:

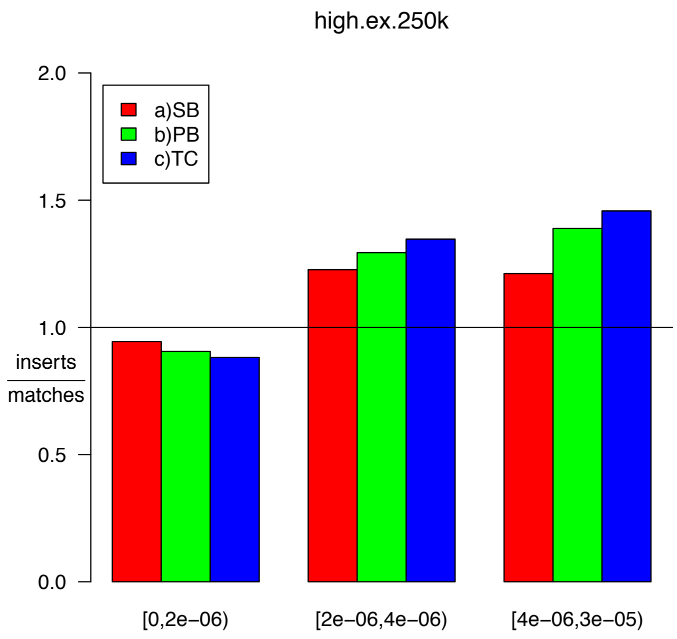

	coef	se	z	p
a)SB	0.282	0.0146	19.3	3.89e-83
b)PB	0.463	0.0162	28.5	7.83e-179
c)TC	0.505	0.0146	34.5	1.78e-260

Here the effect of density of CpG islands is studied:

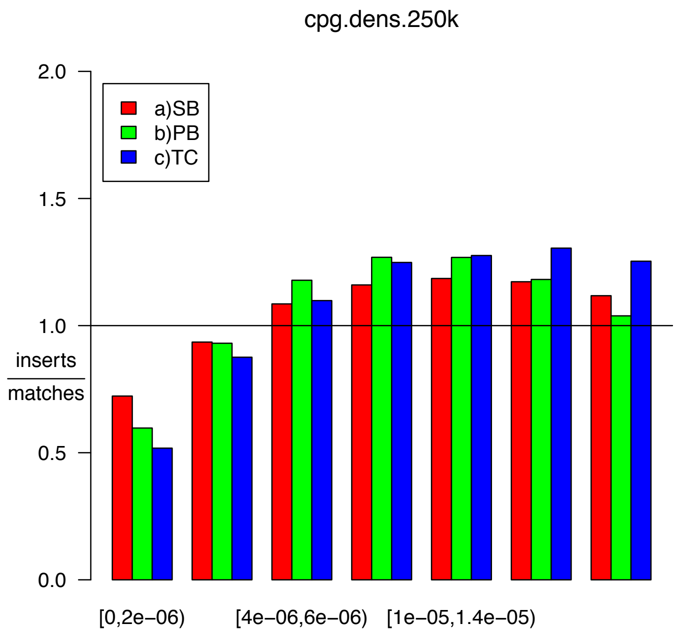

	coef	se	z	p
a)SB	0.292	0.0133	21.9	4.53e-106
b)PB	0.375	0.0152	24.6	9.57e-134
c)TC	0.514	0.0141	36.5	4.07e-291

4.5 500 kilobase Window

In the barplot that follows we examine the association of insertion sites with expression density in a 500 kilobase window surrounding each locus. First, we count just the number of genes represented on the chip.

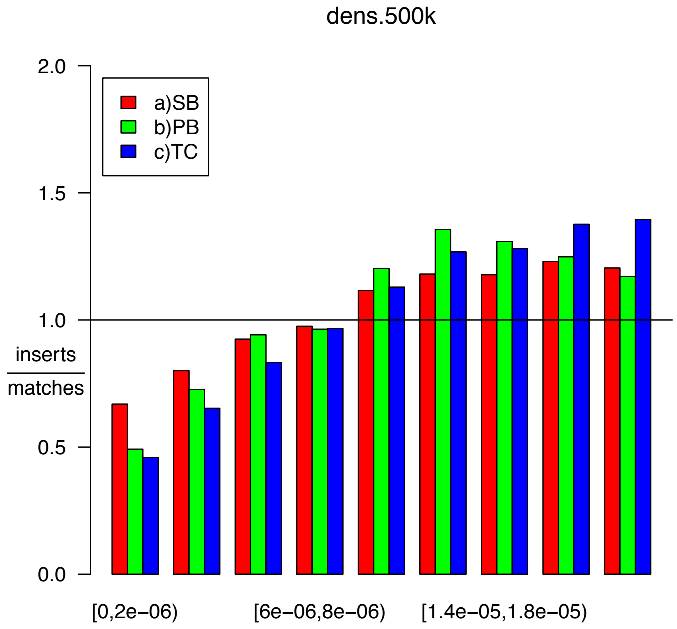

	coef	se	z	p
a)SB	0.343	0.0133	25.7	2.19e-145
b)PB	0.480	0.0153	31.3	7.10e-215
c)TC	0.588	0.0142	41.4	0.00e+00

Here are the results for expression density. First, we count just genes that are in the upper half.

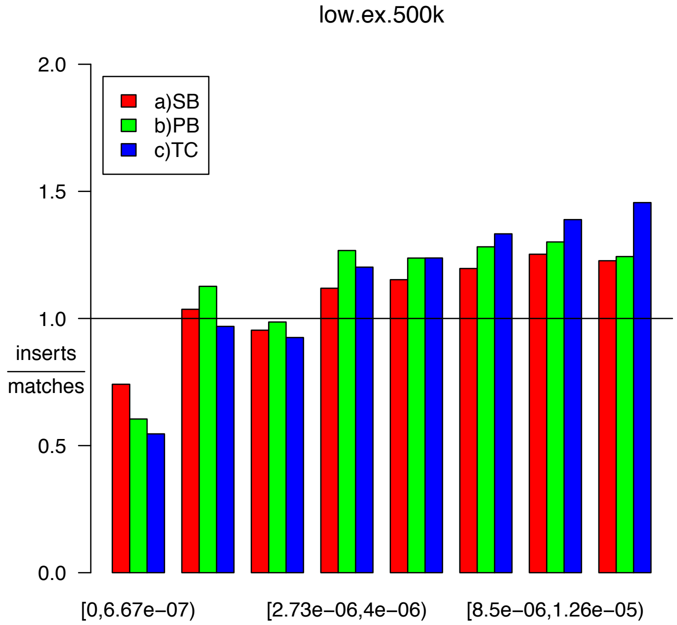


```
      coef     se    z          p
a)SB 0.360 0.0134 27.0 4.40e-160
b)PB 0.502 0.0154 32.6 1.68e-233
c)TC 0.645 0.0143 45.2  0.00e+00
```

Now we count genes in the upper $1/8^{th}$:

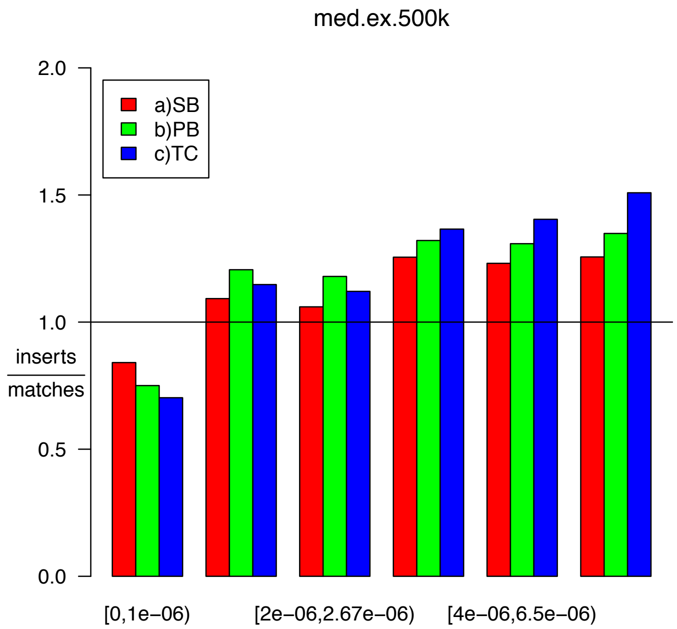


```
      coef     se    z          p
a)SB 0.323 0.0133 24.2 9.83e-130
b)PB 0.496 0.0153 32.4 1.75e-230
c)TC 0.589 0.0141 41.8  0.00e+00
```

And here we count genes in the upper $1/16^{th}$:

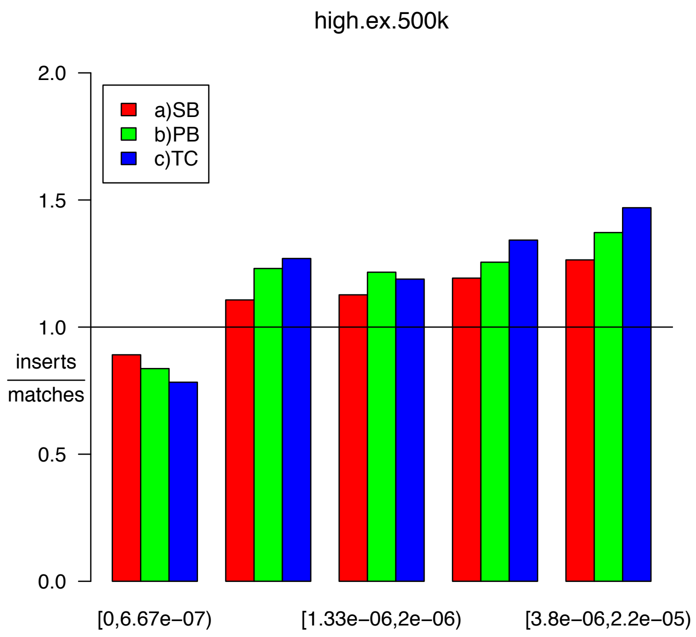


```
     coef     se    z          p
a)SB 0.297 0.0134 22.1 1.12e-108
b)PB 0.463 0.0152 30.4 3.01e-203
c)TC 0.558 0.0140 40.0  0.00e+00
```

Here the effect of density of CpG islands is studied:

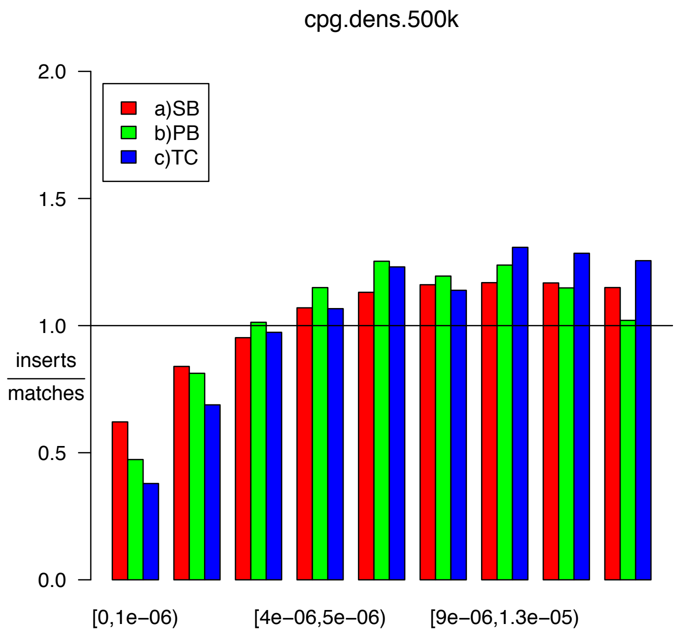

	coef	se	z	p
a)SB	0.288	0.0133	21.6	8.69e-104
b)PB	0.318	0.0152	20.9	9.13e-97
c)TC	0.473	0.0140	33.7	8.28e-249

4.6 1 megabase Window

In the barplot that follows we examine the association of insertion sites with expression density in a 1 megabase window surrounding each locus. First, we count just the number of genes represented on the chip.

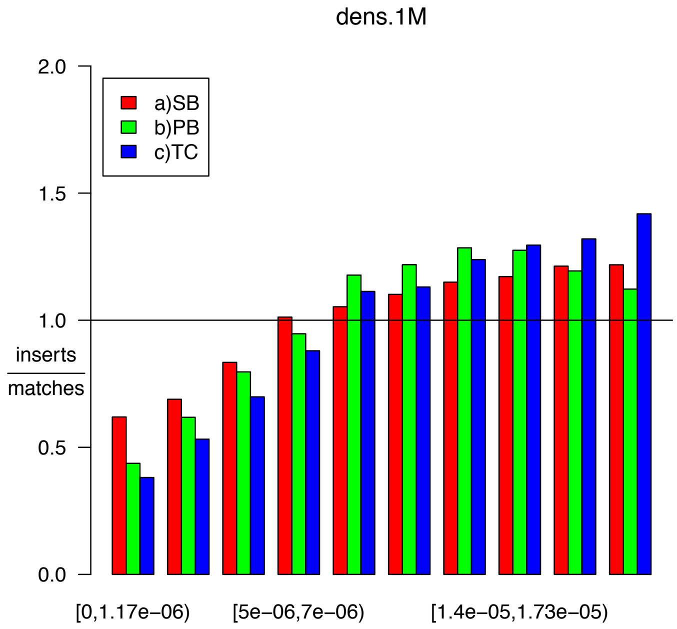

	coef	se	z	p
a)SB	0.320	0.0133	24.1	3.99e-128
b)PB	0.400	0.0153	26.2	5.57e-151
c)TC	0.549	0.0141	38.9	0.00e+00

Here are the results for expression density. First, we count just genes that are in the upper half.

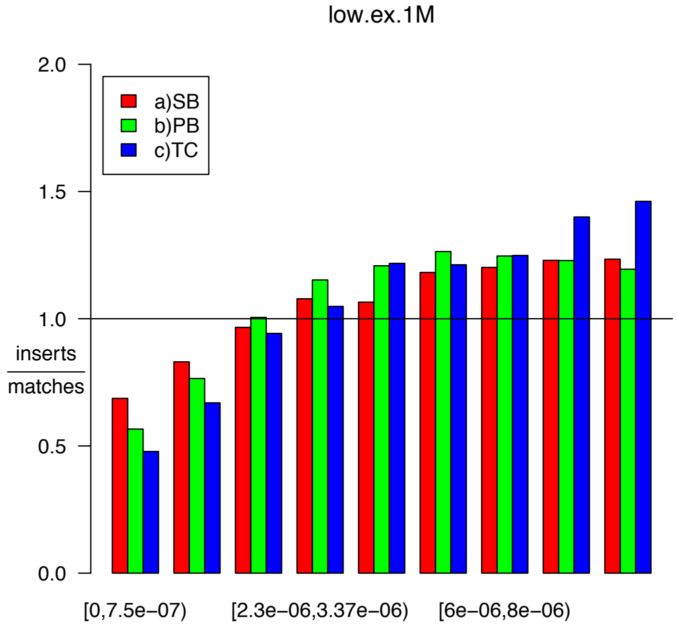

	coef	se	z	p
a)SB	0.339	0.0134	25.4	4.39e-142
b)PB	0.430	0.0153	28.1	2.66e-173
c)TC	0.607	0.0143	42.6	0.00e+00

Now we count genes in the upper $1/8^{th}$:

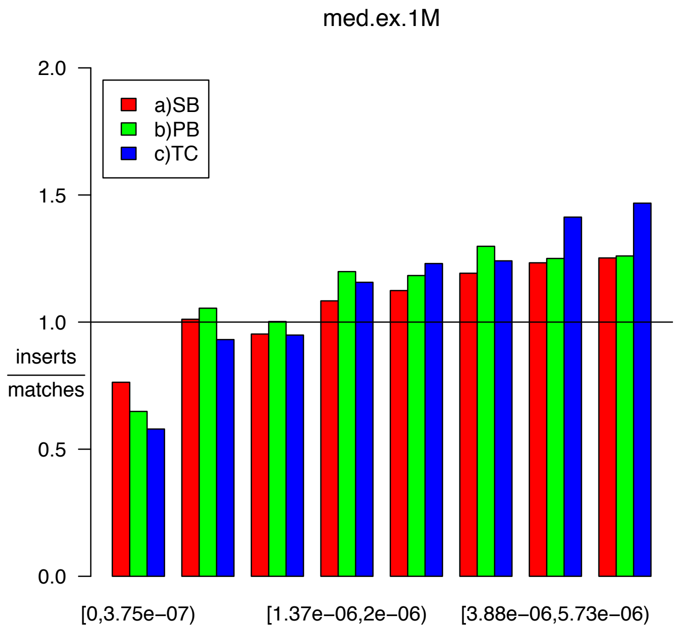


```
      coef     se    z          p
a)SB 0.334 0.0134 25.0 1.29e-137
b)PB 0.447 0.0153 29.2 5.53e-187
c)TC 0.597 0.0143 41.9  0.00e+00
```

And here we count genes in the upper $1/16^{th}$:

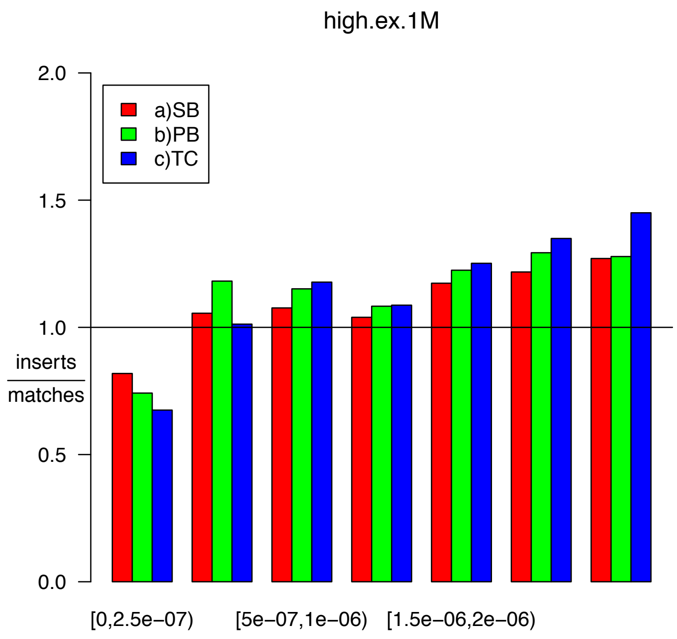

	coef	se	z	p
a)SB	0.295	0.0133	22.1	3.13e-108
b)PB	0.392	0.0152	25.7	4.39e-146
c)TC	0.525	0.0141	37.3	2.16e-304

Here the effect of density of CpG islands is studied:

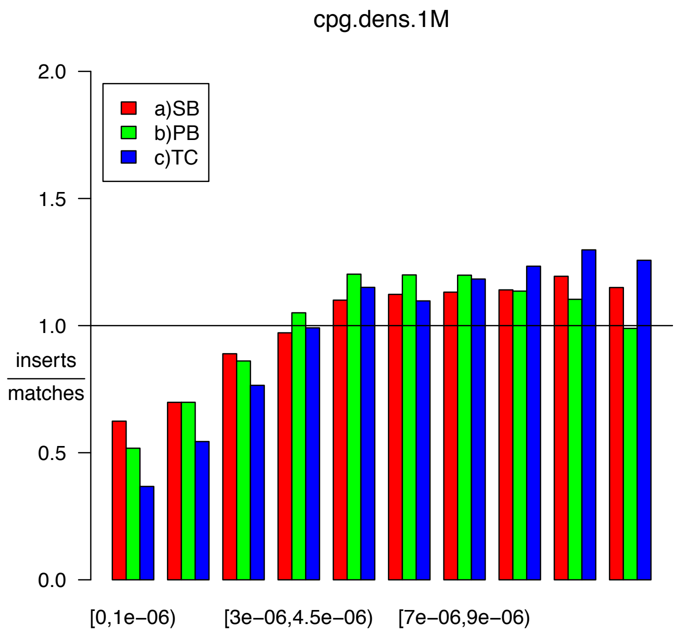

	coef	se	z	p
a)SB	0.271	0.0133	20.3	1.04e-91
b)PB	0.220	0.0152	14.5	1.69e-47
c)TC	0.425	0.0140	30.3	2.66e-202

4.7 2 megabase Window

In the barplot that follows we examine the association of insertion sites with expression density in a 2 megabase window surrounding each locus. First, we count just the number of genes represented on the chip.

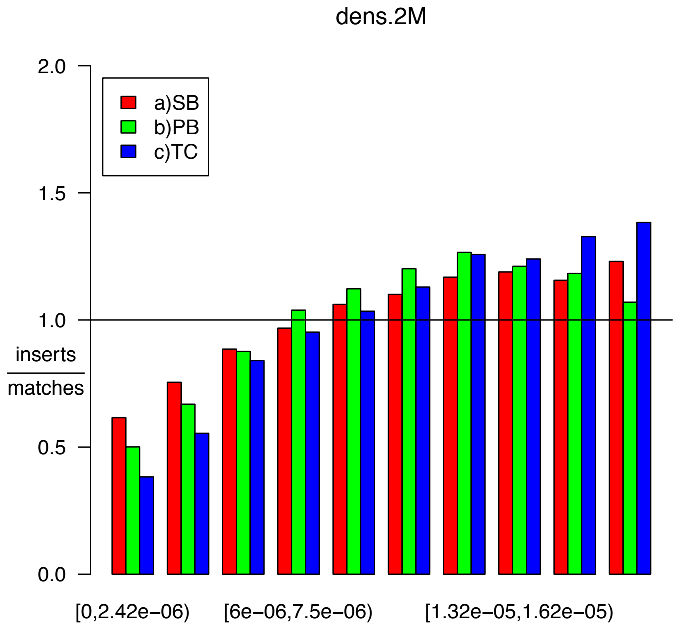

	coef	se	z	p
a)SB	0.319	0.0134	23.9	5.49e-126
b)PB	0.360	0.0153	23.6	8.66e-123
c)TC	0.533	0.0141	37.7	0.00e+00

Here are the results for expression density. First, we count just genes that are in the upper half.

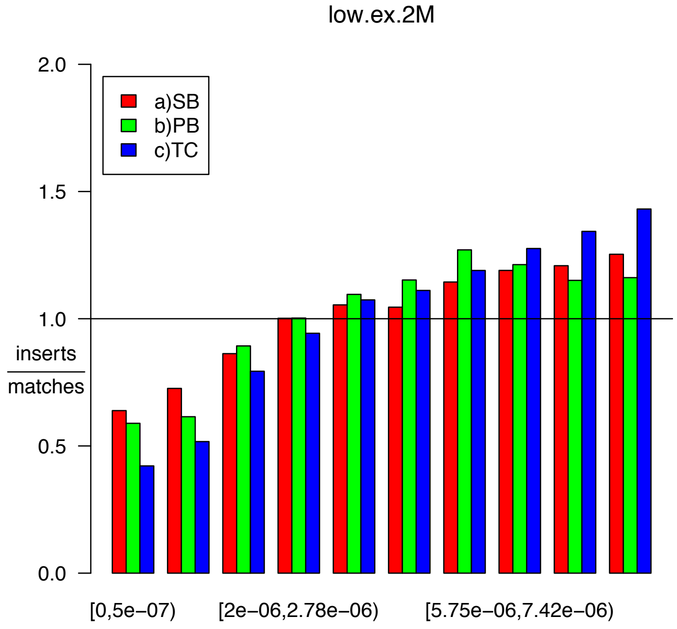


```
      coef     se    z          p
a)SB 0.314 0.0134 23.5 3.31e-122
b)PB 0.360 0.0153 23.6 8.12e-123
c)TC 0.537 0.0142 37.9  0.00e+00
```

Now we count genes in the upper $1/8^{th}$:

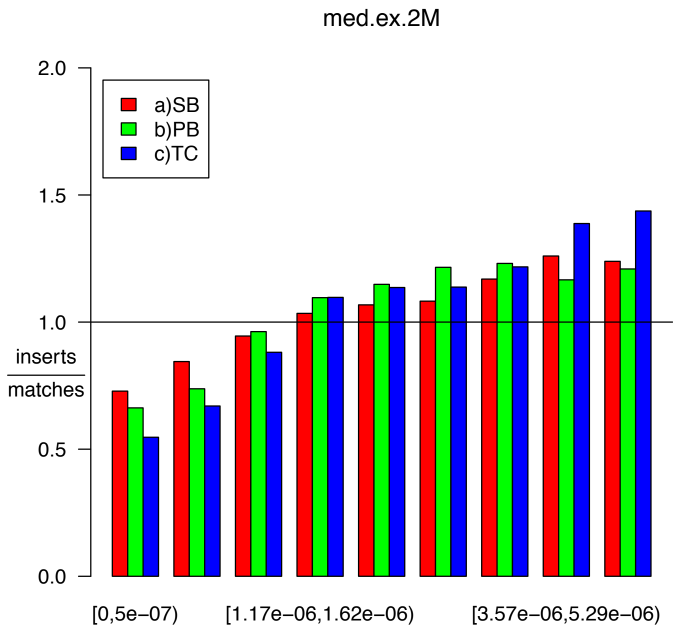

	coef	se	z	p
a)SB	0.308	0.0134	23.1	9.46e-118
b)PB	0.368	0.0153	24.1	2.06e-128
c)TC	0.511	0.0141	36.2	5.51e-287

And here we count genes in the upper $1/16^{th}$:

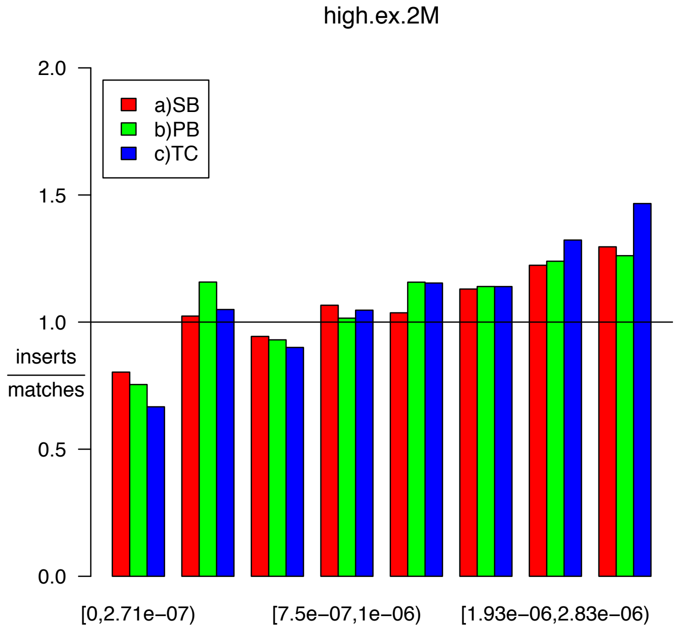

	coef	se	z	p
a)SB	0.281	0.0133	21.1	1.03e-98
b)PB	0.331	0.0152	21.8	2.36e-105
c)TC	0.477	0.0140	33.9	2.01e-252

Here the effect of density of CpG islands is studied:

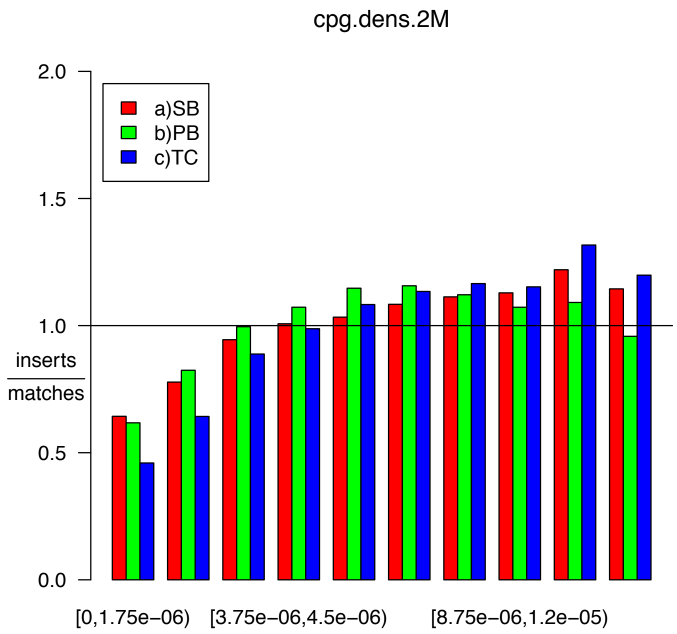

	coef	se	z	p
a)SB	0.270	0.0133	20.20	6.68e-91
b)PB	0.143	0.0151	9.42	4.50e-21
c)TC	0.375	0.0139	26.90	1.49e-159

4.8 4 megabase Window

In the barplot that follows we examine the association of insertion sites with expression density in a 4 megabase window surrounding each locus. First, we count just the number of genes represented on the chip.

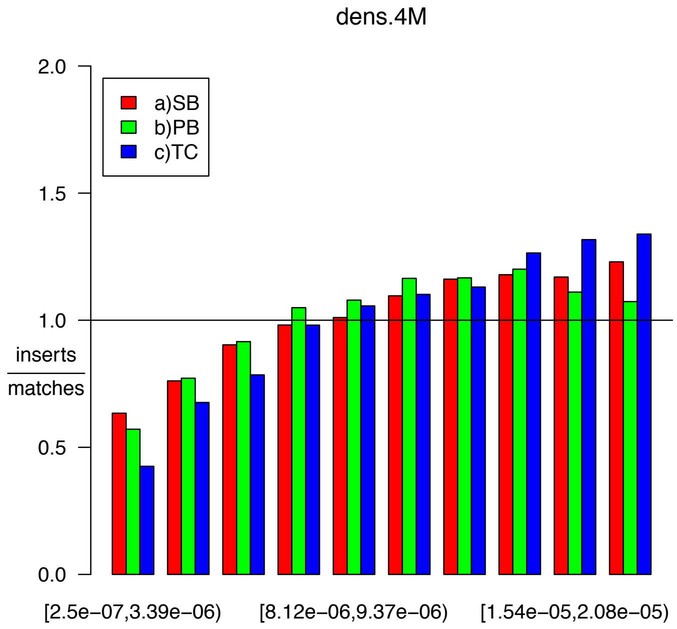

	coef	se	z	p
a)SB	0.316	0.0134	23.6	1.51e-123
b)PB	0.272	0.0152	17.9	1.97e-71
c)TC	0.457	0.0141	32.5	1.67e-231

Here are the results for expression density. First, we count just genes that are in the upper half.

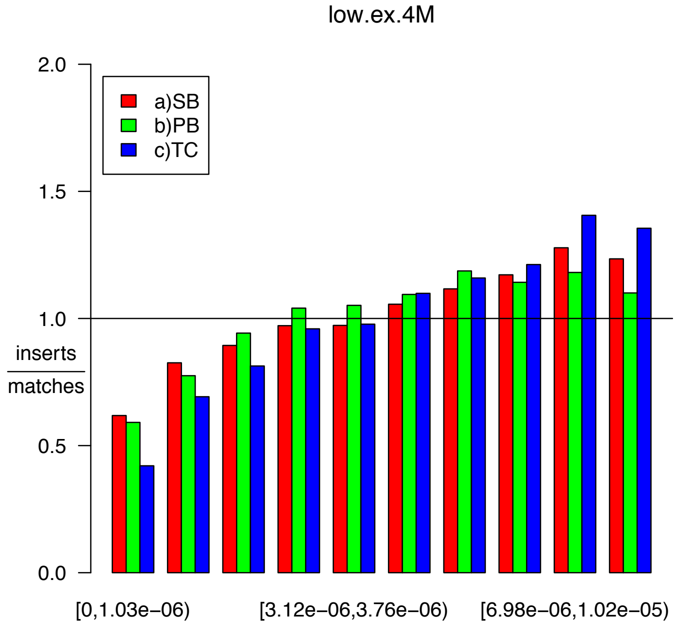

	coef	se	z	p
a)SB	0.321	0.0134	24.0	3.00e-127
b)PB	0.271	0.0152	17.8	1.09e-70
c)TC	0.489	0.0141	34.7	1.88e-263

Now we count genes in the upper $1/8^{th}$:

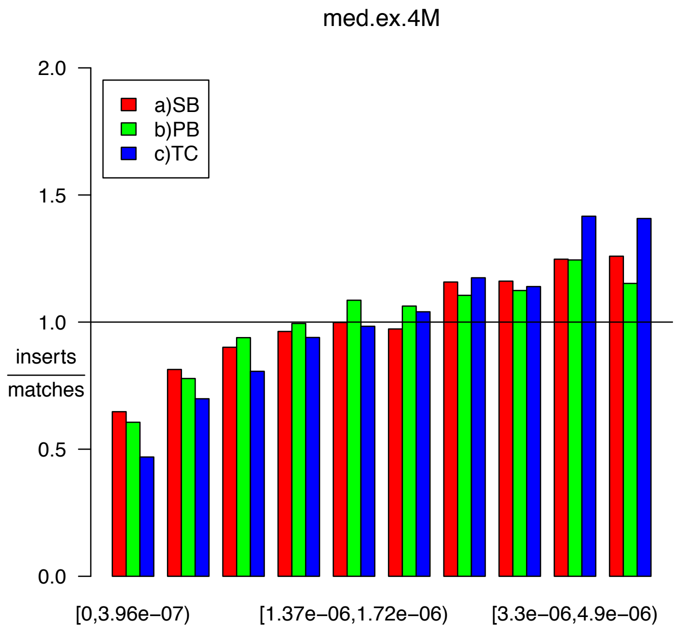


```
      coef     se    z          p
a)SB 0.296 0.0134 22.2 8.20e-109
b)PB 0.260 0.0152 17.1  7.65e-66
c)TC 0.466 0.0141 33.1 5.86e-240
```

And here we count genes in the upper $1/16^{th}$:

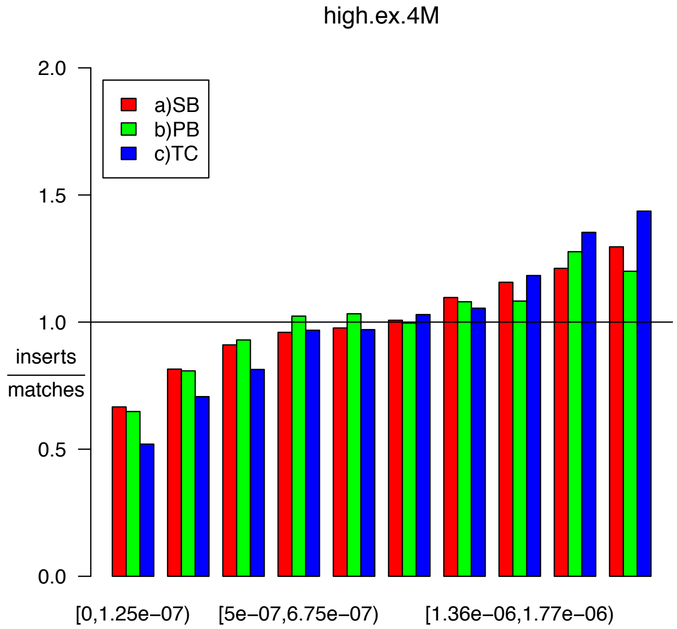

	coef	se	z	p
a)SB	0.285	0.0133	21.4	2.49e-101
b)PB	0.238	0.0152	15.7	1.45e-55
c)TC	0.421	0.0140	30.0	5.45e-198

Here the effect of density of CpG islands is studied:

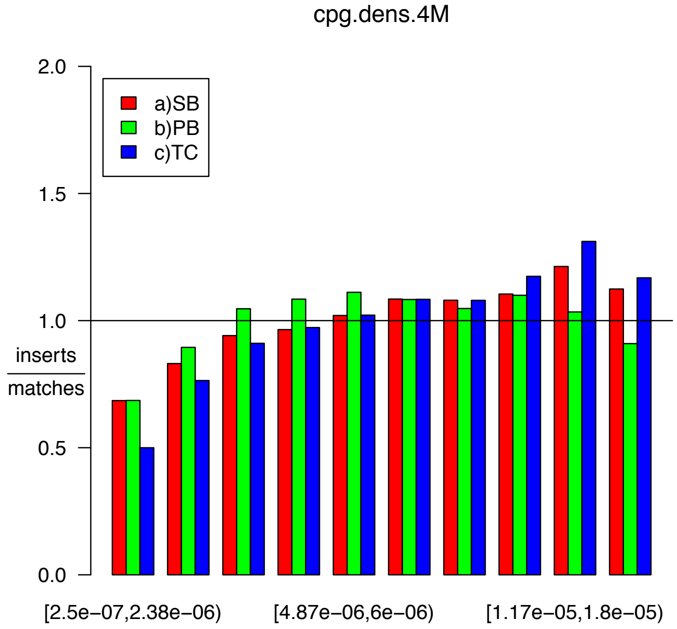

	coef	se	z	p
a)SB	0.233	0.0133	17.50	2.55e-68
b)PB	0.073	0.0151	4.82	1.43e-06
c)TC	0.331	0.0139	23.80	8.14e-125

4.9 8 megabase Window

In the barplot that follows we examine the association of insertion sites with expression density in a 8 megabase window surrounding each locus. First, we count just the number of genes represented on the chip.

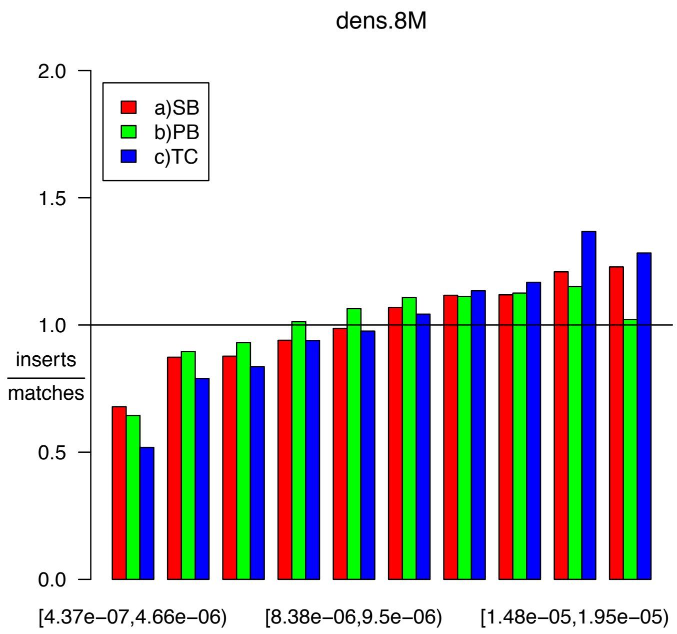

	coef	se	z	p
a)SB	0.280	0.0134	21.0	6.66e-98
b)PB	0.200	0.0152	13.2	8.49e-40
c)TC	0.395	0.0140	28.1	2.44e-174

Here are the results for expression density. First, we count just genes that are in the upper half.

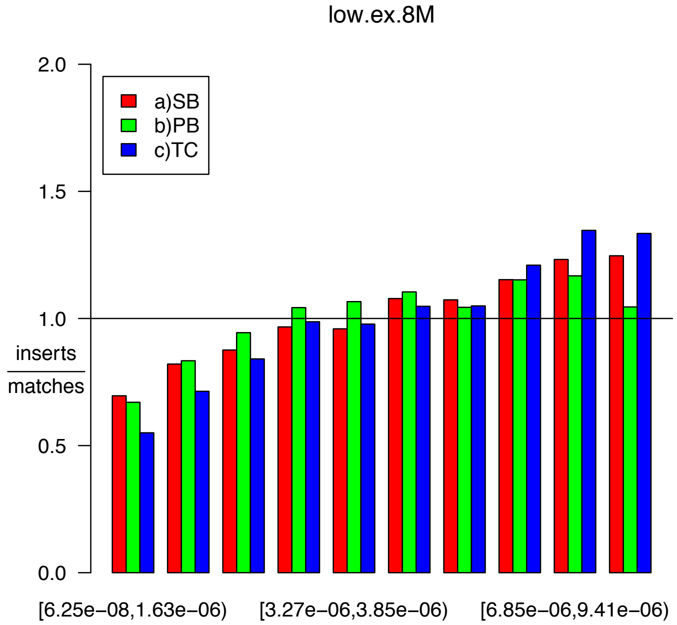

	coef	se	z	p
a)SB	0.295	0.0134	22.1	2.42e-108
b)PB	0.197	0.0151	13.0	1.42e-38
c)TC	0.393	0.0140	28.0	4.24e-173

Now we count genes in the upper $1/8^{th}$:

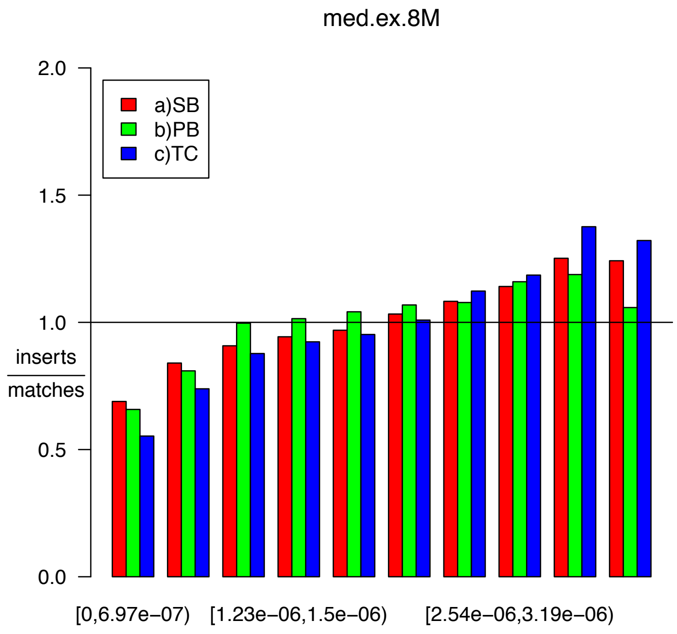

	coef	se	z	p
a)SB	0.283	0.0134	21.2	1.76e-99
b)PB	0.211	0.0152	13.9	5.72e-44
c)TC	0.402	0.0140	28.7	7.99e-181

And here we count genes in the upper $1/16^{th}$:

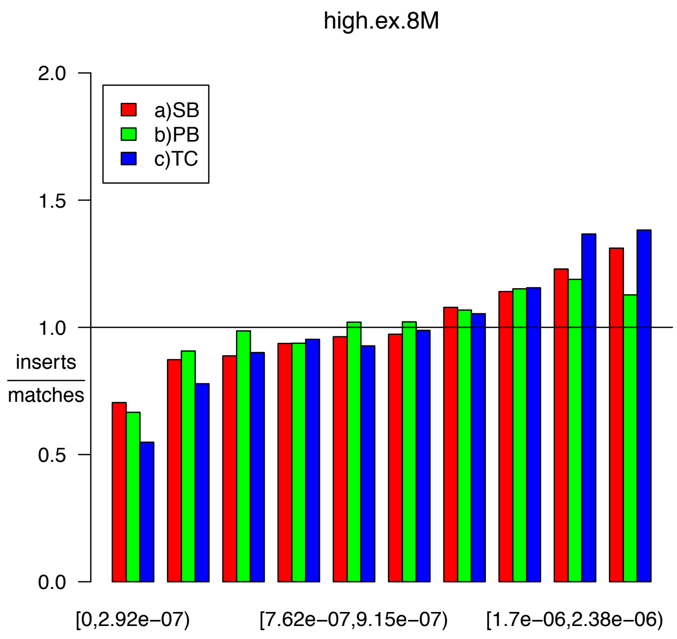

	coef	se	z	p
a)SB	0.270	0.0133	20.2	5.94e-91
b)PB	0.209	0.0151	13.8	2.42e-43
c)TC	0.375	0.0140	26.8	1.07e-157

Here the effect of density of CpG islands is studied:

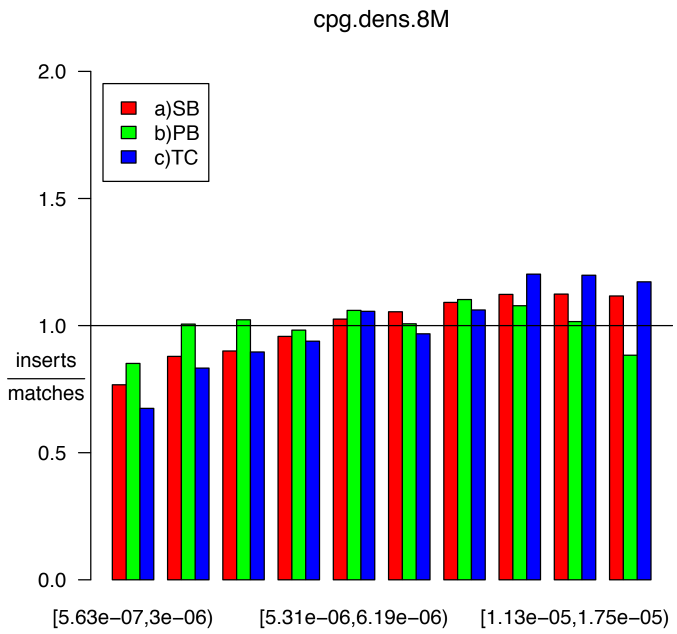

	coef	se	z	p
a)SB	0.197	0.0133	14.80	1.25e-49
b)PB	0.033	0.0151	2.18	2.90e-02
c)TC	0.243	0.0139	17.50	2.90e-68

4.10 16 megabase Window

In the barplot that follows we examine the association of insertion sites with expression density in a 16 megabase window surrounding each locus. First, we count just the number of genes represented on the chip.

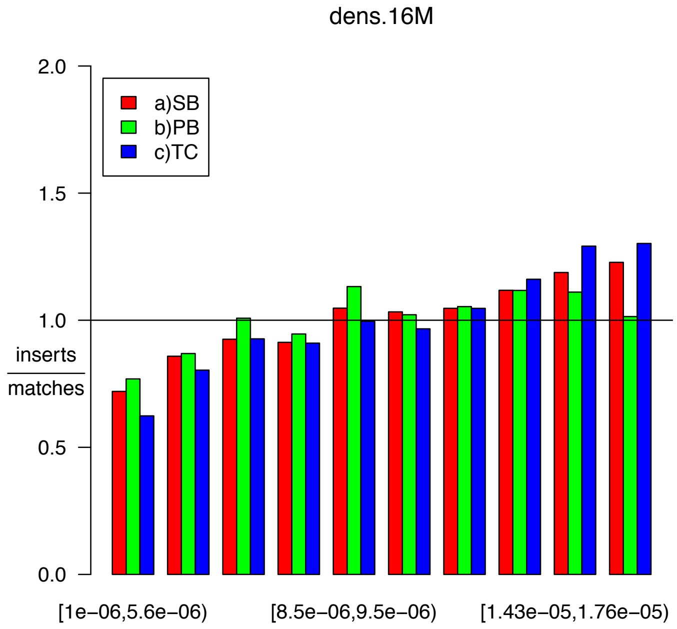

	coef	se	z	p
a)SB	0.231	0.0133	17.40	1.06e-67
b)PB	0.123	0.0151	8.12	4.83e-16
c)TC	0.306	0.0139	21.90	1.14e-106

Here are the results for expression density. First, we count just genes that are in the upper half.

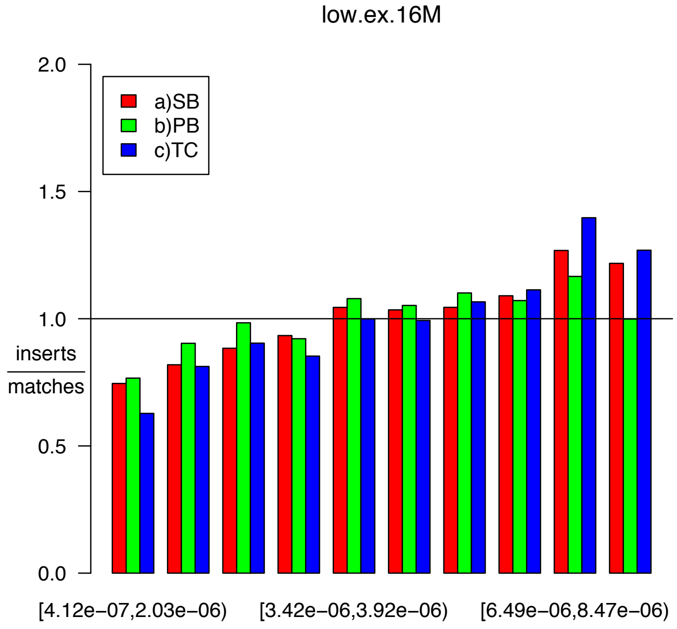

	coef	se	z	p
a)SB	0.247	0.0133	18.5	2.07e-76
b)PB	0.152	0.0151	10.0	1.14e-23
c)TC	0.335	0.0140	23.9	1.04e-126

Now we count genes in the upper $1/8^{th}$:

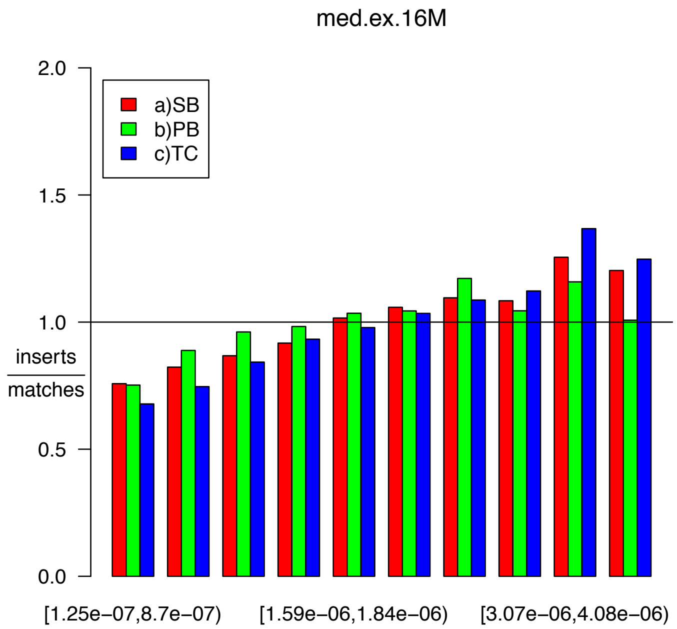

	coef	se	z	p
a)SB	0.263	0.0133	19.7	9.20e-87
b)PB	0.164	0.0151	10.8	2.05e-27
c)TC	0.341	0.0140	24.4	2.24e-131

And here we count genes in the upper $1/16^{th}$:

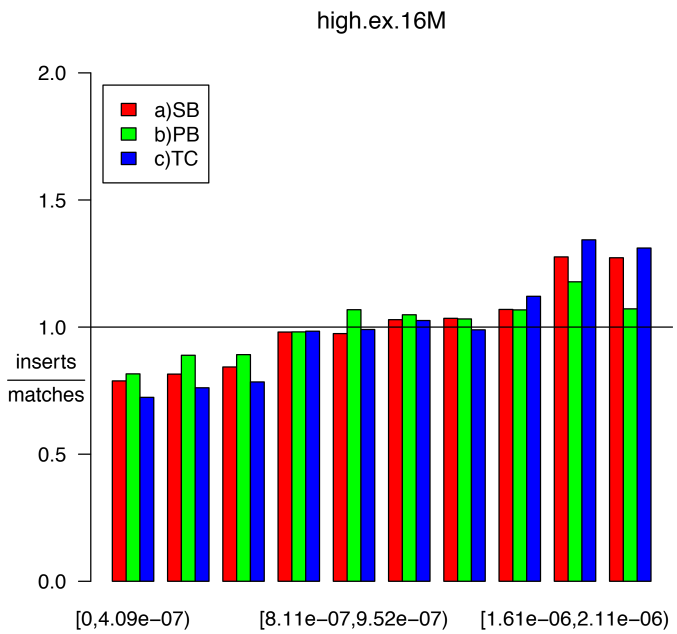

	coef	se	z	p
a)SB	0.255	0.0133	19.1	2.82e-81
b)PB	0.152	0.0151	10.0	1.06e-23
c)TC	0.315	0.0140	22.5	2.96e-112

Here the effect of density of CpG islands is studied:

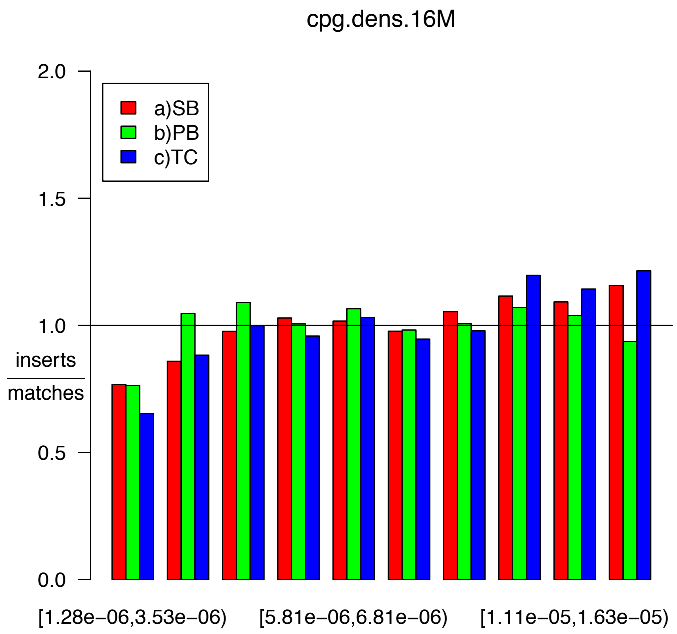

	coef	se	z	p
a)SB	0.1520	0.0133	11.40	3.15e-30
b)PB	0.0134	0.0151	0.89	3.74e-01
c)TC	0.1930	0.0139	13.90	4.26e-44

4.11 32 megabase Window

In the barplot that follows we examine the association of insertion sites with expression density in a 32 megabase window surrounding each locus. First, we count just the number of genes represented on the chip.

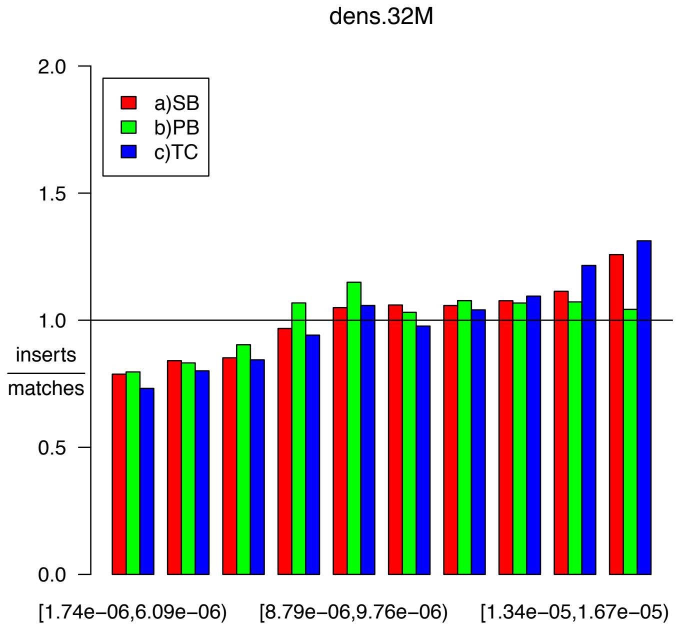

	coef	se	z	p
a)SB	0.214	0.0133	16.10	1.47e-58
b)PB	0.112	0.0151	7.44	1.01e-13
c)TC	0.257	0.0139	18.50	4.97e-76

Here are the results for expression density. First, we count just genes that are in the upper half.

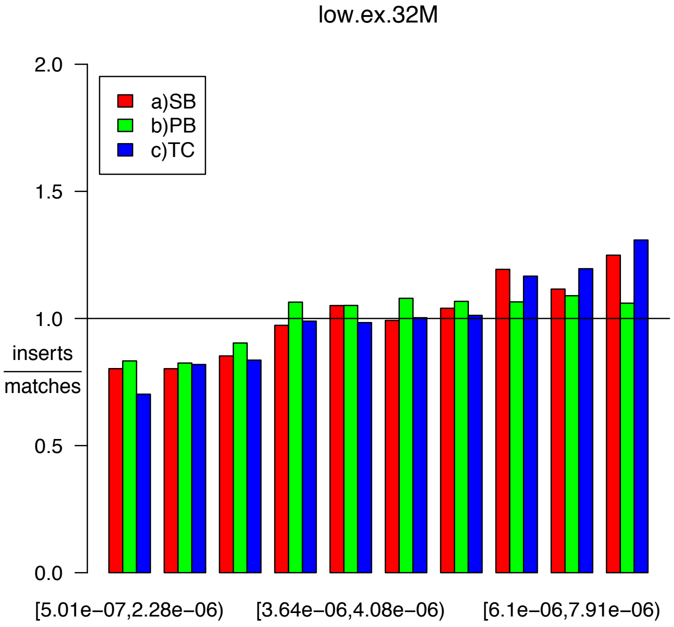

	coef	se	z	p
a)SB	0.222	0.0133	16.70	1.75e-62
b)PB	0.140	0.0151	9.25	2.28e-20
c)TC	0.276	0.0139	19.80	3.33e-87

Now we count genes in the upper $1/8^{th}$:

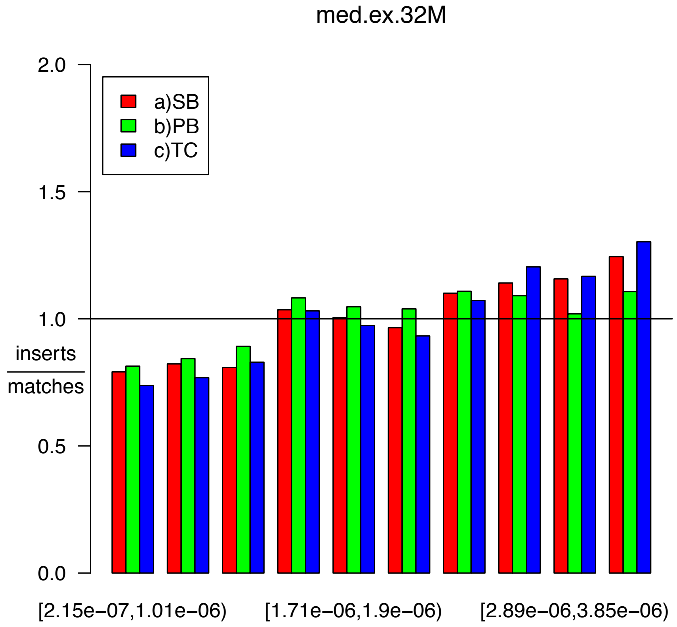

	coef	se	z	p
a)SB	0.229	0.0133	17.20	1.47e-66
b)PB	0.139	0.0151	9.24	2.53e-20
c)TC	0.271	0.0139	19.50	1.21e-84

And here we count genes in the upper $1/16^{th}$:

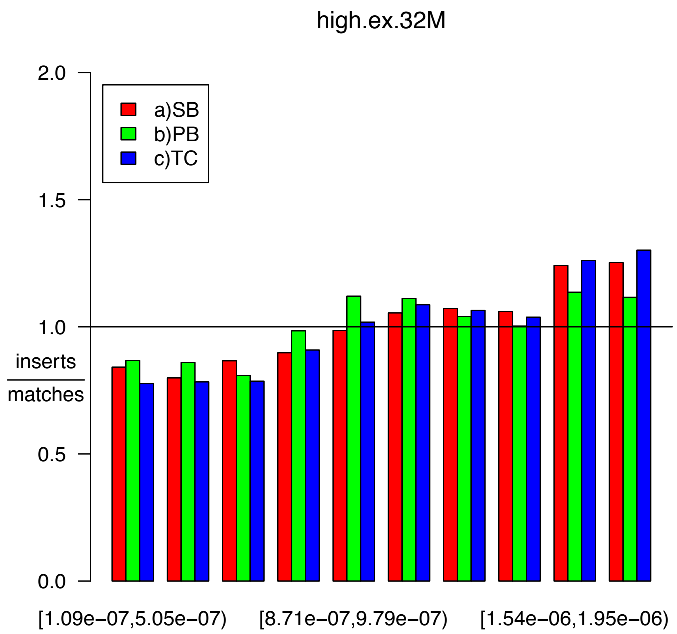

	coef	se	z	p
a)SB	0.257	0.0133	19.3	6.74e-83
b)PB	0.154	0.0151	10.2	2.27e-24
c)TC	0.300	0.0139	21.5	2.16e-102

Here the effect of density of CpG islands is studied:

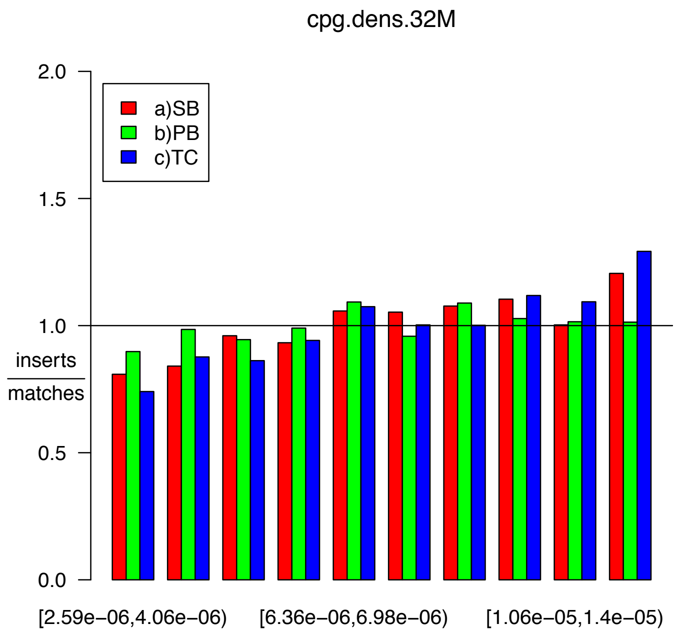

	coef	se	z	p
a)SB	0.168	0.0133	12.70	1.10e-36
b)PB	0.040	0.0151	2.65	7.96e-03
c)TC	0.206	0.0139	14.90	6.29e-50

5 Juxtaposition with Gene Start and End Positions

5.1 Acembly Annotations

In this section we study the effect of juxtaposition in terms of gene start and end positions. The first barplot shows the effect of gene width for those insertions that are located within an Acembly gene. The table following the barplot shows the p-values for a test of the hypothesis that the proportions in each of the categories that define the bars are equal in the insertions and their matches. This p-value is obtained from the $5 \times 2 \times k$ table of counts defined by gene width category, insertion/match status, and stratum (consisting of an insertion and its matched sites) using a likelihood ratio test for the hypothesis of no association between gene width category and insertion/match status. The test used compared the log-linear model [1] with all two-way configurations to that with no gene width category and insertion/match status configuration.

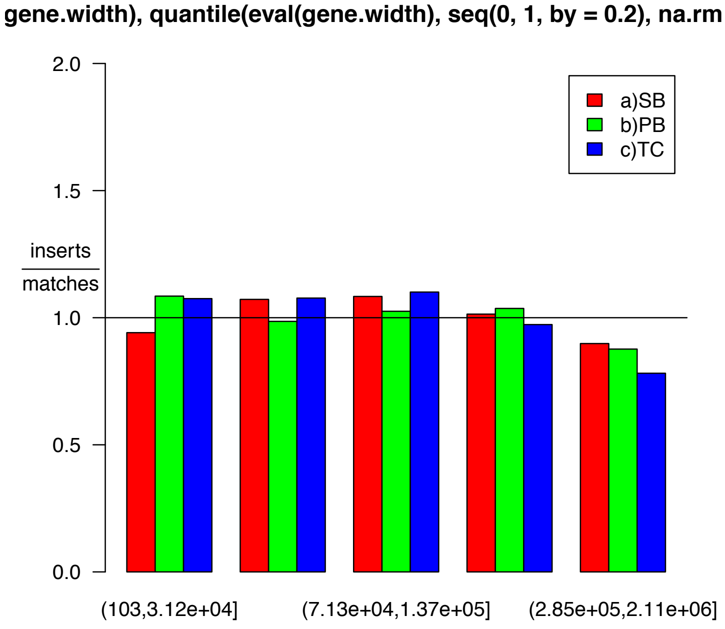


```
    a)SB      b)PB      c)TC
2.62e-13 1.08e-14 1.45e-42
```

The next plot uses the width of a non-gene region for insertions that fall into such regions.

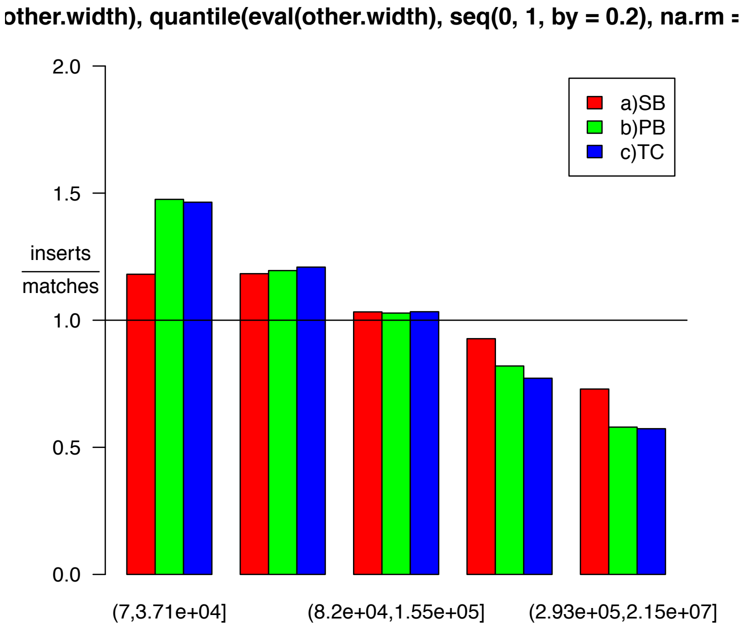


```
   a)SB       b)PB       c)TC
7.33e-63 5.95e-148 8.52e-199
```

The next plot studies the distance to the nearest boundary between a gene and a non-gene region. The distance is expressed as a fraction of the length of the region. Thus, '0.25' refers to one quarter of the distance from the site to nearest boundary divided by the total width of the region.

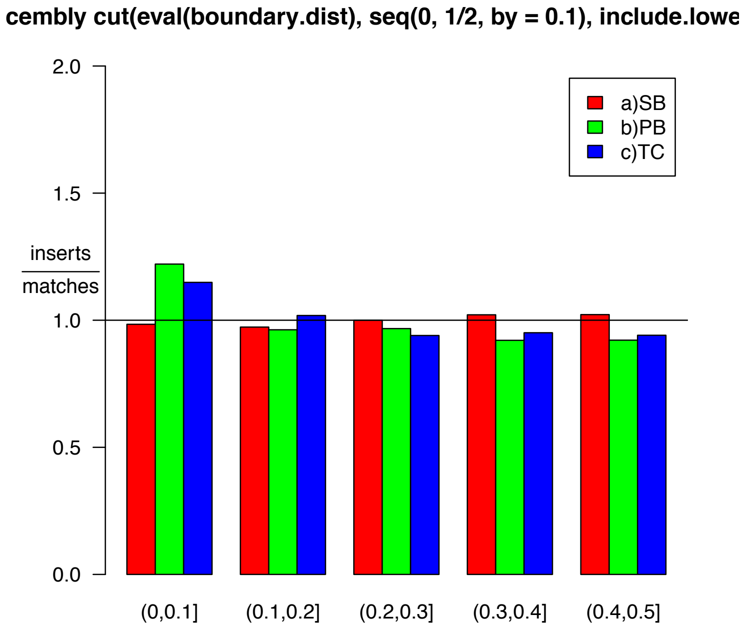


```
    a)SB     b)PB     c)TC
1.96e-02 2.26e-61 7.10e-37
```

This plot studies the effect of nearness to the beginning of a transcript. For sites in genes, it is the distance to the start of the gene divided by the width of the gene. For other sites it is the distance from the site to the nearer gene if that gene boundary is also a transcription starting point. Locations near '0' are relatively near the beginning of transcription, while those near '1' are near the termination of the transcript.

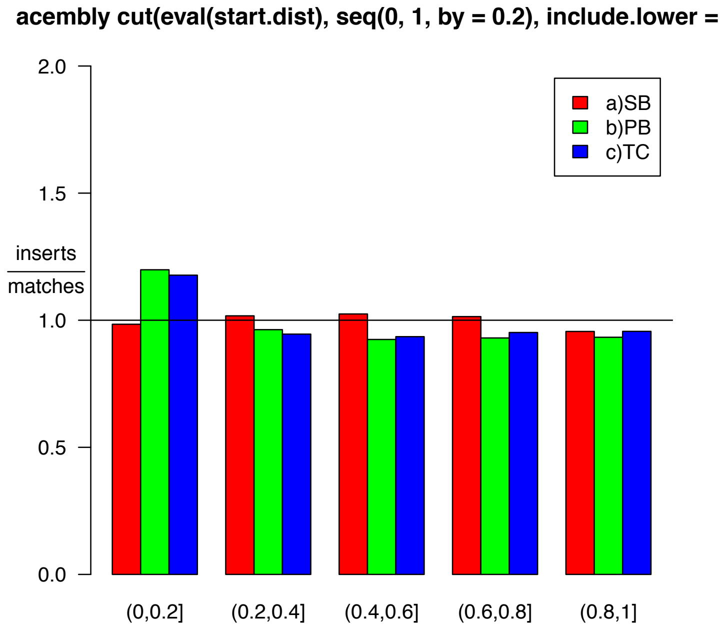


```
    a)SB      b)PB      c)TC
1.24e-03 3.68e-54 8.22e-48
```

5.2 RefSeq Annotations

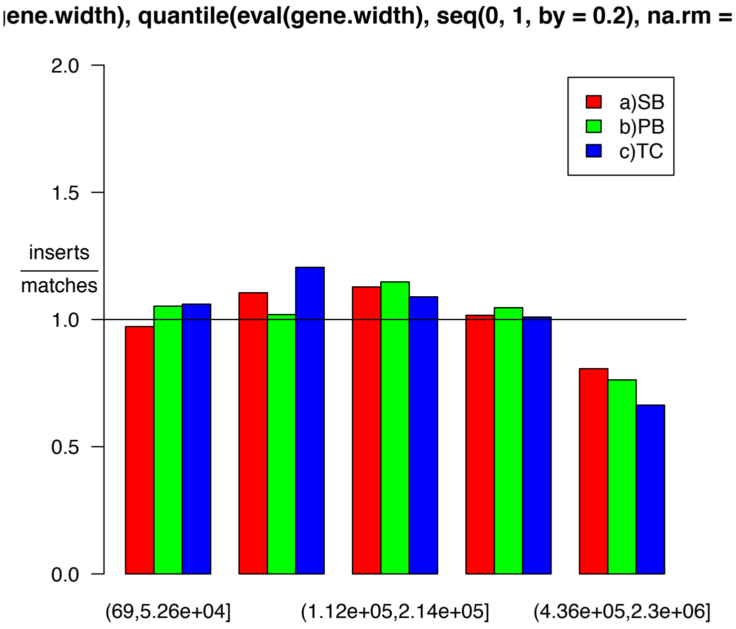


```
    a)SB      b)PB      c)TC
1.05e-34 1.12e-34 1.85e-78
```

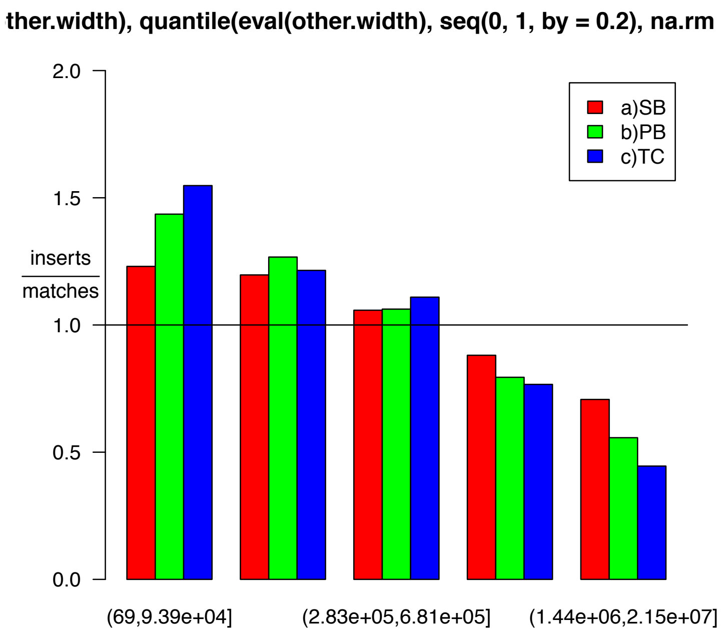


```
      a)SB       b)PB       c)TC
2.60e-128 2.16e-247  0.00e+00
```

refSeq cut(eval(boundary.dist), seq(0, 1/2, by = 0.1), include.lower

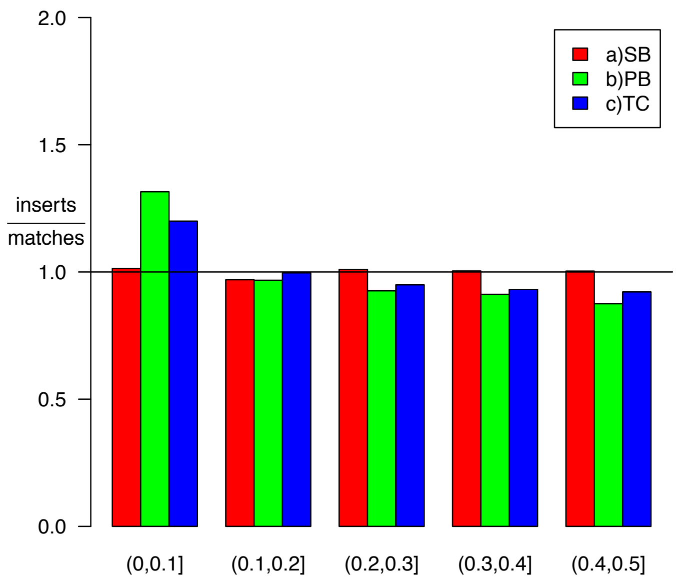


```
    a)SB       b)PB       c)TC
1.01e-01 6.31e-122  1.03e-60
```

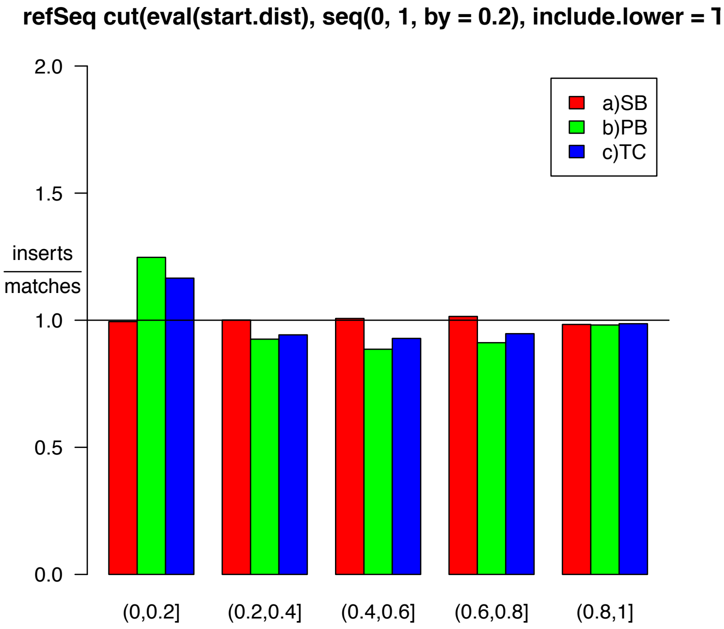


```
      a)SB      b)PB      c)TC
6.07e-01 9.91e-82 5.45e-42
```

5.3 genScan Annotations

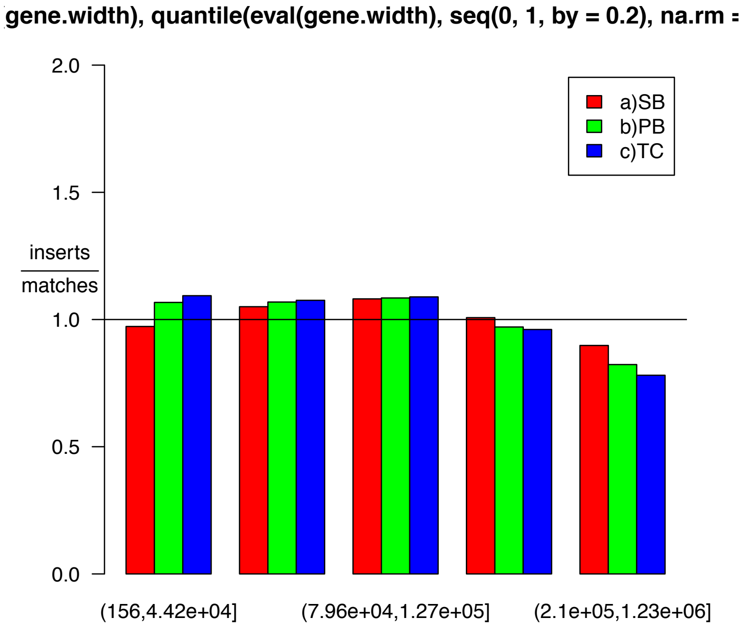


```
    a)SB      b)PB      c)TC
5.45e-18 1.17e-35 3.02e-56
```

other.width), quantile(eval(other.width), seq(0, 1, by = 0.2), na.rm =

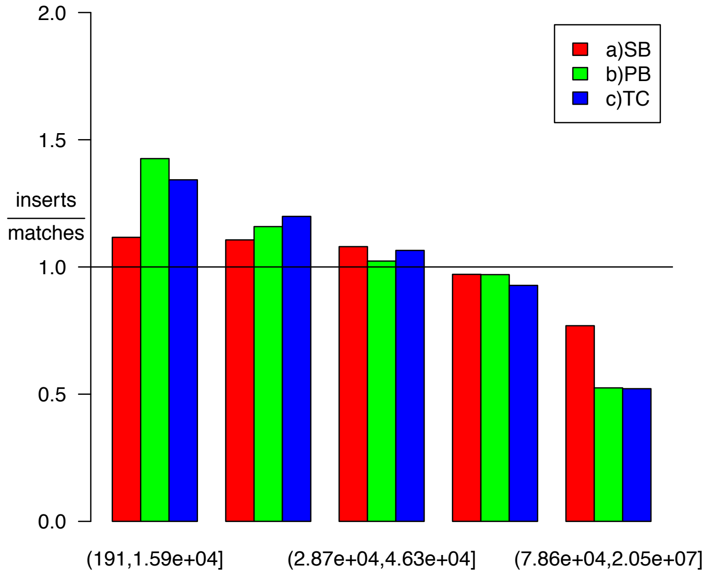


```
    a)SB       b)PB       c)TC
1.26e-23 2.99e-101 2.02e-106
```

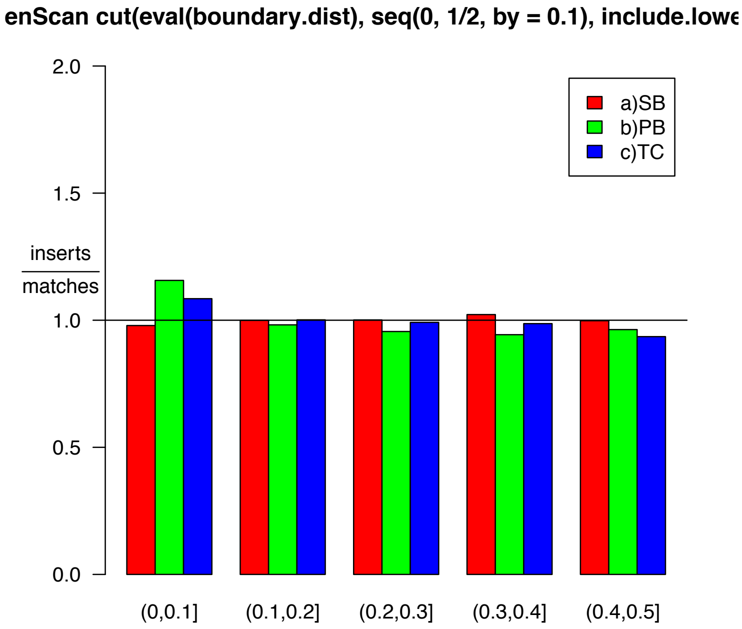


```
    a)SB      b)PB      c)TC
2.29e-01 1.19e-29 4.50e-13
```

genScan cut(eval(start.dist), seq(0, 1, by = 0.2), include.lower =

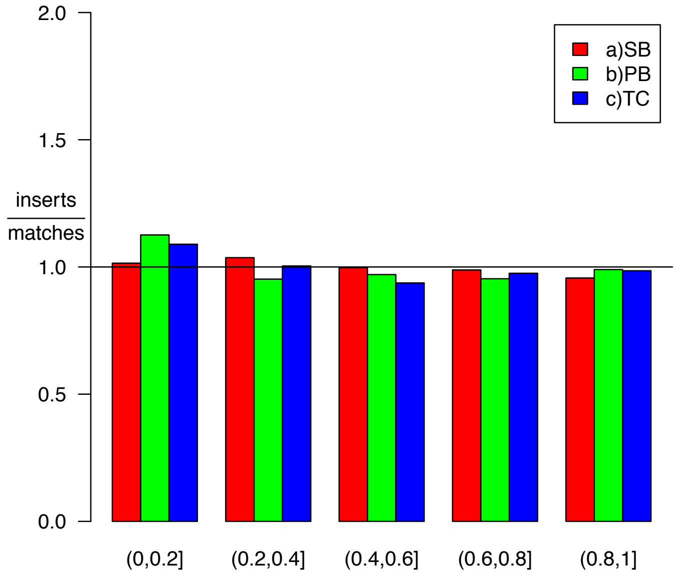


```
    a)SB      b)PB      c)TC
1.06e-03 1.83e-18 5.04e-13
```

5.4 uniGene Annotations

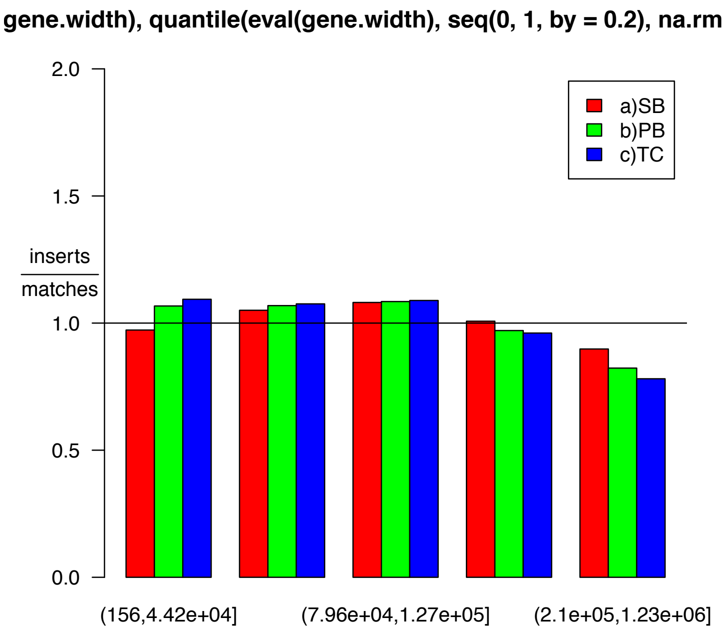


```
    a)SB      b)PB      c)TC
5.45e-18 1.17e-35 3.02e-56
```

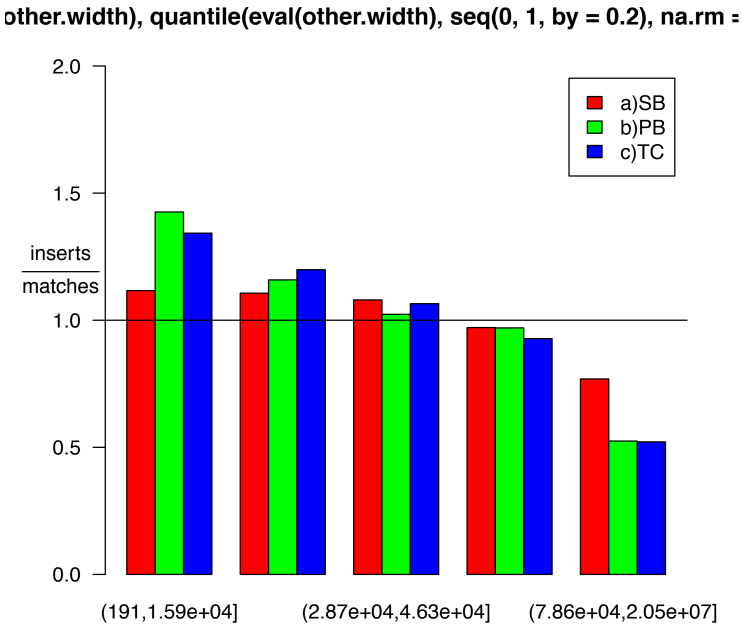


```
    a)SB       b)PB       c)TC
1.26e-23 2.99e-101 2.02e-106
```

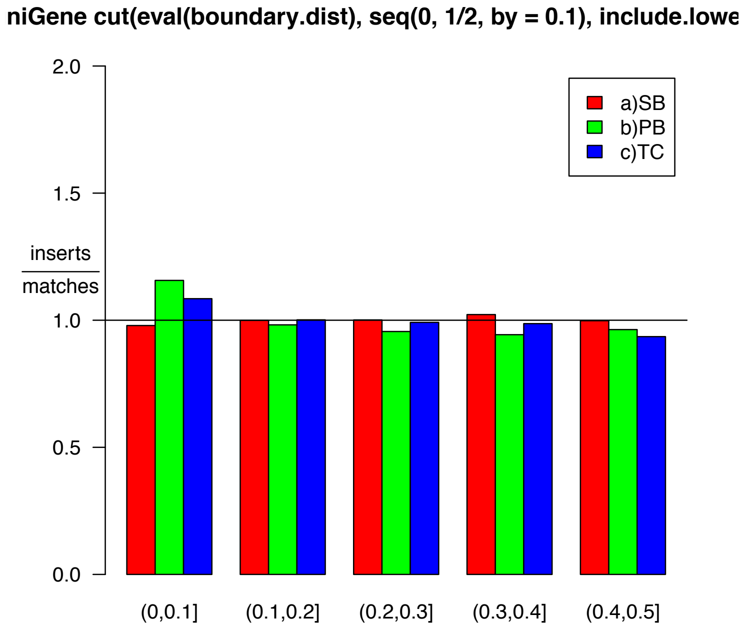


```
     a)SB      b)PB      c)TC
2.29e-01 1.19e-29 4.50e-13
```

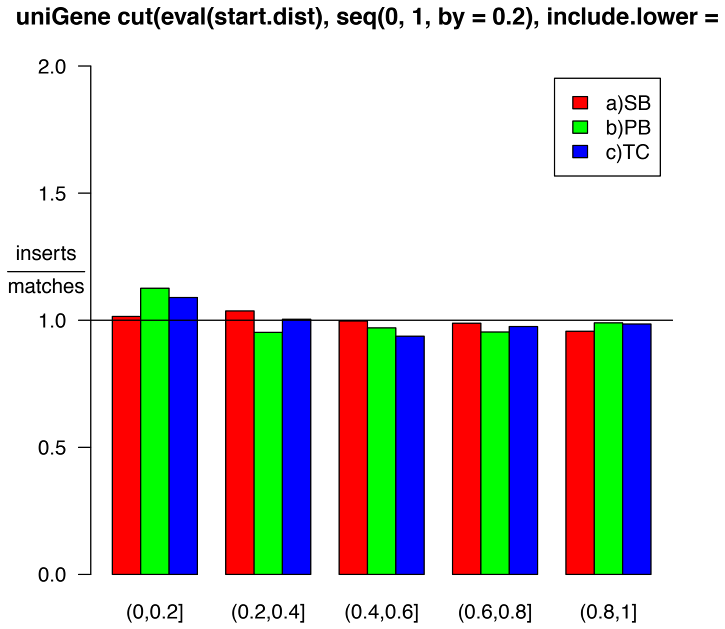


```
    a)SB      b)PB      c)TC
1.06e-03 1.83e-18 5.04e-13
```

6 GC content

Here we study the effect of GC content on insertion. The GC content is taken from the Human Genome Draft at GoldenPath from the table
`http://genome.ucsc.edu/goldenPath/hg18/database/gc5Base.txt.gz`.

Following the plot is a table of fitted coefficients based on splitting the GC percent data at the median.

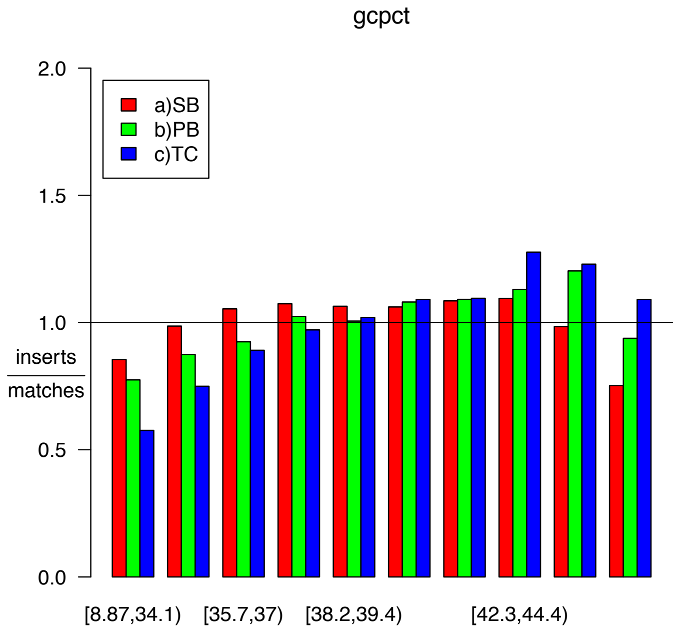


```
          coef     se      z         p
a)SB -0.00835 0.0134 -0.624  5.33e-01
b)PB  0.17100 0.0152 11.300  2.19e-29
c)TC  0.32100 0.0141 22.800 5.18e-115
```

References

[1] Yvonne M.M. Bishop, Stephen E. Fienberg, and Paul W. Holland. *Discrete multivariate analyses: Theory and practice* (MIT Press, 1975).

[2] P. McCullagh and John A. Nelder. *Generalized linear models.* (Chapman & Hall ltd, 1999).

[3] Xiaolin Wu, Yuan Li, Bruce Crise, Shawn M. Burgess "Transcription Start Regions in the Human Genome Are Favored Targets for MLV Integration," *Science,* **300**(5626), (June 2003): 1749-1751.
